# A Modular Synthesis of Teraryl‐Based α‐Helix Mimetics, Part 3: Iodophenyltriflate Core Fragments Featuring Side Chains of Proteinogenic Amino Acids

**DOI:** 10.1002/ejoc.202101278

**Published:** 2022-02-24

**Authors:** Melanie Trobe, Martin Vareka, Till Schreiner, Patrick Dobrounig, Carina Doler, Ella B. Holzinger, Andreas Steinegger, Rolf Breinbauer

**Affiliations:** ^1^ Institute of Organic Chemistry Graz University of Technology Stremayrgasse 9 8010 Graz Austria

**Keywords:** Inhibitors, Peptide mimetics, Protein–protein interactions, Suzuki coupling, Triflate

## Abstract

Teraryl‐based α‐helix mimetics have proven to be useful compounds for the inhibition of protein‐protein interactions (PPI). We have developed a modular and flexible approach for the synthesis of teraryl‐based α‐helix mimetics using a benzene core unit featuring two leaving groups of differentiated reactivity in the Pd‐catalyzed cross‐coupling used for teraryl assembly. In previous publications we have introduced the methodology of 4‐iodophenyltriflates decorated with the side chains of some of the proteinogenic amino acids. We herein report the core fragments corresponding to the previously missing amino acids Arg, Asn, Asp, Met, Trp and Tyr. Therefore, our set now encompasses all relevant amino acid analogues with the exception of His. In order to be compatible with the triflate moiety, some of the nucleophilic side chains had to be provided in a protected form to serve as stable building blocks. Additionally, cross‐coupling procedures for the assembly of teraryls were investigated.

## Graphical abstract

A series of iodophenyltriflate compounds decorated with the side chains of the proteinogenic amino acids Arg, Asn, Asp, Met, Trp and Tyr is reported. These fragments the set of building blocks for the modular assembly of teraryl‐based alpha‐helix mimetics through sequential Pd cross‐coupling.

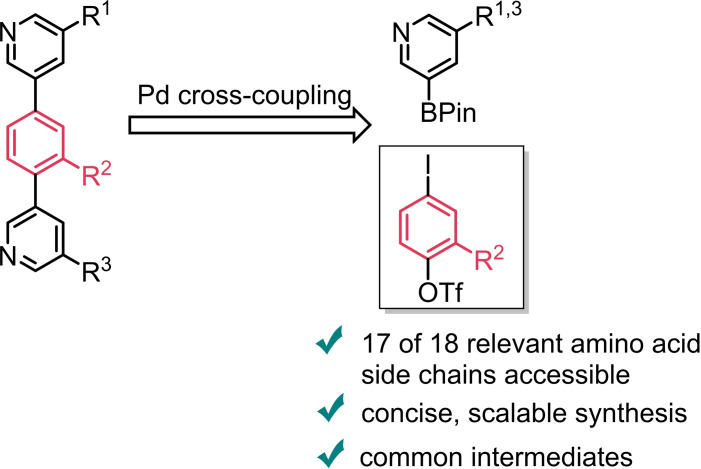

## Introduction

**Figure 1:**
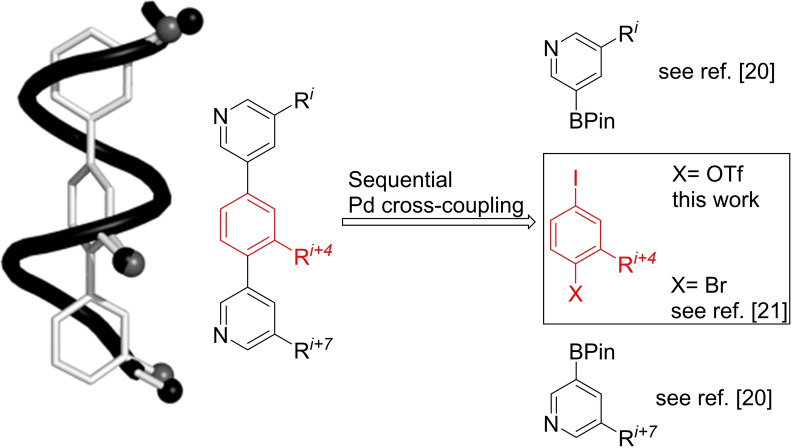
Design principle of teraryl‐based alpha‐helix mimetics (BPin: boronic acid pinacol ester).

Over the last two decades protein‐protein interactions (PPIs) have been recognized as a new concept in drug discovery to control protein function and cure diseases. The number of different PPIs in a human cell is estimated to be beyond 110000, which offers a huge opportunity for Chemical Biology and Medicinal Chemistry but also implies considerable challenges.^[1]^ PPIs are intrinsically difficult to inhibit with small molecule inhibitors as the interfaces of PPIs are rather large with many residues contributing to the binding energy and are therefore difficult to address with low molecular weight compounds.^[2]^ In addition to the stochastic progress of high‐throughput‐screening of compound libraries, a rational approach can be pursued. The structural information available for specific protein complexes is used to identify the so called “hot spots” at the protein interaction area.^[3]^ Various approaches to mimic these peptide fragments, which contribute most to the binding energy in the PPI, are known in the literature. For example, efforts based on conformationally restrained peptides obtained by installation of macrocycles^[4]^ and/or non‐natural amino acids have been reported, such as stapled peptides,^[5]^ β‐peptides^[6]^ or β‐hairpins^[7]^. An alternative strategy focuses on alpha‐helix mimetics as inhibitors of PPIs.[^8^, ^9^] Such mimetics show advantages over the corresponding natural peptide sequences in respect to binding affinity, proteolytic stability, and bioavailability. Hamilton and co‐workers have presented an approach of mimicking α‐helices by suitable positioning of the amino acid side chains at the *i*, *i*+3 (or *i*+4) and *i*+7 residues of a folded α‐helix around a terarylic scaffold.^[10]^ With α‐helices playing a role in ∼60 % of the interaction sites,^[11]^ we have started an effort to produce a comprehensive library of teraryl based α‐helix mimetics. Previously, we have reported about a modular approach for the assembly of such teraryls using electronically differentiated leaving groups.[^12^, ^13^, ^14^, ^15^, ^16^] A central iodophenyltriflate‐core can be decorated by sequential Suzuki‐couplings with preset boronic acid building blocks without intermittent protection or deprotection steps (see Figure 1).^[17]^

In our previous publications, we disclosed the synthesis of iodophenyltriflates with side chains representing the amino acids Ala, Val, Leu, Ile, Phe, “Cys”, “Thr”, “Ser”, Gln, “Glu”, and “Lys”. (Please note that the core fragments marked with “ ” were synthesized in a protected form.) Initial biological data showed promising results for the design principle of teraryl‐based α‐helix mimetics for inhibition of PPI.^[14]^ Therefore, we were even more motivated to extend this approach to provide access to any possible amino acid motif. In this report, we disclose the synthesis of a full set of building blocks featuring all proteinogenic amino acids relevant for α‐helix structures with the exception of His. Our synthetic routes have been optimized to be scalable to provide the building blocks in gram quantities as needed for a library synthesis effort.^[9]^

## Results and Discussion

**Scheme 1:**
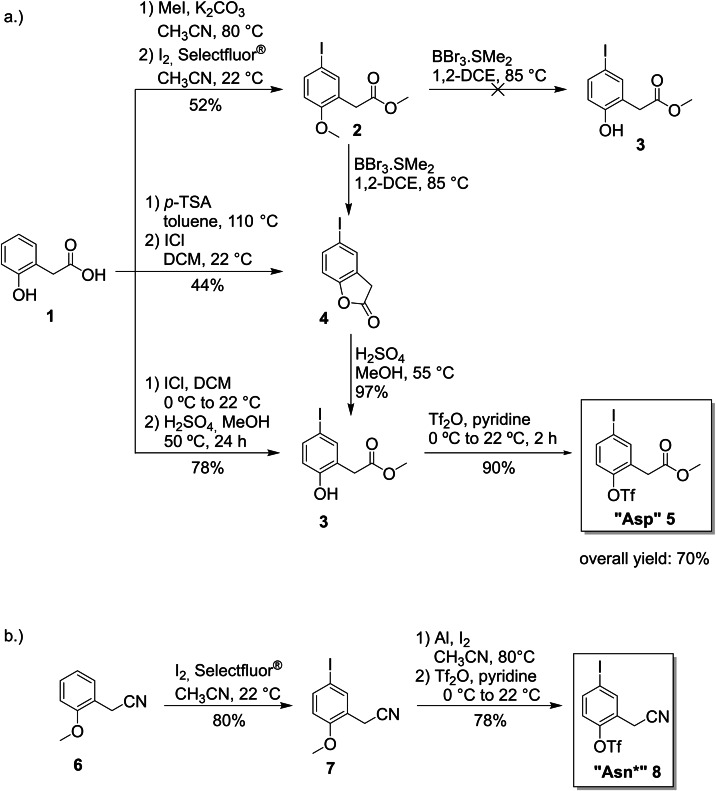
Synthesis of the “Asp” and “Asn*” building blocks (*p*‐TSA: para‐toluenesulfonic acid).

In our strategic reasoning, we aimed to establish short, high yielding and scalable reaction sequences for all core unit fragments – preferably without the use of protecting groups. In addition, the use of general intermediates was desirable. As common motif, ortho‐substituted phenols were used as starting materials throughout most reaction sequences. A wide range of these compounds are commercially available providing easily accessible intermediates for many building blocks with either the correct side chain already installed or a reactive chemical handle for various modifications. With these criteria in mind, we designed reaction sequences for the synthesis of Trp, Met, “Tyr”, “Asn*”, (Please note that core fragments marked with “*” were synthesized in a latent form and had to be converted into the desired functional group after cross coupling.) “Asp” and “Arg” core unit fragments. In the first attempt of synthesizing the “Asp”‐building block **5**, 2‐(2‐hydroxyphenyl)acetic acid (**1**) was used as starting material (Scheme 1a). A methyl ether and a methyl ester were introduced in one step via alkylation with MeI followed by iodination with Selectfluor^®^ and I_2_ ^[18]^ to produce intermediate **2** in 52 % yield. This method was used instead of the standard iodination procedure for building block synthesis (ICl in AcOH)^[12]^ which led to unselective iodination as the methyl ether is a less efficient directing group than the usual phenolic OH. When attempting to cleave the methyl ether in **2** by using BBr_3_.SMe_2_, no product **3** could be detected and instead the corresponding lactone **4** was identified as the main product. To avoid a protection/deprotection strategy, the lactone was iodinated directly in an improved reaction sequence, followed by lactone opening with MeOH to yield the methyl ester **3** in 43 % over three steps. In the last step, the −OTf group was introduced using the already established procedure.^[12]^ Alternatively and more efficiently, **1** can be iodinated directly and subsequently esterified with MeOH shortening the sequence to “Asp” building block **5** to three steps with 70 % overall yield.

To access the Asn core unit fragment **8**, we attempted to convert **5** into the corresponding amide but were not able to find suitable reaction conditions. Aminolysis with NH_3_ in MeOH only led to decomposition of the intermediate. Instead, we used a nitrile as surrogate (Scheme 1b). In contrast to the synthesis of most other building blocks iodination of the corresponding phenol **6** was very unselective and gave a complex mixture of regio‐isomers. In this case, an alternative method was used where the iodine was selectively introduced *para* to a methoxy group by reaction with I_2_/Selectfluor®. For cleavage of the methyl ether, standard conditions with BBr_3_ led to various degrees of dehalogenation depending on the quality of the reagent. This unwanted side reaction could be suppressed by in situ generation of AlI_3_, where two stable solids were used for the preparation of the reactive species as needed.^[19]^ The desired phenol could be produced and after triflation with Tf_2_O in pyridine the masked core unit fragment “Asn*” could be isolated in 3 steps and 63 % overall yield. We were again unsuccessful in converting **8** to the corresponding free amide. Therefore, we suggest that the side chains of either building block **5** or **8** should be converted to the natural Asn side chain after Suzuki‐Miyaura coupling to avoid cleavage of the −I or −OTf functionalities present in the building block by methodology we have presented elsewhere.^[20]^

**Scheme 2:**
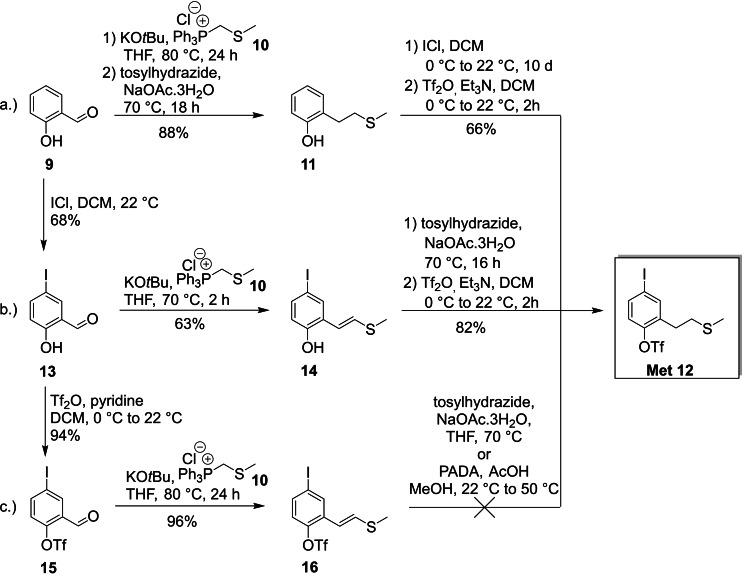
Synthesis of the Met core fragment (KO*t*Bu: potassium *tert*‐butoxide; PADA: dipotassium azodicarboxylate).

The synthesis of the Met building block was attempted starting from aldehyde **15** ^[12]^ via Wittig reaction with potassium *tert*‐butoxide (KO*t*Bu) and phosphonium‐salt **10**, but it was not possible to reduce the double bond in the presence of the −OTf group (Scheme 2c). Therefore, salicylic aldehyde (**9**) and 4‐iodo salicylic aldehyde (**13**) were explored as possible starting materials for the Wittig reaction and subsequent double bond reduction with diimide. When salicylic aldehyde **9** was the starting material, two more reaction steps had to be carried out after reduction (iodination and triflation, Scheme 2a). Both reaction sequences gave the desired Met building block **12** in similar yields. The route starting from **13** was selected since it uses a higher functionalized and common intermediate as precursor (Scheme 2b). For this reaction sequence, a Wittig reaction resulted in thioenolether **14**, which, by a sequence of diimide reduction and triflation, furnished Met core unit fragment **12** in 35 % overall yield.

**Scheme 3:**
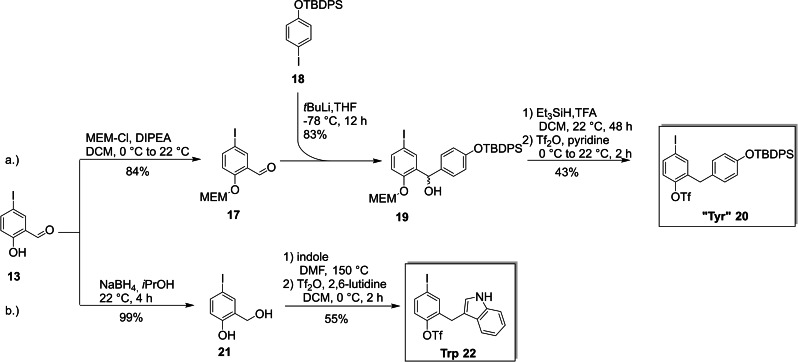
Synthesis of the “Tyr” and Trp building blocks (DIPEA: diisopropylethylamine; MEM: 2‐methoxyethoxymethyl; TFA: trifluoroacetic acid, TBDPS: *tert*‐butyldiphenyl silyl).

4‐Iodo salicylic aldehyde (**13**) conveniently served also as the precursor for synthesizing the “Tyr” and Trp core unit fragment. The “Tyr”‐side chain was introduced by nucleophilic attack of lithiated and *tert*‐butyldiphenylsilyl (TBDPS) protected phenol **18** at (2‐methoxyethoxy)methyl (MEM)‐protected 4‐iodo salicylic aldehyde (**17**) in 83 % yield. In the next step, both the MEM‐protecting group as well as the formed secondary hydroxyl function could be removed with Et_3_SiH/TFA. After triflation under standard conditions, the “Tyr” core unit fragment **20** was isolated in 20 % overall yield over five steps (Scheme 3a). The same reaction sequence was attempted for the synthesis of the His core unit fragment by using various metallated imidazole reagents, but it was not possible to introduce the triflate leaving group at any stage during the synthesis due to decomposition of the imidazole heterocycle. All attempts to add various metallated imidazole reagents to aldehyde **15** (which already bears the triflate group) were also unsuccessful, which indicates that the triflate moiety might be incompatible with the imidazole ring of the His side chain – even when the acidic nitrogen is protected. As a consequence, we have been investigating alternative leaving groups as opposed to the −I/−OTf approach to make a His core fragment building block with electronically differentiated leavings groups available.^[21]^

In order to synthesize the Trp core unit fragment **22**, we soon experienced that the iodine had to be attached before the indole residue was introduced. Otherwise, unselective iodination occurred since the electron‐rich indole ring also provides multiple possible reaction sites for electrophilic aromatic substitution. Therefore, 4‐iodo salicylic aldehyde (**13**) was reduced to the corresponding alcohol **21** with NaBH_4_. The indole ring was then introduced via a Friedel‐Crafts type alkylation^[22]^ and the final product **22** was isolated in 37 % overall yield after triflation using a slightly modified procedure^[23]^ to avoid triflation of the indole moiety (Scheme 3b).

**Scheme 4:**
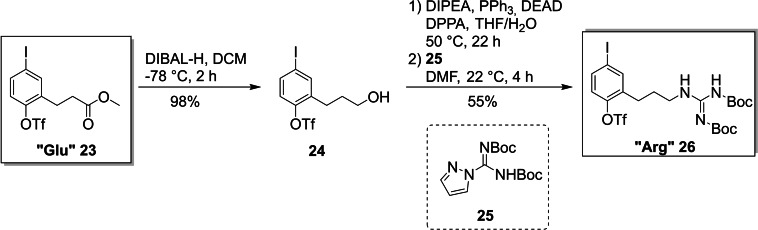
Synthesis of the “Arg” core unit fragment (DEAD: dietyl azodicarboxylate; DPPA: diphenylphosphoryl azide).

The “Glu” core unit fragment **23**, which was synthesized according to our previously developed procedure,^[15]^ found additional use as a convenient precursor for the “Arg” core unit fragment **26**. DIBAL−H reduction of ester **23** delivered alcohol **24**, which was converted to the corresponding amine via a Mitsunobu‐Staudinger sequence. The crude amine was subsequently converted to the Boc‐protected guanidyl residue using guanylating reagent **25**.^[24]^ The desired “Arg” building block **26** was isolated in 54 % yield over three steps starting from the “Glu” building block **23** (Scheme 4).

With the complete set of core building blocks in hand, the assembly of teraryls was investigated. From our previous work^[13]^ we knew that the aqueous solubility of terphenyls can be significantly improved when the top and bottom phenyl rings are replaced by pyridine rings. However, this change in design made the cross‐coupling more challenging, as pyridines can also coordinate to Pd, leading to incomplete conversions in teraryl assembly when the cross‐coupling conditions developed for terphenyls (PdCl_2_(dppf) and CsF in DME) were used.[^12^, ^13^, ^14^] By using the simple Gly‐Ala‐Gly teraryl as a model substrate, we varied precatalyst, base and solvent to find suitable conditions. After considerable experimentation, PdCl_2_(dppf) in MeCN was identified as a suitable catalyst and both coupling steps proceeded nicely at 80 °C. K_2_CO_3_ turned out to be the base of choice for the first coupling step with the reactive iodide leaving group. Alternatively, Ag_2_CO_3_ could be used as an equally effective base. To achieve conversion of the triflate group in the second coupling step, a switch to the more basic Cs_2_CO_3_ was crucial (see SI for details).

**Scheme 5:**
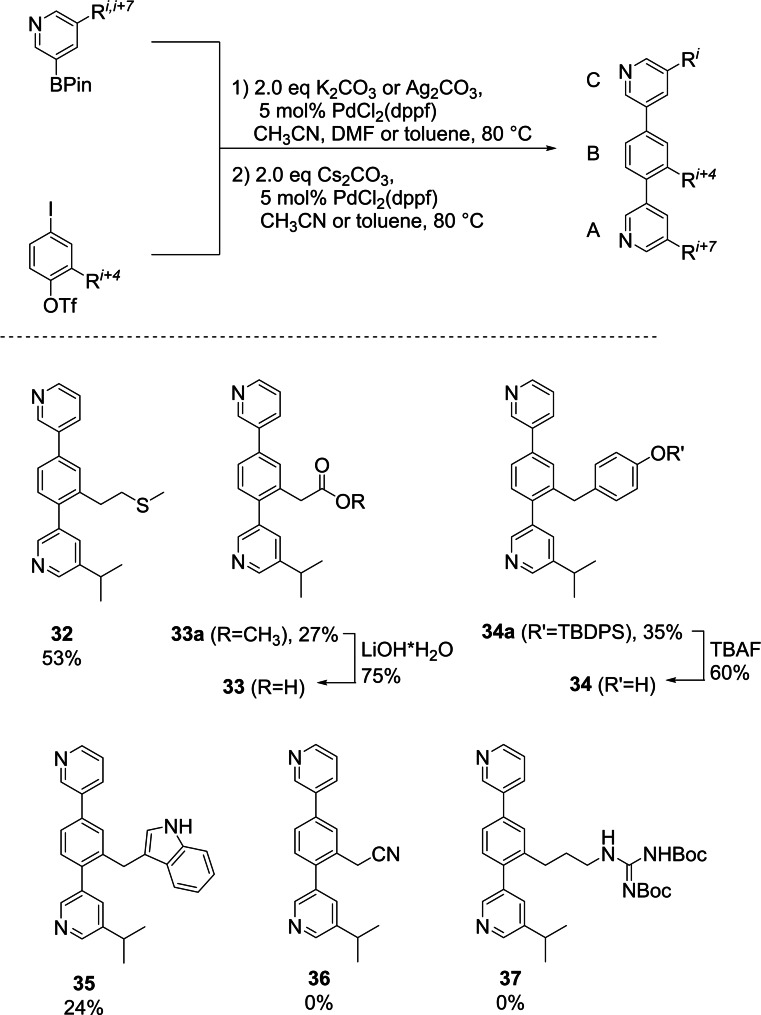
Teraryl assembly with the new core unit fragments (dppf: 1,1’‐bis(diphenylphosphino)ferrocene, TBAF: tetrabutylammonium fluoride).

Unfortunately, we had to notice that further optimization was necessary for coupling of the new building blocks Met (**12**), “Asp” (**5**), “Tyr” (**20**), Trp (**22**), “Asn*” (**8**) and “Arg” (**26**). It was found that especially during the second coupling step, in which the triflate serves as leaving group, rapid hydrolysis to the corresponding phenol may occur. This unwanted side reaction could be suppressed in some cases by performing the reaction in toluene, in which the bases are only sparingly soluble. This way, the Met, “Asp”, “Tyr” and Trp side chains were implemented into teraryl compounds **32**, **33 a**, **34 a** and **35** in moderate to low yields over two coupling steps. The natural aspartate side chain was then introduced via saponification of **33 a**, delivering the Gly‐Asp‐Val mimetic **33** in 75 % yield. The Gly‐Tyr‐Val mimetic **34** could be produced from **34 a** via deprotection of the “Tyr” side chain with tetrabutylammonium fluoride (TBAF) in 60 % yield. Despite extensive screening efforts, it was not possible to avoid hydrolysis of the intermediate diaryl triflates in case of the “Asn*” and “Arg” core building blocks. Therefore, compounds **36** and **37** could not be isolated (Scheme 5).

## Conclusion

**Figure 2:**
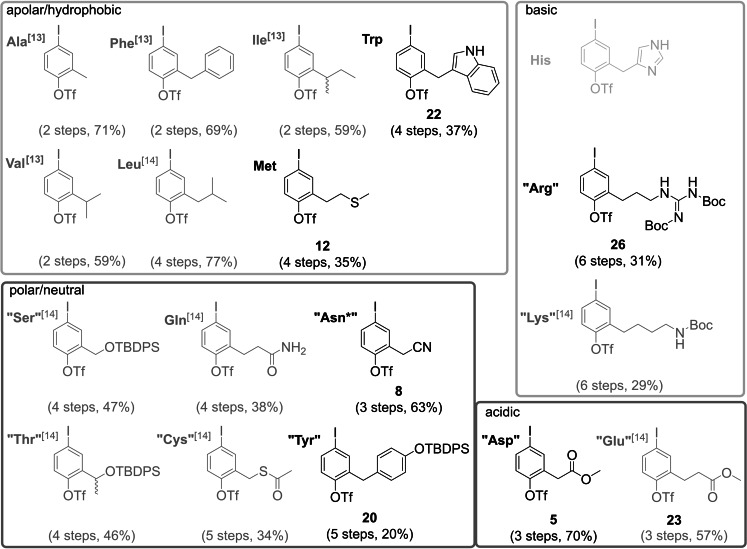
Overview of all core unit fragments (compounds in grey were reported in previous publications).

With this report, we can present a full set of building blocks of iodotriflate core fragments featuring all proteinogenic amino acids – with the exception of His – for the modular synthesis of teraryl‐based α‐helix mimetics (Figure 2). This work represents a big step forward towards our goal that the teraryl‐based approach towards α‐helix mimetics allows a comprehensive coverage of the protein sequence space, which so far has only been accessible to amino acid based designs of PPI inhibitors.^[9]^

As the only exception, the His building block could not be synthesized with an iodotriflate arrangement of leaving groups. Unfortunately, we observed that some of the building blocks with polar side chains such as “Asn*” or “Arg*” suffered from rapid hydrolysis of the labile triflate leaving group during the second cross‐coupling step. Based on our previous experience^[13]^ and the experiments described in this work, we can conclude that the iodotriflate core building blocks are most suited for the assembly of teraryl compounds bearing mainly nonpolar, hydrophobic side chains. Here, the advantage of efficient synthesis of such building blocks from readily available phenols comes into full play. For the assembly of teraryl compounds with more polar side chains, we have explored a modified approach in which a core fragment with two halogen leaving groups (e. g. −Br/−I) is used.^[21]^ Nevertheless, we do not rule out the possibility that with the continuing progress in the development of Pd catalyzed cross‐couplings in the future a more efficient reaction system for the cross coupling of aryltriflates will be available,^[25]^ which would allow to exploit the full synthetic potential of all building blocks presented in this report. Based on the observations described here, such a follow‐up study should focus on identifying an optimized solvent/base system, which must achieve fast and selective coupling whilst also suppressing the undesired hydrolysis of the triflate leaving group.

## Experimental Section

### Representative procedure for the iodination of phenol derivatives

In a one‐neck round‐bottom flask, 1.0 eq iodine monochloride (ICl) was dissolved in DCM (∼1 m) and cooled to 0 °C. 1.0 eq of the corresponding phenol derivative dissolved in DCM (∼1 m) was added. The reaction was warmed to RT and stirred until full conversion was observed. In some cases, additional ICl was added to ensure quantitative conversion. The reaction mixture was diluted with 100 mL DCM and washed with Na_2_S_2_O_3_ solution (25 %, 2×100 mL). The aqueous phase was extracted with DCM (3×50 mL) and the organic layer was then washed with saturated

NaCl solution (1×200 mL). After drying over Na_2_SO_4_ and filtering, the solvent was removed under reduced pressure and the crude product was purified via flash column chromatography or recrystallization.

### Representative procedure for the synthesis of triflate derivatives from the corresponding phenols

In a one‐neck round‐bottom flask, 1.0 eq of the corresponding phenol derivative was dissolved in pyridine (∼1 M). After cooling the solution to 0 °C, 1.1 eq trifluoromethanesulfonic anhydride (Tf_2_O) was carefully added. After stirring 5 min at 0 °C, the solution was allowed to warm to RT and stirred until quantitative conversion was detected by TLC. 60 mL Et_2_O were added and the organic phase was washed with H_2_O (3×30 mL) followed by extraction of the combined aqueous layers with Et_2_O (2×30 mL). The combined organic layers were washed with 1 m HCl (2×60 mL) and saturated NaCl solution (1×60 mL), dried over MgSO_4_, filtered and concentrated in vacuo. The crude product was purified via flash column chromatography.

### Representative procedure for the synthesis of teraryls by consecutive double Suzuki‐Coupling (1^st^ step)

A flame dried Schlenk‐flask was charged with 1.0 eq of the corresponding boronic acid derivative, 2.0 eq K_2_CO_3_ or Ag_2_CO_3_, and 5 mol % PdCl_2_(dppf). After drying in vacuo, a solution of 1.0 eq iodotriflate core building block in absolute, degassed CH_3_CN, DMF or toluene (∼0.2 M) was added. The reaction mixture was stirred at 80 °C until full conversion was detected by GC‐MS or TLC. The typically brown suspension was filtered through a pad of SiO_2_ (3×2 cm, eluted with EtOAc) and the filtrate was concentrated to dryness using a rotary evaporator. The crude product was purified via flash column chromatography or used in the next step without further purification.

### Representative procedure for the synthesis of teraryls by consecutive double Suzuki‐Coupling (2^nd^ step)

Another flame dried Schlenk‐flask was charged with 1.0–1.2 eq of the second boronic acid derivative, 2.0–3.0 eq cesium carbonate (Cs_2_CO_3_), and 5 mol % PdCl_2_(dppf). After drying in vacuo, a solution of the previously prepared intermediate (4‐(pyridin‐3‐yl)phenyl trifluoromethanesulfonate derivative) in absolute, degassed CH_3_CN or toluene (∼0.2 M) was added. The reaction mixture was stirred at 80 °C overnight. The typically black suspension was filtered through a pad of SiO_2_ (3×2 cm, eluent: MeOH) and after concentrating to dryness, the crude product was purified via flash column chromatography.

## Conflict of interest

The authors declare no conflict of interest.

## Supporting information

As a service to our authors and readers, this journal provides supporting information supplied by the authors. Such materials are peer reviewed and may be re‐organized for online delivery, but are not copy‐edited or typeset. Technical support issues arising from supporting information (other than missing files) should be addressed to the authors.

Supporting Information
