# Supplementary material for: A Modular Synthesis of Teraryl‐Based α‐Helix Mimetics, Part 3: Iodophenyltriflate Core Fragments Featuring Side Chains of Proteinogenic Amino Acids
Source: European J Org Chem. 2022 Feb 24;2022(17):e202101278. doi: 10.1002/ejoc.202101278 (PMC9306992; doi:10.1002/ejoc.202101278)

European Journal of Organic Chemistry

Supporting Information

A Modular Synthesis of Teraryl-Based α-Helix Mimetics, Part 3: Iodophenyltriflate Core Fragments Featuring Side Chains of Proteinogenic Amino Acids

Melanie Trobe, Martin Vareka, Till Schreiner, Patrick Dobrounig, Carina Doler, Ella B. Holzinger, Andreas Steinegger, and Rolf Breinbauer*

1 General Experimental Aspects, Materials and Methods

NMR spectra were recorded on a Bruker Avance III 300 MHz FT NMR spectrometer (300.36 MHz (^{1}H), 75.53 MHz (^{13}C)), or on a Varian Unity Inova 500 MHz NB high resolution FT NMR spectrometer (499.76 MHz (^{1}H), 125.67 MHz (^{13}C)) at 27 °C. Chemical shifts δ [ppm] are referenced to residual protonated solvent signals as internal standard [D6]DMSO: δ = 2.50 ppm (^{1}H), 39.52 ppm (^{13}C) and $CDCl_3$: δ = 7.26 ppm (^{1}H), 77.16 ppm (^{13}C).[1] Signal multiplicities are abbreviated as s (singlet), d (doublet), dd (doublet of doublet), t (triplet), dt (doublet of triplet), q (quadruplet), dq (doublet of quadruplet), sept (septet), m (multiplet) with the prefix b in case of broad signals. Superscript abbreviations are used as follows: H^{Ar} (phenyl); abbreviation C_q is used for quaternary carbon atoms. ^{13}C NMR resonances were assigned by APT or 2D-HSQC and -HMBC experiments. GC-MS measurements were performed on an Agilent Technologies 7890A (G3440A) GC system equipped with an Agilent Technologies J&W GC-column HP-5MS ((5%-phenyl)-methylpolysiloxane; length: 30 m; inner-diameter: 0.250 mm; film: 0.25 µm) at a constant helium flow rate (He 5.0; Air Liquide; "Alphagaz"; 1.085 mL/min; average velocity 41.6 cm/sec) in split mode 1/175 (inlet temperature: 250°C; injection volume: 2.0 µL; sample concentration: ~0.5 mg/mL in ethyl acetate (EtOAc), methanol (MeOH), dichloromethane (DCM), or diethyl ether (Et_2O)). The GC was coupled to a 5975C inert mass sensitive detec-tor with triple-axis detector (MSD, EI, 70 eV; transfer line: 300 °C; MS source: 240 °C; MS quad: 180 °C), with a solvent delay of 2.60 min. One general gradient MT_50_S (initial temperature: 50 °C, 1.0 min; linear ramp: 40 °C/min; final temperature: 300 °C; final time: 5.0 min; post run 1.0 min; detecting range: 50.0 to 550.0 amu) was applied. When reactions were monitored by GC-MS, the samples were prepared using a microscale workup. This means, an aliquot was taken from the reaction mixture, quenched by the addition of ~1 mL aqueous solution and ~1 mL DCM, EtOAc, or Et_2O. After proper mixing and phase separation, the organic layer was collected, dried over $MgSO_4$ and filtered through cotton in a Pasteur-pipette. Reaction mixtures containing transition metals were additionally filtered through a short pad of silica gel (~1 cm) over cotton in a Pasteur-pipette (eluted with EtOAc or MeOH). Analytical thin layer chromatography (TLC) was performed on Merck silica gel 60-F254 and spots were visualized by UV-light (λ = 254 and/or 366 nm), and by treatment with cerium ammonium molybdate solution (CAM) (CAM: 2.0 g Ce(IV)SO_4, 50 g $(NH_4)_2MoO_4$, 50 mL concentrated H_2SO_4 in 400 mL water), vanillin solution (15 g vanillin in 250 mL ethanol and 2.5 mL concentrated sulphuric acid), ninhydrin solution (1.5 g ninhydrin in 100 mL n-butanol and 3.0 mL acetic acid) or $FeCl_3$ solution (5 g $FeCl_3$ in 100 mL 0.1M HCl), followed by warming with a

heat gun. Flash column chromatography was performed using silica gel 60 Å (35-70 μ m particle size) from Acros Organics at an air pressure of ~1.5 bar. A 20 to 100-fold excess of silica gel was used with respect to the amount of raw material (exact values are given in experimental procedures). The stationary phase was filled in an appropriately sized column resulting in a pad of 15-25 cm silica gel. The column was equilibrated with the solvent or solvent mixture, and the sample was loaded onto the pad by diluting the crude product with the eluent. If the crude product was not sufficiently soluble in the eluent, the sample was dissolved in a proper solvent (MeOH or EtOAc), and the double amount of silica gel (or Celite®545, particle size 0.02-0.1 mm) was added, followed by removing the solvent using a rotary evaporator and drying in vacuo. The mobile phase was forced through the column by means of a rubber bulb pump. Analytical HPLC analysis was performed on a Shimadzu Nexera Liquid Chromatograph with a tempered column oven. The separation was performed on a C-18-Reversed-Phase column of the type „Poroshell® 120 SB-C18, 3.0 x 100 mm, 2.7 µm“ by Agilent Technologies. For detection, a Shimadzu SPD-M20A Prominence Diode Array Detector at a wavelength of λ = 210 nm and a mass selective detector Shimadzu LCMS-2020 Liquid Chromatograph Mass Spectrometer in ESI positive and ESI negative mode were used. Reversed phase preparative HPLC purifications were performed on a Thermo Scientific UltiMate 3000 system. Detection was accomplished with a Dionex UltiMate Diode Array Detector. The separations were carried out on a Macherey Nagel 125/21 Nucleodur® 100-5 C18EC (125 x 21 mm, 5 µm) column. Acetonitrile and water with 0.05% HCOOH were used as eluents for the purification. The following method was applied: MV_NucleodurC18_001HCOOH_10to100 (0.0 – 13.0 min, linear, 10% CH_3CN to 100% CH_3CN, 13.0 – 15.0 min, isocratic, 100% CH_3CN, flow rate: 12 mL/min, 15.0 – 16.0 min, linear, 100% CH_3CN to 10% CH_3CN, 16 – 18 min, isocratic, 10% CH_3CN, T = 30 °C, flow rate: 12 mL/min). High Resolution Mass Spectrometry (HRMS) was performed on a Waters GCT Premier Micromass (Direct Inlet (DI-EI)). Melting points were determined on a "Mel-Temp" melting-point apparatus (Electrothermal) and are given uncorrected. Chemicals were purchased from Sigma-Aldrich, Fisher Scientific, Merck, or Alfa Aesar. All compounds were used without further purification unless otherwise noted. For determination of concentration of the alkyl-lithium solution in n-hexane a procedure according to KOFRON and BACLAWSKI was used.[1] To 250 mg 2,2-diphenylacetic acid, dissolved in 10 mL dry THF, the alkyl-lithium solution was added dropwise, until a color change from colorless to yellow was detected. The added amount of the alkyl-lithium solution corresponds to the amount of 2,2-diphenylacetic acid. The titre was determined before

every use of the alkyl-lithium solution. DCM was first dried over P_2O_5, distilled, then dried over CaH_2 and distilled under an argon atmosphere before use. THF was dried by heating under reflux under an atmosphere of argon over Na, until benzophenone indicated dryness by a deep blue color and stored over 4Å molecular sieves in an amber glass Schlenk-flask under an argon atmosphere. Molecular sieves were activated by filling a 500 mL round-bottomed flask to one third of its volume with molecular sieves (Sigma-AldricH; beads, 8-12 mesh) and heating the flask in a heating mantle (~150°C) under oil pump vacuum for ~3 days, followed by cooling to room temperature under an atmosphere of argon. When working at a temperature of 0 °C, an ice-water bath served as the cooling agent, and -78 °C was achieved by a dry ice/acetone mixture.

2 General Procedures

2.1 Representative procedure for the iodination of phenol derivatives

In a one-neck round-bottom flask 1.0 eq iodine monochloride (ICl) was dissolved in DCM (~1M) and cooled to 0 °C. 1.0 eq of the corresponding phenol derivative dissolved in DCM (~1M) was added. The reaction was warmed to RT and stirred until full conversion was observed. In some cases, additional ICl was added to ensure quantitative conversion. The reaction mixture was diluted with DCM (100 mL) and washed with $Na_2S_2O_3$ solution (0.1M, 2 x 100 mL). The aqueous phase was extracted with DCM (3 x 50 mL) and the organic layer was then washed with sat. NaCl solution (1 x 200 mL). After drying over Na_2SO_4 and filtering, the solvent was removed under reduced pressure, and the crude product was purified via flash column chromatography or recrystallization.[2]

2.2 Representative procedure for the synthesis of triflate derivatives

In a one-neck round-bottom flask 1.0 eq of the corresponding phenol derivative was dissolved in pyridine (~1M). After cooling the solution to 0 °C, 1.1 eq trifluoromethanesulfonic anhydride (Tf_2O) was carefully added. After stirring 5 min at 0 °C, the solution was allowed to warm to RT and stirred until quantitative conversion was detected via TLC. Et_2O (60 mL) were added, and the organic phase was washed with H_2O (3 x 30 mL), followed by extracting the combined aqueous layers with Et_2O (2 x 30 mL). The combined organic layers were washed with 1M HCl (2 x 60 mL) and sat. NaCl solution (1 x 60 mL), dried over $MgSO_4$, filtered, and concentrated in vacuo. The crude product was purified via flash column chromatography.[3]

2.3 Representative procedure for the synthesis of teraryls by consecutive double Suzuki-Coupling (1st step)

A flame dried Schlenk-flask was charged with 1.0 eq of the corresponding boronic acid derivative, 2.0 eq K_2CO_3 or Ag_2CO_3, and 5 mol% $PdCl_2$(dppf). After drying in vacuo, a solution of 1.0 eq iodotriflate core building block in dry, degassed CH_3CN, DMF or toluene (~0.2M) was added. The reaction mixture was stirred at 80 °C until full conversion was detected via GC-MS or TLC. The typically brown suspension was filtered through a pad of SiO_2 (3 x 2 cm, eluted with MeOH) and the filtrate was concentrated to dryness using a rotary evaporator. The crude product was purified via flash column chromatography or used in the next step without further purification.

2.4 Representative procedure for the synthesis of teraryls by consecutive double Suzuki-Coupling (2nd step)

Another flame dried Schlenk-flask was charged with 1.0-1.2 eq of the second boronic acid derivative, 2.0-3.0 eq cesium carbonate (Cs_2CO_3), and 5 mol% $PdCl_2$(dppf). After drying in vacuo, a solution of the previously prepared intermediate (4-(pyridin-3-yl)phenyl trifluoromethanesulfonate derivative) in dry, degassed CH_3CN or toluene (~0.2M) was added. The reaction mixture was stirred at 80 °C overnight. The typically black suspension was filtered through a pad of SiO_2 (3 x 2 cm, eluent: MeOH) and after concentrating to dryness, the crude product was purified via flash column chromatography.

3 Experimental Procedures and Analytical Data for Building Block Synthesis

3.1 Synthesis of Aspartate building block

3.1.1 Methyl 2-(2-methoxyphenyl)acetate

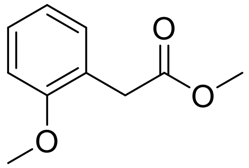

2a

In a 250 mL two-neck round-bottom-flask with reflux condenser 5.00 g 2-hydroxybenzoic acid (**1**) (32.9 mmol, 1.0 eq) and 15.9 g K_2CO_3 (115 mmol, 3.5 eq) were suspended in 50 mL CH_3CN. The pale-yellow suspension was heated to reflux temperature and 5.2 mL MeI (82.2 mmol, 2.5 eq)

dissolved in CH_3CN (15 mL) were added. The reaction was stirred for 24 h. After full conversion was detected via TLC the mixture was cooled to RT and the colorless precipitate was removed by filtration (filter cake rinsed with 3 x 20 mL CH_3CN). The filtrate was concentrated in vacuum and the oily residue was dissolved in DCM (50 mL). A colorless solid precipitated and was again removed by filtration. The filtrate was concentrated under vacuum and the yellow, oily crude was purified via flash column chromatography (500 g SiO_2, 7.0 x 19 cm, eluent: cyclohexane/EtOAc = 20/1, R_f = 0.13, UV and CAM). The pure product **2** was isolated as a pale-yellow oil.
Yield: 4.69 g (79%), pale-yellow oil, $C_{10}H_{12}O_3$ [180.10 g/mol].

^{1}H NMR (300 MHz, $CDCl_3$): δ = 7.29 (t, $^3J_{H,H}$ = 7.8 Hz, 1H; H^{Ar}), 7.24 (d, $^3J_{H,H}$ = 7.3 Hz, 1H; H^{Ar}), 7.18-6.87 (m, 2H; H^{Ar}), 3.82 (s, 3H; CH_3), 3.69 (s, 3H; CH_3), 3.64 (s, 2H; CH_2) ppm; **^{13}C NMR** (76 MHz, $CDCl_3$, APT): δ = 172.4 (C_q; CO), 157.7 (C_q; C^{Ar}), 131.0 (C^{Ar}), 128.7 (C^{Ar}), 123.2 (C_q; C^{Ar}), 120.7 (C^{Ar}), 110.7 (C^{Ar}), 55.6 (CH_3), 52.0 (CH_3), 35.9 (CH_2) ppm; **GC-MS** (EI, 70 eV; MT_50_S): t_R = 5.33 min; **TLC**: R_f = 0.13 (cyclohexane/EtOAc = 20/1, UV and CAM).

Analytical data are in accordance with those reported.[4]

3.1.2 Methyl 2-(5-iodo-2-methoxyphenyl)acetate

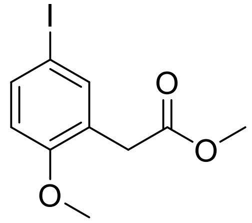

2

A flame dried and argon flushed Schlenk-flask was charged with 2.00 g methyl 2-(2-methoxyphenyl)acetate (**2**) (11.1 mmol, 1.0 eq), 3.93 g Selectfluor® (11.1 mmol, 1.0 eq), and 1.44 g iodine (I_2) (5.66 mmol, 0.5 eq). After dissolving the starting material in CH_3CN (100 mL), the brown solution was stirred for 4 h at RT. After quantitative conversion, CH_3CN was removed under reduced pressure using a rotary evaporator. The oily brown residue was dissolved in DCM (100 mL), washed with H_2O (1 x 100 mL) and the aqueous phase was extracted with DCM (3 x 50 mL). The combined organic layers were washed with $Na_2S_2O_3$ solution (0.1M, 2 x 50 mL) and sat. NaCl solution (1 x 100 mL). After drying over $MgSO_4$ and filtering, the solvent was removed under reduced pressure. Compound **3** was isolated as a colorless solid after flash column

chromatography (200 g SiO_2, 4.5 x 23 cm, eluent: cyclohexane/EtOAc = 20/1, R_f = 0.19, UV and CAM).

Yield: 2.24 g (66%), pale-yellow oil, $C_{10}H_{11}IO_3$ [306.10 g/mol].

^{1}H NMR (300 MHz, $CDCl_3$): δ = 7.53 (dd, $^3J_{H,H}$ = 8.6 Hz, $^4J_{H,H}$ = 2.0 Hz, 1H; H^{Ar}), 7.46 (d, $^4J_{H,H}$ = 1.8 Hz, 1H; H^{Ar}), 6.64 (d, $^3J_{H,H}$ = 8.6 Hz, 1H; H^{Ar}), 3.79 (s, 3H; CH_3), 3.69 (s, 3H; CH_3), 3.57 (s, 2H; CH_2) ppm; **^{13}C NMR** (76 MHz, $CDCl_3$, APT): δ = 171.7 (C_q; C^{Ar}), 157.7 (C_q; C^{Ar}), 139.5 (C^{Ar}), 137.5 (C^{Ar}), 125.8 (C_q; C^{Ar}), 112.9 (C^{Ar}), 82.7 (C_q; C^{Ar}), 55.8 (CH_3), 52.2 (CH_3), 35.4 (CH_2) ppm; **GC-MS** (EI, 70 eV; MT_50_S): t_R = 6.59 min; **TLC**: R_f = 0.19 (cyclohexane/EtOAc = 20/1, UV and CAM).

3.1.3 Benzofuran-2(3H)-one

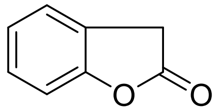

4a

In a 100 mL round-bottom-flask equipped with Dean-Stark apparatus and reflux condenser 4.40 g 2-hydroxybenzoic acid (**1**) (28.9 mmol, 1.0 eq) and 2.75 g p-toluenesulfonic acid monohydrate (14.5 mmol, 0.5 eq) were dissolved in 60 mL toluene. The colorless suspension was heated under reflux for 4 h. The solid was dissolved at reflux temperature and the solution turned brown. Full conversion was detected via TLC. When stirring was stopped two phases separated (brown and yellow). The yellow phase was decanted and cooled to RT from which a colorless solid precipitated. After filtration, the solid was discarded and the filtrate was concentrated in vacuo. The yellow, oily crude was purified via flash column chromatography (250 g SiO_2, 6.0 x 17 cm, eluent: cyclohexane/EtOAc = 15/1, R_f = 0.11, UV and CAM). The pure product **4a** was isolated as a pale-yellow solid.

Yield: 3.11 g (80%), pale-yellow solid, $C_8H_6O_2$ [134.13 g/mol].

^{1}H NMR (300 MHz, $CDCl_3$): δ = 7.21-7.19 (m, 2H; H^{Ar}), 7.07-6.99 (m, 2H; H^{Ar}), 3.64 (s, 2H; CH_2) ppm; **^{13}C NMR** (76 MHz, $CDCl_3$, APT): δ = 174.2 (C_q; CO), 154.9 (C_q; C^{Ar}), 129.1 (C^{Ar}), 124.8 (C^{Ar}), 124.3 (C^{Ar}), 123.2 (C_q; C^{Ar}), 111.0 (C^{Ar}), 33.0 (CH_2) ppm; **GC-MS** (EI, 70 eV; MT_50_S): t_R = 4.79 min; *m/z* (%): 134 (36) [M^+], 106 (21) [M^+-CO], 78 (100) [$M^+-C_2O_2$]; **TLC**: R_f = 0.11 (cyclohexane/EtOAc = 15/1, UV and CAM); **m.p.**$^{exp.}$ = 27-28 °C, (m.p.$^{lit.}$ = 27-28 °C).[5]

Analytical data are in accordance with those reported.[5]

3.1.4 5-Iodobenzofuran-2(3H)-one

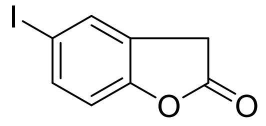

4

The compound was synthesized according to general procedure 2.1 from 3.00 g benzofuran-2(3H)-one (**4**) (22.4 mmol, 1.0 eq) and 3.63 g ICl (22.4 mmol, 1.0 eq) in 45 mL DCM. To achieve quantitative conversion, additional 0.5 eq ICl (total: 3.63 g, 22.4 mmol) were added after 24 h and after 48 h. The orange, solid crude was recrystallized from DCM/cyclohexane (1/4).
Yield: 3.23 g (55%), pale orange powder, $C_8H_5IO_2$ [260.03 g/mol].

^{1}H NMR (300 MHz, $CDCl_3$): δ = 7.64-7.61 (m, 2H; H^{Ar}), 6.89 (d, $^3J_{H,H}$ = 8.1 Hz, 1H; H^{Ar}), 3.73 (s, 2H; CH_2) ppm; **^{13}C NMR** (76 MHz, $CDCl_3$, APT): δ = 173.0 (C_q; CO), 154.5 (C_q; C^{Ar}), 138.0 (C^{Ar}), 133.7 (C^{Ar}), 125.7 (C_q; C^{Ar}), 113.0 (C^{Ar}), 86.8 (C_q; C^{Ar}), 32.7 (CH_2) ppm; **GC-MS** (EI, 70 eV; MT_50_S): t_R = 6.20 min; *m/z* (%): 260 (4) [M^+], 127 (100) [$M^+ - C_8H_5O_2$], 77 (63) [$M^+ - C_2O_2I$]; **m.p.**$^{exp.}$ = 134-137 °C.

Analytical data are in accordance with those reported.[6]

3.1.5 Methyl 2-(2-hydroxy-5-iodophenyl)acetate

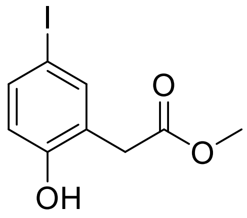

3

In a flame dried and argon flushed Schlenk-flask 1.00 g 5-iodobenzofuran-2(3H)-one (**4**) (3.85 mmol, 1.0 eq) were suspended in 15 mL MeOH. 2.0 mL conc. H_2SO_4 were added to the pale orange suspension and the solid dissolved. The orange solution was heated to 50 °C. After full conversion was detected via TLC, the reaction was cooled to RT, neutralized with sat. $NaHCO_3$ solution and the solvent was removed in vacuum. The oily brown residue was diluted with H_2O (100 mL) and extracted with EtOAc (3 x 100 mL). The combined organic layers were washed with

sat. NaCl solution (1 x 100 mL), dried over $MgSO_4$, filtered and concentrated in vacuum. The crude brown crystals were purified via flash column chromatography (100 g SiO_2, 4.0 x 15 cm, eluent: cyclohexane/EtOAc = 5/1, R_f = 0.20, UV and CAM) and **3** was isolated as light-yellow crystals.
Yield: 882 mg (79%), light yellow crystals, $C_9H_9IO_3$ [292.07 g/mol].

^{1}H NMR (300 MHz, $CDCl_3$): δ = 7.49 (s, 1H; OH), 7.44-7.41 (m, 1H; H^{Ar}), 7.38 (d, $^4J_{H,H}$ = 1.8 Hz, 1H; H^{Ar}), 6.67 (d, $^3J_{H,H}$ = 8.4 Hz, 1H; H^{Ar}), 3.74 (s, 3H; CH_3), 3.60 (s, 2H; CH_2) ppm; **^{13}C NMR** (76 MHz, $CDCl_3$, APT): δ = 174.1 (C_q; CO), 155.4 (C_q; C^{Ar}), 139.5 (C^{Ar}), 138.2 (C^{Ar}), 123.4 (C_q; C^{Ar}), 120.1 (C^{Ar}), 82.7 (C_q; C^{Ar}), 53.1 (CH_3), 37.4 (CH_2) ppm; **GC-MS** (EI, 70 eV; MT_50_S): t_R = 6.52 min; *m/z* (%): 292 (21) [M^+], 260 (100) [M^+-OCH_3], 232 (66) [$M^+-C_2H_3O_2$], 105 (19) [$M^+-C_2H_3O_2I$]; **TLC**: R_f = 0.20 (cyclohexane/EtOAc = 5/1, UV and CAM); **m.p.**$^{exp.}$ = 70-79 °C; **HRMS** (EI): calcd for [M^+]: 291.9597; found: 291.9615.

3.1.6 2-(2-Hydroxy-5-iodophenyl)acetic acid

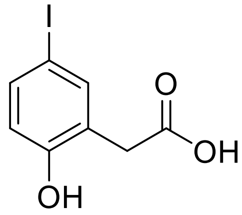

3a

In a 100 mL round-bottom flask 5.34 g ICl (32.9 mmol, 1.0 eq) were dissolved in 40 mL DCM and cooled to 0 °C in an ice bath. 5.00 g 2-Hydroxybenzoic acid (**1**) (32.9 mmol, 1.0 eq) were added in small portions. After a few min a pale-yellow solid started to precipitate. When full conversion (24 h) was detected via HPLC-MS and TLC the reaction mixture was extracted with half-saturated Na_2SO_3 solution (2 x 100 mL) and the precipitate was dissolved into the aqueous phase. The pH-value was then adjusted with 2M $KHSO_4$ solution to pH 1. The product **3a** precipitated as a colorless solid, which was collected by filtration and dried in vacuum.
Yield: 8.86 g (97%), colorless powder, $C_8H_7IO_3$ [278.05 g/mol].

^{1}H NMR (300 MHz, [D_6]DMSO): δ = 12.19 (bs, 1H; COOH), 9.78 (s, 1H; OH), 7.42 (d, $^4J_{H,H}$ = 2.0 Hz, 1H; H^{Ar}), 7.35 (dd, $^3J_{H,H}$ = 8.4 Hz, $^4J_{H,H}$ = 2.1 Hz, 1H; H^{Ar}), 6.63 (d, $^3J_{H,H}$ = 8.4 Hz, 1H; H^{Ar}), 3.44 (s, 2H; CH_2) ppm; **^{13}C NMR** (76 MHz, [D_6]DMSO, APT): δ = 172.3 (C_q; CO), 155.5 (C_q; C^{Ar}), 139.2 (C^{Ar}), 136.2 (C^{Ar}), 125.2 (C_q; C^{Ar}), 117.4 (C^{Ar}), 80.5 (C_q; C^{Ar}), 34.8 (CH_2) ppm;

HPLC-MS (Poroshell, ESI$^-$, MT_general): t_R = 2.84 min; *m/z*: 277 [*M*−H$^+$]; **TLC**: R_f = 0.45 (cyclohexane/EtOAc/AcOH = 100/200/1, UV and Vanillin); **m.p.**$^{exp.}$ = 171-174 °C.

3.1.7 Methyl 2-(2-hydroxy-5-iodophenyl)acetate

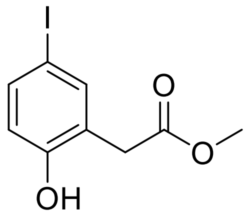

3

In a 250 mL round-bottom flask with air-condenser 5.00 g 2-(2-hydroxy-5-iodophenyl)acetic acid (**3a**) (18.0 mmol, 1.0 eq) were dissolved in 70 mL MeOH and cooled to 0 °C. 10 mL conc. H_2SO_4 were added to the slightly cloudy solution and then the reaction mixture was stirred at 50 °C. After full conversion was detected via TLC, the reaction was cooled to RT, neutralized with sat. $NaHCO_3$ solution (200 mL) and the solvent was removed in vacuum. The residual aqueous phase was extracted with ethyl acetate (4 x 100 mL). The combined organic layers were washed with sat. NaCl solution (1 x 100 mL), dried over Na_2SO_4, filtered and concentrated in vacuum. The crude brown crystals were purified via flash column chromatography (250 g SiO_2, 6.0 x 15 cm, eluent: cyclohexane/EtOAc = 5/1, R_f = 0.20, UV and CAM) and the product **3** was isolated as colorless powder.

Yield: 4.22 g (80%), colorless powder, $C_9H_9IO_3$ [292.07 g/mol].

^{1}H NMR (300 MHz, $CDCl_3$): δ = 7.49 (s, 1H; OH), 7.44-7.41 (m, 1H; H^{Ar}), 7.38 (d, $^4J_{H,H}$ = 1.8 Hz, 1H; H^{Ar}), 6.67 (d, $^3J_{H,H}$ = 8.4 Hz, 1H; H^{Ar}), 3.74 (s, 3H; CH_3), 3.60 (s, 2H; CH_2) ppm; **^{13}C NMR** (76 MHz, $CDCl_3$, APT): δ = 174.1 (C_q; CO), 155.4 (C_q; C^{Ar}), 139.5 (C^{Ar}), 138.2 (C^{Ar}), 123.4 (C_q; C^{Ar}), 120.1 (C^{Ar}), 82.7 (C_q; C^{Ar}), 53.1 (CH_3), 37.4 (CH_2) ppm; **GC-MS** (EI, 70 eV; MT_50_S): t_R = 6.52 min; *m/z* (%): 292 (21) [*M*$^+$], 260 (100) [*M*$^+$−OCH_3], 232 (66) [*M*$^+$−$C_2H_3O_2$], 105 (19) [*M*$^+$−$C_2H_3O_2I$]; **TLC**: R_f = 0.20 (cyclohexane/EtOAc = 5/1, UV and CAM); **m.p.**$^{exp.}$ = 76-79 °C; **HRMS** (EI): calcd for [*M*$^+$]: 291.9597; found: 291.9615.

3.1.8 Methyl 2-(5-iodo-2-(((trifluoromethyl)sulfonyl)oxy)phenyl)acetate

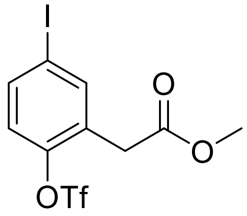

5

Compound **5** was prepared according to general procedure 2.2 from 500 mg phenol derivative **3** (1.71 mmol, 1.0 eq) and 531 mg Tf_2O (1.88 mmol, 1.1 eq) in 1.5 mL pyridine. Quantitative conversion was detected after 2 h. After purification via flash column chromatography (75 g SiO_2, 3.0 x 20 cm, eluent: cyclohexane/EtOAc = 20/1, R_f = 0.25, UV and CAM) compound **5** was isolated as a colorless oil.
Yield: 655 mg (90%), colourless oil, $C_{10}H_8F_3IO_5S$ [424.13 g/mol].

^{1}H NMR (300 MHz, $CDCl_3$): δ = 7.72 (d, $^4J_{H,H}$ = 2.1 Hz, 1H; H^{Ar}), 7.67 (dd, $^3J_{H,H}$ = 8.7 Hz, $^4J_{H,H}$ = 2.1 Hz, 1H; H^{Ar}), 7.04 (d, $^3J_{H,H}$ = 8.4 Hz, 1H; H^{Ar}), 3.71 (s, 3H; CH_3), 3.67 (s, 2H; CH_2) ppm; **^{13}C NMR** (76 MHz, $CDCl_3$, APT): δ = 169.8 (CO), 148.1 (C_q; C^{Ar}), 141.4 (C^{Ar}), 138.6 (C^{Ar}), 128.6 (C_q; C^{Ar}), 123.4 (C^{Ar}), 118.7 (q, $^1J_{C,F}$ = 318 Hz; CF_3), 93.3 (C_q; C^{Ar}), 52.7 (CH_3), 35.3 (CH_2) ppm; **GC-MS** (EI, 70 eV; MT_50_S): t_R = 6.36 min; *m/z* (%): 424 (60) [M^+], 365 (31) [$M^+-C_2H_3O_2$], 275 (100) [$M^+-CF_3O_3S$], 164 (60) [$M^+-CF_3IO_2S$]; **TLC**: R_f = 0.25 (cyclohexane/EtOAc = 20/1, UV and CAM); **HRMS** (EI): calcd for [M^+]: 423.9089; found: 423.9132.

3.2 Synthesis of Asparagine building block

3.2.1 2-(5-Iodo-2-methoxyphenyl)acetonitrile

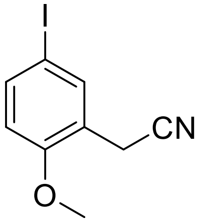

7

A flame dried and argon flushed Schlenk-flask was charged with 1.00 g 2-(2-methoxyphenyl)-acetonitrile (**6**) (6.79 mmol, 1.0 eq), 2.41 g Selectfluor® (6.79 mmol, 1.0 eq), and 880 mg iodine

(I_2) (3.47 mmol, 0.5 eq). After dissolving the starting material in 65 mL CH_3CN, the brown solution was stirred for 4 h at RT. After quantitative conversion was detected, CH_3CN was removed under reduced pressure using a rotary evaporator. The oily brown residue was dissolved in DCM (100 mL), washed with H_2O (1 x 100 mL) and the aqueous phase was extracted with DCM (3 x 50 mL). The combined organic layers were washed with $Na_2S_2O_3$ solution (25%, 2 x 50 mL) and sat. NaCl solution (1 x 100 mL). After drying over $MgSO_4$ and filtering, the solvent was removed under reduced pressure. Compound **7** was isolated as a colorless solid after flash column chromatography (100 g SiO_2, 4.0 x 15 cm, eluent: cyclohexane/EtOAc = 6/1, R_f = 0.28, UV and CAM).
Yield: 1.49 g (80%), colourless crystals, C_9H_8INO [273.07 g/mol].

^{1}H NMR (300 MHz, $CDCl_3$): δ = 7.63-7.58 (m, 2H; H^{Ar}), 6.66 (d, $^3J_{H,H}$ = 8.5 Hz, 1H; H^{Ar}), 3.84 (s, 3H; CH_3), 3.63 (s, 2H; CH_2) ppm; **^{13}C NMR** (76 MHz, $CDCl_3$, APT): δ = 156.8 (C_q; C^{Ar}), 138.5 (C^{Ar}), 137.7 (C^{Ar}), 121.3 (C_q; C^{Ar}), 117.5 (C_q; CN), 112.8 (C^{Ar}), 82.6 (C_q; C^{Ar}), 55.8 (CH_3), 18.4 (CH_2) ppm; **GC-MS** (EI, 70 eV; MT_50_S): t_R = 6.58 min; *m/z* (%): 273 (100) [M^+], 258 (31) [M^+-CH_3], 233 (11) [$M^+-C_2H_2N$], 146 (9) [M^+-I]; **TLC**: R_f = 0.28 (cyclohexane/EtOAc = 6/1, UV and CAM); **m.p.**$^{exp.}$ = 67-70 °C; **HRMS** (EI): calcd for [M^+]: 272.9651; found: 272.9658.

3.2.2 2-(2-Hydroxy-5-iodophenyl)acetonitrile

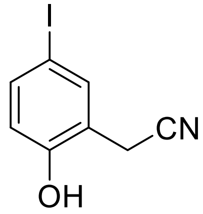

8a

In a flame dried and argon flushed Schlenk-flask 261 mg Al-powder (9.67 mmol, 1.1 eq) were suspended in 40 mL dry CH_3CN. 3.68 g I_2 (14.5 mmol, 1.7 eq) were added and the red-brown suspension was stirred at 80 °C for 2 h. The formation of AlI_3 was indicated by decoloring of the suspension. 2.40 g **7** (8.79 mmol, 1.0 eq) were added and the reaction mixture was stirred for 3 d at 80 °C. When full conversion was detected via TLC, the reaction was diluted with EtOAc (100 mL) and quenched by the addition of 1M HCl (100 mL). The phases were separated, and the aqueous phase was extracted with EtOAc (2 x 100 mL). The combined organic layers were washed with 0.1M $Na_2S_2O_3$ solution (1 x 100 mL) and sat. NaCl solution (1 x 100 mL), dried over $MgSO_4$ and filtered. After purification via flash column chromatography (75 g SiO_2, 4.0 x 15 cm, eluent:

cyclohexane/EtOAc = 4/1, R_f = 0.22, UV and CAM) compound **8a** was isolated as a pale-yellow powder.

Yield: 2.10 g (92%), pale-yellow powder, C_8H_6INO [259.04 g/mol].

^{1}H NMR (300 MHz, $CDCl_3$): δ = 7.62 (d, $^4J_{H,H}$ = 1.4 Hz, 1H; H^{Ar}), 7.48 (dd, $^3J_{H,H}$ = 8.4 Hz, $^4J_{H,H}$ = 1.8 Hz, 1H; H^{Ar}), 6.59 (d, $^3J_{H,H}$ = 8.4 Hz, 1H; H^{Ar}), 5.75 (bs, 1H; OH), 3.67 (s, 2H; CH_2) ppm; **^{13}C NMR** (76 MHz, $CDCl_3$, APT): δ = 153.3 (C_q; C^{Ar}), 138.5 (C^{Ar}), 138.2 (C^{Ar}), 119.7 (C_q; C^{Ar}), 117.6 (C^{Ar}), 117.5 (C_q; CN), 82.7 (C_q; C^{Ar}), 18.3 (CH_2) ppm; **GC-MS** (EI, 70 eV; MT_50_S): t_R = 6.22 min; *m/z* (%): 259 (100) [M^+], 232 (56) [$M^+-C_2H_2N$], 204 (24) [$M^+-C_3H_3O$]; **TLC**: R_f = 0.22 (cyclohexane/EtOAc = 4/1, UV and CAM); **m.p.**$^{exp.}$ = 121 °C (decomposition); **HRMS** (EI): calcd for [M^+]: 258.9494; found: 258.9490.

3.2.3 2-(Cyanomethyl)-4-iodophenyl trifluoromethanesulfonate

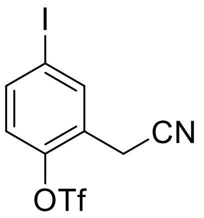

8

Compound **8** was prepared according to general procedure 2.2 from 432 mg phenol derivative **8a** (1.67 mmol, 1.0 eq) and 518 mg Tf_2O (1.83 mmol, 1.1 eq) in 4 mL pyridine. Quantitative conversion was detected after 2 h. After purification via flash column chromatography (60 g SiO_2, 3.0 x 19 cm, eluent: cyclohexane/Et_2O = 10/1, R_f = 0.26, UV and CAM) compound **8** was isolated as a brown oil.

Yield: 556 mg (85%), brown oil, $C_9H_5F_3INO_3S$ [391.11 g/mol].

^{1}H NMR (300 MHz, $CDCl_3$): δ = 7.96 (s, 1H; H^{Ar}), 7.79 (dd, $^3J_{H,H}$ = 8.6 Hz, $^4J_{H,H}$ = 1.9 Hz, 1H; H^{Ar}), 7.10 (d, $^3J_{H,H}$ = 8.7 Hz, 1H; H^{Ar}), 3.81 (s, 2H; CH_2) ppm; **^{13}C NMR** (76 MHz, $CDCl_3$, APT): δ = 146.7 (C_q; C^{Ar}), 139.7 (C^{Ar}), 139.5 (C^{Ar}), 125.6 (C_q; C^{Ar}), 123.8 (C^{Ar}), 118.6 (q, $^1J_{C,F}$ = 318 Hz; CF_3), 115.5 (C_q; CN), 93.9 (C_q; C^{Ar}), 18.8 (CH_2) ppm; **GC-MS** (EI, 70 eV; MT_50_S): t_R = 6.36 min; *m/z* (%): 391 (51) [M^+], 258 (100) [$M^+-CF_3O_2S$], 131 (13) [$M^+-CF_3IO_2S$]; **TLC**: R_f = 0.26 (cyclohexane/EtOAc = 10/1, UV and CAM); **HRMS** (EI): calcd for [M^+]: 390.8987; found: 390.8986.

3.3 Synthesis of Methionine building block

3.3.1.1.1 ((Methylthio)methyl)triphenylphosphonium chloride

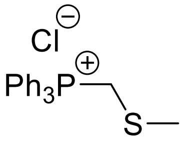

10

In a flame dried and argon flushed Schlenk-flask 20 g PPh_3 (76.3 mmol, 1.0 eq) were dissolved in 40 mL dry toluene. 6.4 mL Chloromethyl methyl sulfide (76.3 mmol, 1.0 eq) were added upon which the solution turned yellow. The reaction mixture was stirred at 100 °C and within 24 h a colorless precipitate was formed. The suspension was cooled to 0 °C and the precipitate was collected by filtration and washed with toluene (3 x 5 mL).
Yield: 18.7 g (68%), colorless powder, $C_{20}H_{20}ClPS$ [358.86 g/mol].

^{1}H NMR (300 MHz, [D_6]DMSO): δ = 7.92-7.75 (m, 15H; H^{Ar}), 5.11 (d, $^2J_{H,P}$ = 9.0 Hz, 2H; CH_2), 1.90 (s, 3H; CH_3) ppm; **^{13}C NMR** (76 MHz, [D_6]DMSO, APT): δ = 135.1 (d, $^4J_{C,P}$ = 3 Hz; C^{Ar}), 134.0 (d, $^3J_{C,P}$ = 10 Hz; C^{Ar}), 130.1 (d, $^2J_{C,P}$ = 12 Hz; C^{Ar}), 118.2 (d, $^1J_{C,P}$ = 87 Hz; C_q; C^{Ar}), 23.8 (d, $^1J_{C,P}$ = 51 Hz; CH_2), 17.2 (d, $^3J_{C,P}$ = 3 Hz; CH_3) ppm; **m.p.**$^{exp.}$ = 225-227 °C (m.p.$^{lit.}$ = 224-226 °C).[7]

Analytical data are in accordance with those reported.[8]

3.3.2 2-(2-(Methylthio)vinyl)phenol

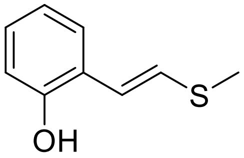

11a

In a flame dried and argon flushed 250 mL three-neck round-bottom flask equipped with reflux condenser and an argon-inlet 4.41 g phosphonium-salt **10** (12.3 mmol, 1.5 eq) were suspended in 40 mL dry THF. After cooling the suspension to 0 °C, 1.38 g KO*t*Bu (12.3 mmol, 1.5 eq) were added. The yellow reaction mixture was stirred at 50 °C for 60 min. The suspension was cooled to 0 °C, 870 µL salicylaldehyde (**9**) (8.19 mmol, 1.0 eq) were added and the orange suspension was heated to 80 °C. After quantitative conversion (20 h) the reaction mixture was cooled to RT and

quenched by the addition of 40 mL sat. NH_4Cl solution. The phases were separated and the aqueous phase was diluted with 50 mL H_2O. The aqueous phase was extracted with DCM (3 x 50 mL) and the combined organic layers were washed with sat. NaCl solution (1 x 100 mL), dried over Na_2SO_4 and filtered. The solvent was removed under reduced pressure and the yellow, oily crude product was purified via flash column chromatography (250 g SiO_2, 5.5 x 16 cm, eluent: cyclohexane/EtOAc = 10/1, R_f = 0.11, UV and CAM).

Yield: 1.29 g (95%), pale-yellow oil, $C_9H_{10}OS$ [166.24 g/mol].

^{1}H NMR (300 MHz, $CDCl_3$): δ = 7.69 (d, $^3J_{H,H}$ = 7.6 Hz, 1H; CH), 7.48 (dt, $^3J_{H,H}$ = 7.5 Hz, $^4J_{H,H}$ = 1.4 Hz, 1H; H^{Ar}), 7.32-7-27 (m, 2H; H^{Ar}), 7.16 (d, $^3J_{H,H}$ = 8.0 Hz, 1H; H^{Ar}), 6.91 (d, $^3J_{H,H}$ = 15.5 Hz, 1H; CH), 2.79 (s, 3H; CH_3) ppm; **^{13}C NMR** (76 MHz, $CDCl_3$, APT): δ = 152.1 (C_q; C^{Ar}), 127.9 (C^{Ar}), 127.8 (CH), 127.1 (C^{Ar}), 124.8 (C_q; C^{Ar}), 121.3 (C^{Ar}), 119.4 (CH), 116.0 (C^{Ar}), 15.0 (CH_3) ppm; **GC-MS** (EI, 70 eV; MT_50_S): t_R = 6.23 min; *m/z* (%): 166 (60) [M^+], 151 (6) [M^+−CH_3], 119 (66) [M^+−CH_3S]; **TLC**: R_f = 0.11 (cyclohexane/EtOAc = 10/1, UV and CAM).

Analytical data are in accordance with those reported.[9]

3.3.3 2-(2-(Methylthio)ethyl)phenol

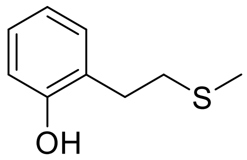

11

An argon flushed 250 mL two-neck round-bottom flask, equipped with argon-inlet and reflux-condenser, was charged with 2.45 g phenol derivative **11a** (15.0 mmol, 1.0 eq) dissolved in 150 mL THF. 16.8 g p-Tosylhydrazide (90.2 mmol, 6.0 eq) and 12.3 g NaOAc.3H_2O (90.2 mmol, 6.0 eq) were added and the yellow suspension was heated to 70 °C. The reaction mixture was cooled to RT after quantitative conversion (18 h) was detected via GC-MS. Water (100 mL) was added and the phases were separated. The aqueous layer was extracted with EtOAc (3 x 100 mL) and the combined organic layers were dried over Na_2SO_4. The Na_2SO_4 was removed by filtration and the solvent was removed under reduced pressure. The crude product was purified via flash column chromatography (250 g SiO_2, 6.0 x 17 cm, eluent: cyclohexane/EtOAc = 10/1, R_f = 0.17, UV and CAM).

Yield: 2.53 g (93%), pale-yellow oil, $C_9H_{12}OS$ [168.25 g/mol].

^{1}**H NMR** (300 MHz, $CDCl_3$): δ = 7.13-7.08 (m, 2H; H^{Ar}), 6.87 (t, $^{3}J_{H,H}$ = 7.4 Hz, 1H; H^{Ar}), 6.79 (d, $^{3}J_{H,H}$ = 7.7 Hz, 1H; H^{Ar}), 5.48 (bs, 1H; OH), 2.93 (t, $^{3}J_{H,H}$ = 7.1 Hz, 2H; CH_2), 2.77 (t, $^{3}J_{H,H}$ = 7.2 Hz, 2H; CH_2), 2.13 (s, 3H; CH_3) ppm; ^{13}**C NMR** (76 MHz, $CDCl_3$, APT): δ = 154.0 (C_q; C^{Ar}), 130.9 (C^{Ar}), 128.0 (C^{Ar}), 127.4 (C_q; C^{Ar}), 121.2 (C^{Ar}), 116.3 (C^{Ar}), 35.0 (CH_2), 31.1 (CH_2), 16.0 (CH_3) ppm; **GC-MS** (EI, 70 eV; MT_50_S): t_R = 5.89 min; *m/z* (%): 168 (50) [M^+], 120 (47) [M^+−CH_3S], 107 (100) [M^+−C_2H_5S]; **TLC**: R_f = 0.17 (cyclohexane/EtOAc = 10/1, UV and CAM); **HRMS** (EI): calcd for [M^+]: 168.0609; found: 168.0607.

3.3.4 4-Iodo-2-(2-(methylthio)ethyl)phenol

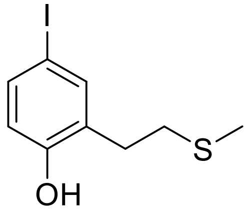

12a

Compound **12a** was prepared according to general procedure 2.1 from 1.93 g ICl (11.9 mmol, 1.0 eq) in 12 mL DCM and 2.00 g **11** (11.9 mmol, 1.0 eq) in 3.5 mL DCM. After 5 d additional 1.55 g ICl (9.52 mmol, 0.8 eq) were added and quantitative conversion was detected after 10 d. After purification via flash column chromatography (125 g SiO_2, 5.0 x 12 cm, eluent: cyclohexane/EtOAc = 10/1, R_f = 0.24, UV and CAM) compound **12a** was isolated as a pale brown oil.

Yield: 2.54 g (73%), pale brown oil, $C_9H_{11}IOS$ [294.15 g/mol].

^{1}**H NMR** (300 MHz, $CDCl_3$): δ = 7.40-7.35 (m, 2H; H^{Ar}), 6.57 (d, $^{3}J_{H,H}$ = 8.3 Hz, 1H; H^{Ar}), 5.73 (s, 1H; H^{Ar}), 2.86 (t, $^{3}J_{H,H}$ = 6.8 Hz, 2H; CH_2), 2.73 (t, $^{3}J_{H,H}$ = 6.7 Hz, 2H; CH_2), 2.12 (s, 3H; CH_3) ppm; ^{13}**C NMR** (76 MHz, $CDCl_3$): δ = 154.1 (C_q; C^{Ar}), 139.4 (C^{Ar}), 136.8 (C^{Ar}), 130.4 (C_q; C^{Ar}), 118.7 (C^{Ar}), 83.2 (C_q; C^{Ar}), 34.8 (CH_2), 30.9 (CH_2), 16.1 (CH_3) ppm; **GC-MS** (EI, 70 eV; MT_50_S): t_R = 7.14 min; *m/z* (%): 294 (56) [M^+], 246 (100) [M^+−CH_3S], 233 (49) [M^+−C_2H_5S]; **TLC**: R_f = 0.24 (cyclohexane/EtOAc = 10/1, UV and CAM); **HRMS** (EI): calcd for [M^+]: 293.9576; found: 293.9582.[9]

3.3.5 2-Hydroxy-5-iodobenzaldehyde

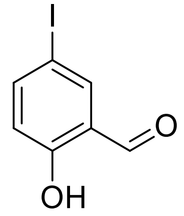

13

The compound was synthesized according to general procedure 2.1 from 16.1 g iodine monochloride (ICl) (99.1 mmol, 1.0 eq) dissolved in 100 mL DCM and 10.0 mL salicylaldehyde (**9**) (99.1 mmol, 1.0 eq) dissolved in 37 mL DCM. The crude product was recrystallized from cyclohexane.

Yield: 16.7 g (68%); pale-yellow powder, $C_7H_5IO_2$ [248.02 g/mol].

^{1}H NMR (300 MHz, $CDCl_3$): δ = 10.94 (s, 1H; CHO), 9.83 (s, 1H; OH), 7.84 (d, $^4J_{H,H}$ = 2.2 Hz, 1H; H^{Ar}), 7.76 (dd, $^3J_{H,H}$ = 8.8 Hz, $^4J_{H,H}$ = 2.2 Hz, 1H; H^{Ar}), 6.80 (d, $^3J_{H,H}$ = 8.8 Hz, 1H; H^{Ar}) ppm; **^{13}C NMR** (76 MHz, $CDCl_3$, APT): δ = 195.5 (CHO), 161.3 (C_q; C^{Ar}), 145.4 (C^{Ar}), 142.0 (C^{Ar}), 122.7 (C_q; C^{Ar}), 120.3 (C^{Ar}), 80.5 (C_q; C^{Ar}) ppm; **GC-MS** (EI, 70 eV; MT_50_S): t_R = 5.48 min; *m/z* (%): 248 (100) [M^+], 219 (6) [M^+−CHO], 202 (3) [M^+−CH_2O_2]; **TLC**: R_f = 0.30 (cyclohexane/EtOAc = 25/1, UV and CAM); **m.p.**$^{exp.}$ = 97-99 °C (m.p.$^{lit.}$ = 97-98 °C).[2]

Analytical data are in accordance with those reported.[2]

3.3.6 4-Iodo-2-(2-(methylthio)vinyl)phenol

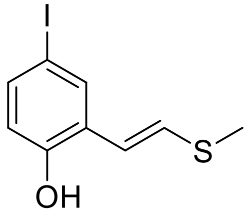

14

In a flame dried and argon flushed Schlenk-flask 1.30 g phosphonium-salt **10** (3.63 mmol, 1.5 eq) were suspended in 12 mL dry THF and 407 mg KO*t*Bu (3.63 mmol, 1.5 eq) were added. The yellow reaction mixture was stirred at 50 °C for 60 min. The suspension was cooled to 0 °C and 600 mg **13** (2.42 mmol, 1.0 eq) were added and the orange suspension was heated to 70 °C. After quantitative conversion (2 h) the reaction mixture was cooled to RT and quenched by the addition

of sat. NH_4Cl solution (60 mL). The phases were separated and the aqueous phase was diluted with H_2O (50 mL). The aqueous phase was extracted with DCM (3 x 50 mL) and the combined organic layers were washed with sat. NaCl solution (1 x 100 mL), dried over Na_2SO_4 and filtered. The solvent was removed under reduced pressure and the yellow, oily crude product was purified via flash column chromatography (150 g SiO_2, 4.0 x 25 cm, eluent: cyclohexane/EtOAc = 10/1, R_f = 0.11, UV and CAM).

Yield: 444 mg (63%), pale-yellow powder, C_9H_9IOS [292.13 g/mol].

^{1}H NMR (300 MHz, $CDCl_3$): δ = 7.55 (d, $^4J_{H,H}$ = 1.8 Hz, 1H; H^{Ar}), 7.31 (dd, $^3J_{H,H}$ = 8.4 Hz, $^4J_{H,H}$ = 2.0 Hz, 1H; H^{Ar}), 6.85 (d, $^3J_{H,H}$ = 15.5 Hz, 1H; CH), 6.52 (d, $^3J_{H,H}$ = 8.4 Hz, 1H; H^{Ar}), 6.33 (d, $^3J_{H,H}$ = 15.5 Hz, 1H; CH), 4.95 (s, 1H; OH), 2.37 (s, 3H; CH_3) ppm; **^{13}C NMR** (76 MHz, $CDCl_3$, APT): δ = 151.9 (C_q; C^{Ar}), 136.3 (C^{Ar}), 135.6 (C^{Ar}), 129.6 (CH), 127.5 (C_q; C^{Ar}), 118.2 (CH), 117.5 (C^{Ar}), 83.5 (C_q; C^{Ar}), 15.0 (CH_3) ppm; **GC-MS** (EI, 70 eV; MT_50_S): t_R = 7.39 min; *m/z* (%): 292 (50) [M^+], 245 (33) [M^+−CH_3S], 118 (100) [M^+−CH_3IS]. **TLC**: R_f = 0.11 (cyclohexane/EtOAc = 10/1, UV and CAM); **m.p.**$^{exp.}$ = 73-76 °C; **HRMS** (EI): calcd for [M^+]: 291.9419; found: 292.9415.

3.3.7 4-Iodo-2-(2-(methylthio)ethyl)phenol

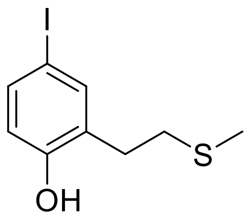

12a

A 25 mL round-bottom flask equipped with reflux-condenser was charged with 350 mg **14** (1.20 mmol, 1.0 eq) and 12 mL THF. 1.34 g p-tosylhydrazide (7.19 mmol, 6.0 eq) and 580 mg $NaOAc.3H_2O$ (7.19 mmol, 6.0 eq) were added and the pale-yellow suspension was stirred at 70 °C until quantitative conversion was detected via GC-MS (16 h). The reaction mixture was cooled to RT and water (50 mL) was added. The phases were separated, and the aqueous layer was extracted with EtOAc (3 x 50 mL). The combined organic layers were dried over Na_2SO_4, filtered and the solvent was removed under reduced pressure. The crude product was purified via flash column chromatography (30 g SiO_2, 2.5 x 17 cm, eluent: cyclohexane/EtOAc = 10/1, R_f = 0.17, UV and CAM).

Yield: 322 mg (91%), pale-yellow powder, $C_9H_{11}IOS$ [294.15 g/mol].

^{1}H NMR (300 MHz, $CDCl_3$): δ = 7.40-7.35 (m, 2H; H^{Ar}), 6.57 (d, $^3J_{H,H}$ = 8.3 Hz, 1H; H^{Ar}), 5.73 (s, 1H; OH), 2.86 (t, $^3J_{H,H}$ = 6.8 Hz, 2H; CH_2), 2.73 (t, $^3J_{H,H}$ = 6.7 Hz, 2H; CH_2), 2.12 (s, 3H; CH_3) ppm; **^{13}C NMR** (76 MHz, $CDCl_3$, APT): δ = 154.1 (C_q; C^{Ar}), 139.4 (C^{Ar}), 136.8 (C^{Ar}), 130.4 (C_q; C^{Ar}), 118.7 (C^{Ar}), 83.2 (C_q; C^{Ar}), 34.8 (CH_2), 30.9 (CH_2), 16.1 (CH_3) ppm; **GC-MS** (EI, 70 eV; MT_50_S): t_R = 7.14 min; *m/z* (%): 294 (56) [M^+], 246 (100) [M^+−CH_3S], 233 (49) [M^+−C_2H_5S]; **TLC**: R_f = 0.17 (cyclohexane/EtOAc = 10/1, UV and CAM); **m.p.**$^{exp.}$ = 52-54 °C; **HRMS** (EI): calcd for [M^+]: 293.9576; found: 293.9582.

3.3.8 4-Iodo-2-(2-(methylthio)ethyl)phenyl trifluoromethanesulfonate (12a)

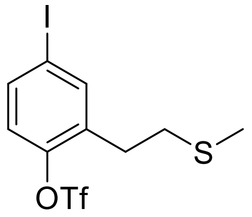

12

In a 50 mL round-bottom flask 2.00 g **12a** (6.80 mmol, 1.0 eq) were dissolved in 10 mL DCM. After cooling the solution to 0 °C 2.1 mL Et_3N (15.0 mmol, 2.2 eq) and 1.26 mL trifluoromethanesulfonic anhydride (Tf_2O) (7.48 mmol, 1.1 eq) were carefully added. After stirring for 5 min at 0 °C, the solution was allowed to warm to RT and stirred until quantitative conversion was detected via GC-MS (2 h). H_2O (100 mL) was added and the aqueous phase was extracted with DCM (3 x 100 mL). The combined organic phases were washed with sat. NaCl solution (1 x 60 mL), dried over Na_2SO_4 and concentrated in vacuum. The crude product was purified via flash column chromatography (60 g SiO_2, 3.0 x 16 cm, eluent: cyclohexane/EtOAc = 200/1, R_f = 0.23, UV and CAM).

Yield: 2.61 g (90%), colorless solid, $C_{10}H_{10}IO_3S$ [426.21 g/mol].

^{1}H NMR (300 MHz, $CDCl_3$): δ = 7.68 (d, $^4J_{H,H}$ = 1.9 Hz, 1H; H^{Ar}), 7.61 (dd, $^3J_{H,H}$ = 8.6 Hz, $^4J_{H,H}$ = 2.1 Hz, 1H; H^{Ar}), 6.99 (d, $^3J_{H,H}$ = 8.6 Hz, 1H; H^{Ar}), 2.93 (t, $^3J_{H,H}$ = 7.7 Hz, 2H; CH_2), 2.72 (t, $^3J_{H,H}$ = 7.6 Hz, 2H; CH_2), 2.13 (s, 3H; CH_3) ppm; **^{13}C NMR** (76 MHz, $CDCl_3$, APT): δ = 148.0 (C_q; C^{Ar}), 140.6 (C^{Ar}), 137.7 (C^{Ar}), 135.8 (C_q; C^{Ar}), 123.4 (C^{Ar}), 118.7 (q, $^1J_{C,F}$ = 320 Hz; CF_3), 93.5 (C_q; C^{Ar}), 34.0 (CH_2), 30.1 (CH_2), 15.7 (CH_3) ppm; **GC-MS** (EI, 70 eV; MT_50_S): t_R =

6.80 min; *m/z* (%): 426 (2) [M^+], 277 (100) [M^+−CF_3O_3S], 262 (11) [M^+− $C_2H_3F_3O_3S$]; **TLC**: R_f = 0.23 (cyclohexane/EtOAc = 200/1, UV and CAM); **m.p.**$^{exp.}$ = 42-44 °C; **HRMS** (EI): calcd for [M^+]: 425.9088; found: 425.9060.

3.4 Synthesis of Tyrosine building block

3.4.1 *tert*-Butyl(4-iodophenoxy)diphenylsilane

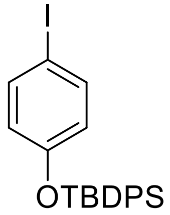

18

In a 250 mL round-bottom flask equipped with a drying tube 5.00 g p-iodophenol (22.7 mmol, 1.0 eq) and 3.87 g imidazole (56.8 mmol, 2.5 eq) were dissolved in 100 mL DCM. 7.1 mL *tert*-Butyl diphenylsilyl chloride (27.3 mmol, 1.2 eq) were added to the pale orange solution. A colorless precipitate was formed, and the suspension was stirred at RT overnight. When complete conversion was detected via GC-MS the reaction mixture was diluted with DCM (50 mL). The organic phase was washed with 1M HCl (1 x 100 mL), sat. $NaHCO_3$ solution (1 x 100 mL) and sat. NaCl solution (1 x 100 mL). Then the organic layer was dried over Na_2SO_4, filtered and concentrated to dryness under reduced pressure. The yellow, oily crude product was purified via flash column chromatography (200 g SiO_2, 5.5 x 13 cm, eluent: cyclohexane, R_f = 0.47, CAM).
Yield: 9.22 g (89%), colorless crystals, $C_{22}H_{23}IOSi$ [458.41 g/mol].

^{1}H NMR (300 MHz, $CDCl_3$): δ = 7.70-7.67 (m, 4H; H^{Ar}), 7.46-7.35 (m, 8H; H^{Ar}), 6.53 (d, $^3J_{H,H}$ = 8.8 Hz, 2H; H^{Ar}), 1.09 (s, 9H; CH_3) ppm; **^{13}C NMR** (76 MHz, $CDCl_3$, APT): δ = 155.7 (C_q; C^{Ar}), 139.2 (C^{Ar}), 135.6 (C^{Ar}), 132.6 (C_q; C^{Ar}), 130.2 (C^{Ar}), 128.0 (C^{Ar}), 122.3 (C^{Ar}), 83.6 (C_q; C^{Ar}), 26.6 (CH_3), 19.6 (CH) ppm; **GC-MS** (EI, 70 eV; MT_50_S): t_R = 9.28 min; *m/z* (%): 458 (5) [M^+], 401 (100) [M^+−C_4H_9], 273 (49) [M^+−C_4H_9I]; **m.p.**$^{exp.}$ = 37-38 °C.

Analytical data are in accordance with those reported.[10]

3.4.2 5-Iodo-2-((2-methoxyethoxy)methoxy)benzaldehyde

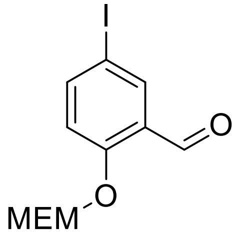

17

In a 100 mL round-bottom flask 3.00 g 2-hydroxy-5-iodobenzaldehyde (**13**) (12.1 mmol, 1.0 eq) were dissolved in 40 mL DCM. The pale-yellow solution was cooled to 0 °C in an ice bath. 2.9 mL DIPEA (16.9 mmol, 1.4 eq) followed by 1.9 mL MEM-Cl (16.9 mmol, 1.4 eq) were added. The solution turned intensively yellow and was stirred at RT overnight after complete addition of the reagents. After quantitative conversion was detected via GC-MS the reaction was quenched by the addition of sat. NH_4Cl solution (50 mL). The phases were separated, and the aqueous phase was extracted with DCM (3 x 50 mL). The combined organic layers were washed with sat. NaCl solution, dried over Na_2SO_4 and the solvent was removed under reduced pressure. The orange, oily crude product was purified via flash column chromatography (250 g SiO_2, 4.5 x 30 cm, eluent: cyclohexane/EtOAc = 4/1, R_f = 0.16, UV and CAM).

Yield: 3.43 g (84%), pale-yellow oil, $C_{11}H_{13}IO_4$ [336.13 g/mol].

^{1}H NMR (300 MHz, $CDCl_3$): δ = 10.39 (s, 1H; CHO), 8.13 (d, $^{4}J_{H,H}$ = 2.2 Hz, 1H; H^{Ar}), 7.82 (dd, $^{3}J_{H,H}$ = 8.8 Hz, $^{4}J_{H,H}$ = 2.3 Hz, 1H; H^{Ar}), 7.10 (d, $^{3}J_{H,H}$ = 8.8 Hz, 1H; H^{Ar}), 5.41 (s, 2H; CH_2), 3.90-3.87 (m, 2H; CH_2), 3.60-3.57 (m, 2H; CH_2), 3.40 (s, 3H; CH_3) ppm; **^{13}C NMR** (76 MHz, $CDCl_3$, APT): δ = 188.3 (CHO), 159.4 (C_q; C^{Ar}), 144.2 (C^{Ar}), 137.1 (C^{Ar}), 127.2 (C_q; C^{Ar}), 117.7 (C^{Ar}), 93.8 (CH_2), 84.7 (C_q; C^{Ar}), 71.6 (CH_2), 68.5 (CH_2), 59.2 (CH_3) ppm; **GC-MS** (EI, 70 eV; MT_50_S): t_R = 7.05 min; *m/z* (%): 336 (1) [M^+], 260 (7) [$M^+ - C_3H_7O_2$], 89 (100) [$M^+ - C_7H_4IO_2$], 59 (90) [$M^+ - C_8H_6IO_3$]; **TLC**: R_f = 0.16 (cyclohexane/EtOAc = 4/1, UV and CAM).

3.4.3 (4-((*tert*-Butyldiphenylsilyl)oxy)phenyl)(5-iodo-2-((2-methoxyethoxy)methoxy)phenyl)methanol

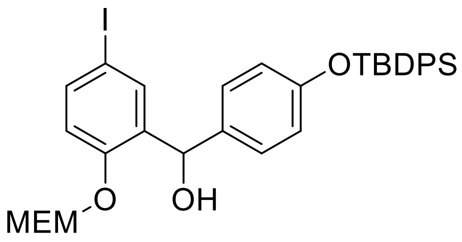

19

In a flame dried and argon flushed Schlenk-flask 2.00 g **18** (4.36 mmol, 1.0 eq) were dissolved in 20 mL dry THF. The colorless solution was cooled to -78 °C and 4.0 mL *t*BuLi solution (2.20M in THF) (8.73 mmol, 2.0 eq) were added. The yellow solution was stirred at -78 °C for 30 min. After quantitative iodide-lithium exchange had been detected via GC-MS, 1.47 g aldehyde **17** (4.36 mmol, 1.0 eq) were added. The color of the mixture turned orange and the reaction turned slightly cloudy. When full conversion was detected via TLC, the reaction was quenched by the addition of sat. NH_4Cl solution (50 mL) at -78 °C. After warming up to RT the phases were separated and the aqueous phase was diluted with H_2O (10 mL). Then the aqueous phase was extracted with DCM (5 x 50 mL). The combined organic layers were dried over Na_2SO_4, filtered and the solvent was removed under reduced pressure. The yellow, oily crude product was purified via flash column chromatography (200 g SiO_2, 4.0 x 27 cm, eluent: cyclohexane/EtOAc = 4/1, R_f = 0.31, UV and CAM).

Yield: 2.43 g (83%), colorless, highly viscous oil, $C_{33}H_{37}IO_5Si$ [668.64 g/mol].

^{1}H NMR (300 MHz, $CDCl_3$): δ = 7.70 (d, $^3J_{H,H}$ = 6.6 Hz, 5H; H^{Ar}), 7.45-7.33 (m, 7H; H^{Ar}), 7.06 (d, $^3J_{H,H}$ = 8.5 Hz, 2H; H^{Ar}), 6.84 (d, $^3J_{H,H}$ = 8.6 Hz, 1H; H^{Ar}), 6.70 (d, $^3J_{H,H}$ = 8.5 Hz, 2H; H^{Ar}), 5.87 (s, 1H; CH), 5.11 (q, $^3J_{H,H}$ = 7.0 Hz, 2H; CH_2), 3.58-3.36 (m, 4H; CH_2), 3.30 (s, 3H; CH_3), 1.09 (s, 9H; CH_3) ppm; **^{13}C NMR** (76 MHz, $CDCl_3$, APT): δ = 155.2 (C_q; C^{Ar}), 153.9 (C_q; C^{Ar}), 137.4 (C^{Ar}), 136.2 (C^{Ar}), 135.6 (C^{Ar}), 135.5 (C_q; C^{Ar}), 133.0 (C_q; C^{Ar}), 130.1 (C^{Ar}), 127.9 (C^{Ar}), 127.8 (C^{Ar}), 119.6 (C^{Ar}), 116.6 (C^{Ar}), 93.2 (CH_2), 85.0 (C_q; C^{Ar}), 71.6 (CH_2), 71.0 (CH), 67.9 (CH_2), 59.1 (CH_3), 26.7 (CH_3), 19.6 (C_q) ppm; **TLC**: R_f = 0.31 (cyclohexane/EtOAc = 4/1, UV and CAM).

3.4.4 2-(4-((*tert*-Butyldiphenylsilyl)oxy)benzyl)-4-iodophenol

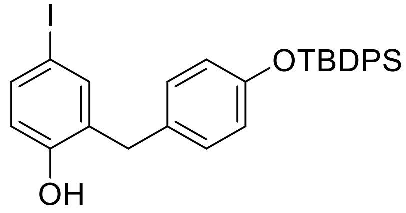

20a

In a 50 mL round-bottom flask 1.90 g **19** (2.84 mmol, 1.0 eq) were dissolved in 30 mL DCM. 3.6 mL Et_3SiH (22.7 mmol, 8.0 eq) and 1.3 mL trifluoroacetic acid (17.1 mmol, 6.0 eq) were added to this pale-yellow solution. The yellow solution was stirred at RT until full conversion was detected via TLC (48 h). Then the reaction was diluted with H_2O (50 mL) and extracted with DCM (4 x 50 mL). The combined organic layers were dried over Na_2SO_4, filtered and the solvent was removed under reduced pressure. The oily crude product was purified via flash column chromatography (100 g SiO_2, 150 g SiO_2, 4.0 x 23 cm, eluent: cyclohexane/EtOAc = 10/1, R_f = 0.23, UV and $FeCl_3$).

Yield: 1.09 g (68%), orange oil, $C_{29}H_{29}IO_2Si$ [564.54 g/mol].

^{1}H NMR (300 MHz, $CDCl_3$): δ = 7.73-7.70 (m, 4H; H^{Ar}), 7.45-7.31 (m, 8H; H^{Ar}), 6.92 (d, $^3J_{H,H}$ = 8.4 Hz, 2H; H^{Ar}), 6.70 (d, $^3J_{H,H}$ = 8.4 Hz, 2H; H^{Ar}), 6.54 (d, $^3J_{H,H}$ = 8.4 Hz, 1H; H^{Ar}), 4.80 (bs, 1H; OH), 3.79 (s, 2H, CH_2), 1.11 (s, 9H; CH_3) ppm; **^{13}C NMR** (76 MHz, $CDCl_3$, APT): δ = 154.5 (C_q; C^{Ar}), 154.0 (C_q; C^{Ar}), 139.3 (C^{Ar}), 136.5 (C^{Ar}), 135.7 (C^{Ar}), 133.1 (C_q; C^{Ar}), 131.3 (C_q; C^{Ar}), 130.4 (C_q; C^{Ar}), 130.0 (C^{Ar}), 129.6 (C^{Ar}), 127.9 (C^{Ar}), 120.1 (C^{Ar}), 118.1 (C^{Ar}), 82.8 (C_q; C^{Ar}), 35.4 (CH_2), 26.7 (CH_3), 19.6 (C_q) ppm; **TLC**: R_f = 0.23 (cyclohexane/EtOAc = 10/1, UV and $FeCl_3$); **HRMS** (EI): calcd for [M^+]: 564.0981; found: 564.0984.

3.4.5 2-(4-((*tert*-Butyldiphenylsilyl)oxy)benzyl)-4-iodophenyl trifluoromethanesulfonate

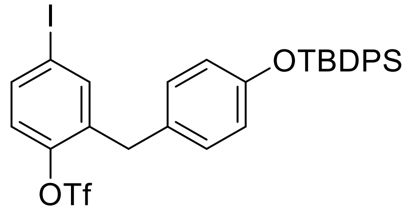

20

The compound was prepared according to general procedure 2.2 from 200 mg **20a** (354 µmol, 1.0 eq) and 70 µL Tf_2O (390 µmol, 1.1 eq) in 1 mL pyridine. The yellow, oily crude product was

purified via flash column chromatography (15 g SiO_2, 2.0 x 16 cm, eluent: cyclohexane/EtOAc = 200/1, R_f = 0.29, CAM).
Yield: 156 mg (63%), colorless oil, $C_{30}H_{28}F_3IO_4SSi$ [696.59 g/mol].

^{1}H NMR (300 MHz, $CDCl_3$): δ = 7.72 (d, $^3J_{H,H}$ = 6.4 Hz, 4H; H^{Ar}), 7.58 (dd, $^3J_{H,H}$ = 8.6 Hz, $^4J_{H,H}$ = 1.8 Hz, 1H; H^{Ar}), 7.44-7.36 (m, 7H; H^{Ar}), 6.99 (d, $^3J_{H,H}$ = 8.6 Hz, 1H; H^{Ar}), 6.88 (d, $^3J_{H,H}$ = 8.4 Hz, 2H; H^{Ar}), 6.73 (d, $^3J_{H,H}$ = 8.4 Hz, 2H; H^{Ar}), 3.88 (s, 2H; CH_2), 1.11 (s, 9H; CH_3) ppm; **^{13}C NMR** (76 MHz, $CDCl_3$, APT): δ = 154.6 (C_q; C^{Ar}), 147.7 (C_q; C^{Ar}), 146.1 (C_q; C^{Ar}), 140.5 (C^{Ar}), 137.1 (C^{Ar}), 136.9 (C_q; C^{Ar}), 135.6 (C^{Ar}), 132.9 (C_q; C^{Ar}), 129.9 (C^{Ar}), 129.9 (C^{Ar}), 127.8 (C^{Ar}), 123.1 (C^{Ar}), 118.6 (d[1], $^1J_{C,F}$ = 323 Hz; CF_3), 120.1 (C^{Ar}), 93.4 (C_q; C^{Ar}), 34.6 (CH_2), 26.5 (CH_3), 19.5 (C_q) ppm; **TLC**: R_f = 0.29 (cyclohexane/EtOAc = 200/1, UV and CAM); **HRMS** (EI): calcd for [M^+]: 696.0474; found: 696.0477.

3.5 Synthesis of Tryptophan building block

3.5.1 2-(Hydroxymethyl)-4-iodophenol

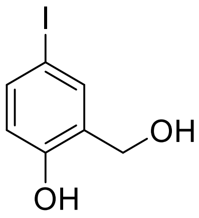

21

In a 50 mL round-bottom flask 228 mg $NaBH_4$ (6.05 mmol, 0.5 eq) were suspended in 30 mL *i*PrOH. The colorless suspension was cooled to 0 °C and 3.00 g 2-hydroxy-5-iodobenzaldehyde (**13**) (12.1 mmol, 1.0 eq) were added in small portions. After stirring the yellow suspension at RT for 2 h it turned into a colorless suspension. When full conversion was detected via TLC (4 h) the reaction was cooled again to 0 °C and quenched by the addition of 2M HCl (12 mL). The colorless solution was diluted with sat. NaCl solution (50 mL) and extracted with Et_2O (4 x 50 mL). The combined organic layers were dried over Na_2SO_4, filtered and the solvent was removed in vacuum. The crude product **21** was directly used in the following step without further purification.
Yield: 3.00 g (99%); pale-yellow powder, $C_7H_7IO_2$ [250.04 g/mol].

---
[1] only doublet observed (signal should give a quadruplet)

^{1}H NMR (300 MHz, [D$_6$]DMSO): δ = 9.66 (bs, 1H; OH), 7.55 (s, 1H; H^{Ar}), 7.34 (dd, $^{3}J_{H,H}$ = 8.3 Hz, $^{4}J_{H,H}$ = 1.6 Hz, 1H; H^{Ar}), 6.60 (d, $^{3}J_{H,H}$ = 8.4 Hz, 1H; H^{Ar}), 5.07 (bs, 1H; OH), 4.42 (s, 2H; CH$_2$) ppm; **^{13}C NMR** (76 MHz, [D$_6$]DMSO, APT): δ = 154.0 (C$_q$; C^{Ar}), 135.6 (C^{Ar}), 135.3 (C^{Ar}), 131.9 (C$_q$; C^{Ar}), 117.2 (C^{Ar}), 80.8 (C^{Ar}), 57.5 (CH$_2$) ppm; **TLC**: R$_f$ = 0.50 (cyclohexane/EtOAc = 3/1, UV and CAM); **m.p.**$^{exp.}$ = 135-137 °C (m.p.$^{lit.}$ = 139-140 °C).[11]

Analytical data are in accordance with those reported.[12]

3.5.2 2-((1*H*-Indol-3-yl)methyl)-4-iodophenol

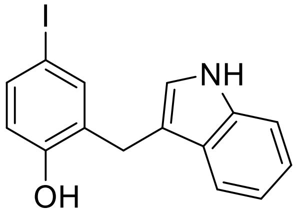

22a

In a flame dried and argon flushed Schlenk-flask 500 mg 2-(hydroxymethyl)-4-iodophenol (**21**) (2.00 mmol, 1.0 eq) and 235 mg indole (2.00 mmol, 1.0 eq) were dissolved in 2 mL DMF. 3Å molecular sieves were added to this pale-yellow solution and the reaction mixture was heated to 150 °C. The mixture turned darker overnight and became finally a dark brown solution. When quantitative conversion was detected via TLC the molecular sieves were removed by filtration and the filter cake was washed with Et$_2$O (2 x 50 mL) and water (2 x 50 mL). The phases were separated and the aqueous phase was extracted with Et$_2$O (3 x 50 mL). The combined organic layers were washed with sat. NaCl solution, dried over Na$_2$SO$_4$ and the solvent was removed under reduced pressure. The brown, oily crude product was purified via flash column chromatography (30 g SiO$_2$, 2.5 x 14 cm, eluent: cyclohexane/EtOAc = 5/1, R$_f$ = 0.19, UV and ninhydrin).
Yield: 498 mg (71%), orange powder, $C_{15}H_{12}INO_4$ [349.17 g/mol].

^{1}H NMR (300 MHz, [D$_6$]DMSO): δ = 10.83 (s, 1H; NH), 9.76 (s, 1H; OH), 7.47 (d, $^{3}J_{H,H}$ = 7.8 Hz, 1H; H^{Ar}), 7.36-7.25 (m, 3H; H^{Ar}), 7.14 (s, 1H; H^{Ar}), 7.06 (t, $^{3}J_{H,H}$ = 7.4 Hz, 1H; H^{Ar}), 6.94 (t, $^{3}J_{H,H}$ = 7.2 Hz, 1H; H^{Ar}), 6.67 (d, $^{3}J_{H,H}$ = 8.3 Hz, 1H; H^{Ar}), 3.91 (s, 2H; CH$_2$), ppm; **^{13}C NMR** (76 MHz, [D$_6$]DMSO, APT): δ = 154.8 (C$_q$; C^{Ar}), 137.8 (C^{Ar}), 136.3 (C$_q$; C^{Ar}), 135.1 (C^{Ar}), 131.3 (C$_q$; C^{Ar}), 127.0 (C$_q$; C^{Ar}), 123.4 (C^{Ar}), 120.9 (C^{Ar}), 118.5 (C^{Ar}), 118.3 (C^{Ar}), 117.6 (C^{Ar}), 112.8 (C$_q$; C^{Ar}), 111.4 (C^{Ar}), 80.8 (C$_q$; C^{Ar}), 24.2 (CH$_2$) ppm; **TLC**: R$_f$ = 0.19 (cyclohexane/EtOAc = 5/1,

UV and ninhydrin); **m.p.**$^{exp.}$ = 128-130 °C; **HRMS** (EI): calcd for [M^+]: 348.9964; found: 348.9971.

3.5.3 2-((1*H*-Indol-3-yl)methyl)-4-iodophenyl trifluoromethanesulfonate

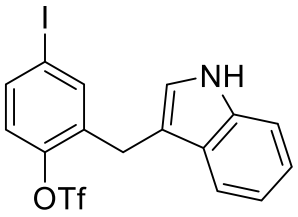

22

A 25 mL round-bottom flask was charged with 1.00 g indole-derivative **22a** (2.86 mmol, 1.0 eq) and 6 mL DCM. The pale-yellow solution was cooled to 0 °C in an ice bath and 500 µL 2,6-lutidine (4.30 mmol, 1.5 eq) were added. Then 690 µL Tf_2O (2.86 mmol, 1.0 eq) were added and a reddish-brown solution was formed. The reaction was stirred at 0 °C until full conversion was detected via TLC (cyclohexane/EtOAc = 5/1, R_f = 0.35, UV and ninhydrin). Then the reaction mixture was diluted with DCM (50 mL) and washed with H_2O (2 x 50 mL) as well as brine (1 x 50 mL). The organic phase was dried over Na_2SO_2, filtered and the solvent was removed under reduced pressure. The brown, oily crude product was purified via flash column chromatography (150 g SiO_2, 4.0 x 23 cm, eluent: cyclohexane/EtOAc = 10/1, R_f = 0.23, UV and ninhydrin).
Yield: 1.09 g (78%), pale brown oil, $C_{16}H_{11}F_3INO_3S$ [481.23 g/mol].

^{1}H NMR (300 MHz, $CDCl_3$): δ = 8.08 (bs, 1H; NH), 7.60-7.58 (m, 2H; H^{Ar}), 7.46 (d, $^3J_{H,H}$ = 7.8 Hz, 1H; H^{Ar}), 7.40 (d, $^3J_{H,H}$ = 8.1 Hz, 1H; H^{Ar}), 7.23 (dt, $^3J_{H,H}$ = 8.3 Hz, $^4J_{H,H}$ = 1.2 Hz, 2H; H^{Ar}), 7.15-7.09 (m, 1H; H^{Ar}), 7.06-7.01 (m, 2H; H^{Ar}), 4.16 (s, 2H; CH_2), ppm; **^{13}C NMR** (76 MHz, $CDCl_3$), APT): δ = 147.8 (C_q; C^{Ar}), 140.3 (C^{Ar}), 137.1 (C^{Ar}), 136.6 (C_q; C^{Ar}), 136.4 (C_q; C^{Ar}), 127.0 (C_q; C^{Ar}), 123.1 (C^{Ar}), 123.1 (C^{Ar}), 122.4 (C^{Ar}), 118.6 (q, $^1J_{C,F}$ = 320 Hz; CF_3), 119.9 (C^{Ar}), 118.7 (C^{Ar}), 112.0 (C_q; C^{Ar}), 111.3 (C^{Ar}), 93.4 (C_q; C^{Ar}), 25.4 (CH_2) ppm; **TLC**: R_f = 0.23 (cyclohexane/EtOAc = 10/1, UV and ninhydrin), **HRMS** (EI): calcd for [M^+-H]: 479.9384; found: 479.9380.

3.6 Synthesis of Arginine building block

3.6.1 6-Iodochroman-2-one

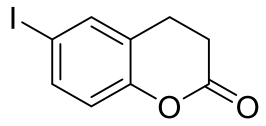

23a

The compound was synthesized according to general procedure 2.1 from 5.00 g dihydrocoumarin (33.8 mmol, 1.0 eq) dissolved in 35 mL DCM and 5.48 g ICl (33.8 mmol, 1.0 eq) dissolved in 35 mL DCM. The yellow, solid crude was recrystallized from DCM/cyclohexane (1/4).[2]
Yield: 8.28 g (90%), colorless powder, $C_9H_7IO_2$ [274.06 g/mol].

^{1}H NMR (300 MHz, $CDCl_3$): δ = 7.56-7.53 (m, 2H; H^{Ar}), 6.81 (d, $^3J_{H,H}$ = 8.1 Hz, 1H; H^{Ar}), 2.97 (t, $^3J_{H,H}$ = 7.2 Hz, 2H; CH_2), 2.76 (t, $^3J_{H,H}$ = 7.1 Hz, 2H; CH_2) ppm; **^{13}C NMR** (76 MHz, $CDCl_3$, APT): δ = 167.8 (C_q; CO), 152.1 (C_q; C^{Ar}), 137.4 (C^{Ar}), 136.9 (C^{Ar}), 125.2 (C_q; C^{Ar}), 119.2 (C^{Ar}), 87.6 (C_q; C^{Ar}), 28.9 (CH_2), 23.5 (CH_2) ppm; **GC-MS** (EI, 70 eV; MT_50_S): t_R = 6.71 min; *m/z* (%): 145 (31) [M^+], 118 (100) [M^+−CN], 51 (7) [M^+−C_6H_5O]; **m.p.**$^{exp.}$ = 134-136 °C, (m.p.$^{lit.}$ = 133-134 °C).[13]

Analytical data are in accordance with those reported.[2]

3.6.2 Methyl 3-(2-hydroxy-5-iodophenyl)propanoate

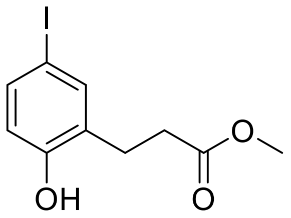

23b

In a flame dried and argon flushed Schlenk-flask 2.00 g 6-iodochroman-2-one (**23a**) (7.30 mmol, 1.0 eq) were suspended in 140 mL MeOH. 2 mL conc. H_2SO_4 were added to this colorless suspension. The suspension dissolved and the resulting colorless solution was warmed to 55 °C and stirred overnight at this temperature. After full conversion (24 h) was detected via GC-MS, the brown solution was neutralized by adding sat. $NaHCO_3$ solution (50 mL) and the solvent was removed in vacuum. The brown residue was diluted with H_2O (100 mL), and extracted with DCM

(3 x 100 mL). The combined organic layers were dried over $MgSO_4$, filtered and concentrated in vacuum. The crude product was purified via flash column chromatography (250 g SiO_2, 6.0 x 17 cm, eluent: cyclohexane/EtOAc = 5/1, R_f = 0.18, UV and CAM) and **23b** was isolated as a light-yellow oil.

Yield: 1.70 g (76%), light-yellow oil, $C_{10}H_{11}IO_3$ [306.10 g/mol].

^{1}H NMR (300 MHz, $CDCl_3$): δ = 7.39-7.35 (m, 3H; H^{Ar}, OH), 6.65-6.62 (m, 1H; H^{Ar}), 3.66 (s, 3H; CH_3), 2.82 (t, $^3J_{H,H}$ = 5.9 Hz, 2H; CH_2), 2.69 (t, $^3J_{H,H}$ = 5.9 Hz, 2H; CH_2) ppm; **^{13}C NMR** (76 MHz, $CDCl_3$, APT): δ = 176.2 (C_q; CO), 154.6 (C_q; C^{Ar}), 139.2 (C^{Ar}), 137.0 (C^{Ar}), 130.4 (C_q; C^{Ar}), 119.9 (C^{Ar}), 82.9 (C_q; C^{Ar}), 52.6 (CH_3), 35.0 (CH_2), 24.5 (CH_2) ppm; **GC-MS** (EI, 70 eV; MT_50_S): t_R = 6.92 min; *m/z* (%): 306 (17) [M^+], 274 (100) [$M^+ - OCH_3$], 246 (64) [$M^+ - C_2H_3O_2$], 91 (0.31) [$M^+ - C_4H_7O_2I$]; **TLC**: R_f = 0.18 (cyclohexane/EtOAc = 5/1, UV and CAM); **HRMS** (EI): calcd for [M^+]: 305.9753; found: 305.9766.

3.6.3 Methyl 3-(5-iodo-2-(((trifluoromethyl)sulfonyl)oxy)phenyl)propanoate

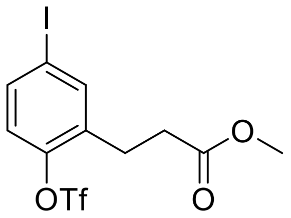

23

Compound **23** was prepared according to procedure 2.2 from 800 mg phenol derivative **23b** (2.61 mmol, 1.0 eq) and 700 µL Tf_2O (2.87 mmol, 1.1 eq) in 3.3 mL pyridine. Quantitative conversion was detected after 2 h. After purification via flash column chromatography (100 g, SiO_2, 5.0 x 10 cm, eluent: cyclohexane/EtOAc = 20/1, R_f = 0.26, UV and CAM) compound **23** was isolated as a colorless oil.

Yield: 966 mg (84%), colorless oil, $C_{11}H_{10}F_3IO_5S$ [438.16 g/mol].

^{1}H NMR (300 MHz, $CDCl_3$): δ = 7.69 (d, $^4J_{H,H}$ = 2.1 Hz, 1H; H^{Ar}), 7.62 (dd, $^3J_{H,H}$ = 8.7 Hz, $^4J_{H,H}$ = 2.1 Hz, 1H; H^{Ar}), 7.01 (d, $^3J_{H,H}$ = 8.7 Hz, 1H; H^{Ar}), 3.69 (s, 3H; CH_3), 2.99 (t, $^3J_{H,H}$ = 7.7 Hz, 2H; CH_2), 2.65 (t, $^3J_{H,H}$ = 7.7 Hz, 2H; CH_2) ppm; **^{13}C NMR** (76 MHz, $CDCl_3$, APT): δ = 172.3 (C_q; C^{Ar}), 147.9 (C_q; C^{Ar}), 140.3 (C^{Ar}), 137.6 (C^{Ar}), 135.8 (C_q; C^{Ar}), 123.4 (C^{Ar}), 118.7 (q, $^1J_{C,F}$ = 318 Hz; CF_3), 93.4 (C_q; CN), 52.0 (CH_3), 33.7 (CH_2), 25.0 (CH_2) ppm; **GC-MS** (EI, 70 eV;

MT_50_S): t_R = 6.65 min; *m/z* (%): 438 (4) [M^+], 407 (11) [M^+-OCH_3], 289 (100) [$M^+-CF_3O_3S$], 91 (34) [$M^+-C_5H_3F_3IO_4S$]; **TLC**: R_f = 0.26 (cyclohexane/EtOAc = 20/1, UV and CAM); **HRMS** (EI): calcd for [M^+]: 437.9246; found: 437.9276.

3.6.4 2-(3-Hydroxypropyl)-4-iodophenyl trifluoromethanesulfonate

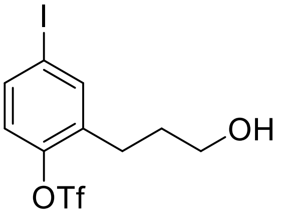

24

In a flame dried and argon flushed Schlenk-flask 2.00 g ester **23** (4.56 mmol, 1.0 eq) were dissolved in 7 mL dry DCM. This solution was cooled to -78 °C and 9.2 mL diisobutylaluminium hydride (DIBAL-H) (1.0M in toluene) (9.20 mmol, 2.0 eq) were slowly added via a dropping funnel. The colorless solution was warmed up to RT. After stirring for 2 h the solution was cooled again to -78 °C and quenched by the addition of MeOH (6 mL). Saturated Rochelle-salt solution (20 mL) was added, and the emulsion was stirred until phase separation occurred (overnight). The phases were separated, and the aqueous phase was extracted with DCM (3 x 100 mL). The combined organic layers were dried over Na_2SO_4 and the solvent was removed under reduced pressure. The colorless, oily crude product was purified via flash column chromatography (75 g SiO_2, 3.0 x 20 cm, eluent: cyclohexane/EtOAc = 5/1, R_f = 0.22, UV and CAM).

Yield: 1.83 g (98%), colorless oil, $C_{10}H_{10}F_3IO_4S$ [410.15 g/mol].

^{1}H NMR (300 MHz, $CDCl_3$): δ = 7.69 (d, $^4J_{H,H}$ = 1.9 Hz, 1H; H^{Ar}), 7.59 (dd, $^3J_{H,H}$ = 8.6 Hz, $^4J_{H,H}$ = 2.1 Hz, 1H; H^{Ar}), 6.99 (d, $^3J_{H,H}$ = 8.6 Hz, 1H; H^{Ar}), 3.69 (t, $^3J_{H,H}$ = 6.1 Hz, 1H; CH_2), 2.77 (t, $^3J_{H,H}$ = 7.8 Hz, 2H; CH_2), 1.93-1.83 (m, 2H; CH_2) ppm; **^{13}C NMR** (76 MHz, $CDCl_3$, APT): δ = 148.1 (C_q; C^{Ar}), 140.4 (C^{Ar}), 137.3 (C_q; C^{Ar}), 137.1 (C^{Ar}), 123.3 (C^{Ar}), 118.7 (q, $^1J_{C,F}$ = 320 Hz; CF_3), 93.5 (C_q; C^{Ar}), 61.9 (CH_2), 32.6 (CH_2), 26.3 (CH_2) ppm; **TLC**: R_f = 0.22 (cyclohexane/EtOAc = 5/1, UV and CAM); **HRMS** (EI): calcd for [M^+]: 409.9297; found: 409.9318.

3.6.5 2-(3-(2,3-Bis(*tert*-butoxycarbonyl)guanidino)propyl)-4-iodophenyl trifluoromethanesulfonate

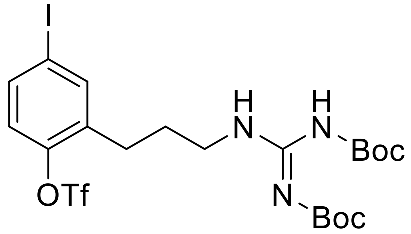

26

In a 50 mL round-bottom flask 1.3 g **24** (3.17 mmol, 1.0 eq) were dissolved in 15 mL THF. The colorless solution was cooled to 0 °C and 570 µL DIPEA (3.17 mmol, 1.0 eq), 998 mg PPh_3 (3.80 mmol, 1.2 eq), 756 µL DIAD (3.80 mmol, 1.2 eq) and 820 µL diphenyl phosphoryl azide (DPPA) (3.80 mmol, 1.2 eq) were added. The pale-yellow suspension was stirred at RT for 3 h. When quantitative conversion of the alcohol was detected via TLC 1.08 g PPh_3 (4.12 mmol, 1.3 eq) dissolved in 2 mL THF were added and the reaction was stirred overnight at RT. After adding 1 mL H_2O the reaction was warmed to 50 °C and stirred until full conversion of the azide-intermediate was detected via GC-MS. The solvent was removed under reduced pressure and the oily residue (amine-intermediate) was dissolved in 16 mL DMF. To this pale-yellow solution 986 mg *N*,*N*'-di-*Boc*-1*H*-pyrazole-1-carboxamidine (**25**) (3.18 mmol, 1.0 eq) were added. The reaction was stirred at RT for 4 h. When quantitative conversion of the amine was detected via TLC, the reaction was diluted with Et_2O (100 mL) and washed with water (2 x 50 mL). The organic phase was dried over Na_2SO_4, filtered and the solvent was removed under reduced pressure. The pale-yellow, oily crude product was purified via flash column chromatography (250 g SiO_2, 5.5 x 18 cm, eluent: cyclohexane/EtOAc = 15/1, R_f = 0.15, UV and CAM).

Yield: 1.14 g (55%), colourless solid, $C_{21}H_{29}F_3IN_3O_7S$ [651.44 g/mol].

^{1}H NMR (300 MHz, $CDCl_3$): δ = 11.49 (bs, 1H; NH), 8.41 (bs, 1H; NH), 7.67 (s, 1H; H^{Ar}), 7.59 (dd, $^3J_{H,H}$ = 8.6 Hz, $^4J_{H,H}$ = 1.8 Hz, 1H; H^{Ar}), 6.98 (d, $^3J_{H,H}$ = 8.6 Hz, 1H; H^{Ar}), 3.59 (q, $^3J_{H,H}$ = 6.6 Hz, 2H; CH_2), 2.72 (t, $^3J_{H,H}$ = 7.8 Hz, 2H; CH_2), 1.96-1.86 (m, 2H; CH_2), 1.50 (s, 9H; CH_3), 1.49 (s, 9H; CH_3) ppm; **^{13}C NMR** (76 MHz, $CDCl_3$, APT): δ = 163.6 (C_q; CN), 156.4 (C_q; CO), 153.5 (C_q; CO), 1487.9 (C_q; C^{Ar}), 140.2 (C^{Ar}), 137.3 (C^{Ar}), 136.6 (C_q; C^{Ar}), 123.4 (C^{Ar}), 118.7 (q, $^1J_{C,F}$ = 320 Hz; CF_3), 93.5 (C_q; C^{Ar}), 83.4 (C_q), 79.5 (C_q), 40.3 (CH_2), 29.2 (CH_2), 28.4 (CH_3), 28.2 (CH_3), 27.2 (CH_2) ppm; **TLC**: R_f = 0.15 (cyclohexane/EtOAc = 15/1, UV and ninhydrin); **m.p.**$^{exp.}$ = 105-108 °C; **HRMS** (EI): calcd for [M^+]: 651.0723; found: 651.0693.

4 Screening for optimal coupling conditions

4.1 General reaction scheme

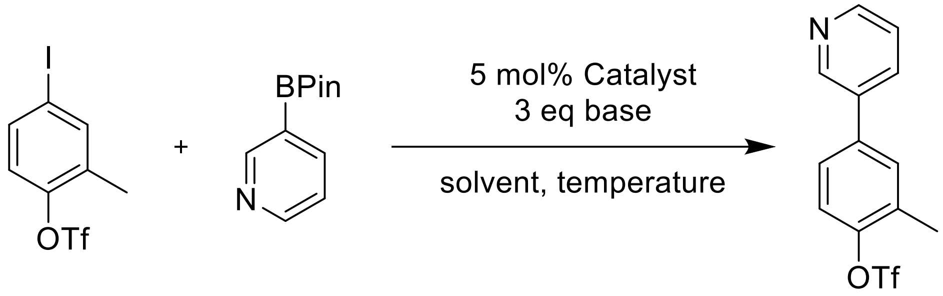

27 1.0 eq **28** 1.05 eq **29**

4.2 Base anion screening[a]

Entry	Anion	2 h		24 h	
		Conversion[b]	Selectivity[b]	Conversion[b]	Selectivity[b]
1	K_2CO_3	48%	> 99%	> 99%	> 99%
2	K_3PO_4	73%	95%	> 99%	95%
3	KOH	62%	86%	69%	52%
4	KF	< 5%	n. d.	11%	> 99%
5	KO*i*Pr	93%	89%	93%	88%
6	KOMe	74%	86%	51%	86%
7	KO*t*Bu	62%	53%	87%	71%
8	KOAc	< 5%	n. d.	< 5%	n. d.

[a] $PdCl_2$(dppf), 1,2-DME, 80 °C, [b] Conversion and selectivity were monitored via GC-MS.

4.3 Solvent screening[a]

Entry	Solvent	2 h		24 h	
		Conversion[b]	Selectivity[b]	Conversion[b]	Selectivity[b]
1	1,2-DME	20%	> 99%	78%	96%
2	THF	4%	> 99%	44%	> 99%
3	1,4-Dioxane	< 5%	n. d.	83%	> 99%

4	DMF	> 99%	> 99%	> 99%	56%
5	MeOH	< 5%	n. d.	< 5%	n. d.
6	CH_3CN	41%	> 99%	> 99%	> 99%
7	DMSO	> 99%	> 99%	> 99%	< 5%

[a] $PdCl_2$(dppf), K_2CO_3, 80 °C, [b] Conversion and selectivity were monitored via GC-MS.

4.4 Base cation screening[a]

Entry	Cation	2 h		24 h	
		Conversion[b]	Selectivity[b]	Conversion[b]	Selectivity[b]
1	K_2CO_3	> 99%	> 99%	> 99%	24%
2	Na_2CO_3	98%	> 99%	> 99%	> 99%
3	Cs_2CO_3	> 99%	> 99%	> 99%	< 5%
4	Li_2CO_3	< 5%	n. d.	31%	> 99%
5	Ag_2CO_3	> 99%	> 99%	> 99%	> 99%

[a] $PdCl_2$(dppf), DMF, 80 °C, [b] Conversion and selectivity were monitored via GC-MS.

4.5 Catalyst screening[a]

Entry	Pd-source	Ligand	Conversion 2 h[b]	Conversion 6 h[b]
1	$Pd_2(dba)_3$	$P(tBu)_3$	62%	96%
2	$[Pd(\mu\text{-}Br)P(tBu)_3]_2$		55%	80%
3	$Pd(PPh_3)_4$		10%	20%
4	$Pd(OAc)_2$	SPhos	30%	60%
5	$Pd(OAc)_2$	XPhos	14%	31%
6	$PdCl_2(PPh_3)_2$		38%	75%
7	$Pd_2(dba)_3$	PCy_3	< 5%	6%
8	$Pd_2(dba)_3$	PPh_3	13%	20%
9	$Pd_2(dba)_3$	$P(o\text{-}Tol)_3$	69%	96%
10	$PdCl_2$(dppf)		90%	>99%

[a] K_2CO_3, CH_3CN, 70 °C, [b] Conversion and selectivity were monitored via GC-MS.

4.6 2-Methyl-4-(pyridin-3-yl)phenyl trifluoromethanesulfonate

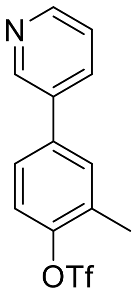

29

Compound **29** was prepared according to procedure 2.3 from 1.40 g pyridine boronic acid ester **28** (6.83 mmol, 1.0 eq), 1.89 g K_2CO_3 (13.7 mmol, 2.0 eq), 220 mg $PdCl_2$(dppf) (273 µmol, 4 mol%) and 2.50 g core building block **27** (6.83 mmol, 1.0 eq) in 50 mL dry, degassed DMF. After quantitative conversion was detected via GC-MS (2 h) the catalyst was removed by filtration through a pad of silica gel (eluted with 400 mL EtOAc). The organic phase was washed with sat. NH_4Cl solution (2 x 100 mL), dried over Na_2SO_4, filtered and the solvent was removed under reduced pressure. The crude product was purified via flash column chromatography (200 g SiO_2, 4.5 x 24 cm, eluent: cyclohexane/EtOAc = 3/1, R_f = 0.20, UV and CAM).
Yield: 1.74 g (80%), yellow oil, $C_{13}H_{10}F_3NO_3S$ [317.28 g/mol].

^{1}H NMR (300 MHz, $CDCl_3$): δ = 8.82 (s, 1H; H^{Ar}), 8.63 (d, $^4J_{H,H}$ = 3.8 Hz, 1H; H^{Ar}), 7.84 (d, $^3J_{H,H}$ = 7.9 Hz, 1H; H^{Ar}), 7.50-7.33 (m, 4H; H^{Ar}), 2.46 (s, 3H; CH_3) ppm; **^{13}C NMR** (76 MHz, $CDCl_3$, APT): δ = 149.2 (C^{Ar}), 148.5 (C_q; C^{Ar}), 148.3 (C^{Ar}), 138.2 (C_q; C^{Ar}), 135.1 (C_q; C^{Ar}), 134.5 (C^{Ar}), 131.8 (C_q; C^{Ar}), 131.0 (C^{Ar}), 126.5 (C^{Ar}), 123.7 (C^{Ar}), 122.1 (C^{Ar}), 118.8 (q, $^1J_{C,F}$ = 320 Hz; CF_3), 16.6 (CH_3) ppm; **GC-MS** (EI, 70 eV; MT_50_S): t_R = 6.60 min; *m/z* (%): 317 (17) [M^+], 184 (100) [M^+−CF_3O_2S]; **TLC**: R_f = 0.20 (cyclohexane/EtOAc = 3/1, UV and CAM), **HRMS** (EI): calcd for [M^++H]: 318.0412; found: 318.0406.

4.7 General reaction scheme for OTf-coupling

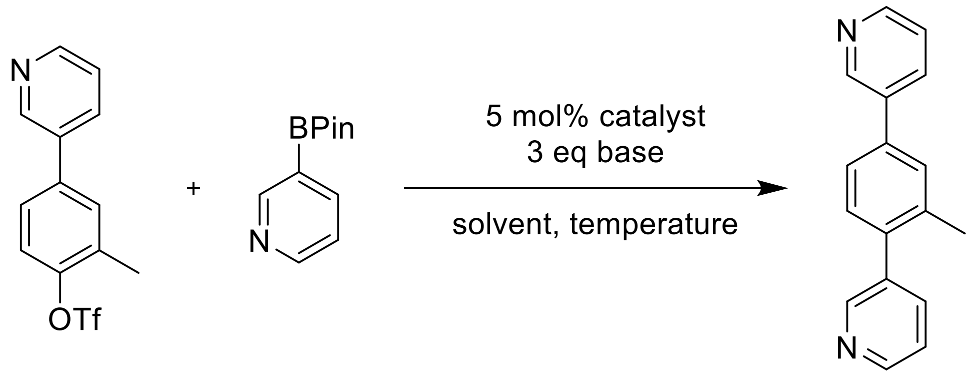

29 1.0 eq

28 1.05 eq

30

4.8 Base and solvent screening[a]

Entry	Base	Solvent	2 h		24 h	
			Conversion[b]	Selectivity[b]	Conversion[b]	Selectivity[b]
1	Cs_2CO_3	DMF	> 99%	> 99%	> 99%	96%
2	Cs_2CO_3	CH_3CN	> 99%	> 99%	> 99%	> 99%
3	K_3PO_4	DMF	> 99%	> 99%	> 99%	> 99%
4	NaOEt	DMF	> 99%	54%	> 99%	< 5%
5	NaOEt	EtOH	88%	7%	> 99%	13%

[a] $PdCl_2$(dppf), 70 °C, [b] Conversion and selectivity were monitored via GC-MS.

4.9 Catalyst screening[a]

Entry	Pd-source	Ligand	2 h		24 h	
			Conversion[b]	Selectivity[a]	Conversion[b]	Selectivity[a]
1	$Pd_2(dba)_3$	P(*t*Bu)$_3$	< 5%	n. d.	>99%	< 5%
2	[Pd(μ-Br)P(*t*Bu)$_3$]$_2$		31%	< 5%	>99%	11%
3	$Pd(PPh_3)_4$		96%	95%	>99%	97%
4	$Pd(OAc)_2$	SPhos	>99%	84%	>99%	82%
5	$Pd(OAc)_2$	XPhos	< 5%	n. d.	>99%	24%
6	$PdCl_2(PPh_3)_2$		>99%	>99%	>99%	>99%
7	$Pd_2(dba)_3$	PCy_3	>99%	>99%	>99%	>99%
8	$Pd_2(dba)_3$	PPh_3	67%	93%	>99%	95%

9	$Pd_2(dba)_3$	$P(o\text{-}Tol)_3$	< 5%	n. d.	74%	32%
10	$PdCl_2(dppf)$		>99%	>99%	>99%	>99%

[a] Cs_2CO_3, CH_3CN, 70 °C, [b] Conversion and selectivity were monitored via GC-MS.

4.10 3,3'-(2-Methyl-1,4-phenylene)dipyridine

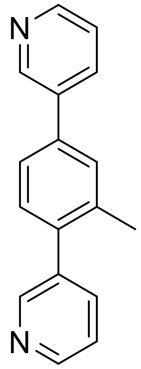

30

Compound **30** was prepared according to procedure 2.4 from 353 mg pyridine boronic acid ester **28** (1.72 mmol, 2.1 eq), 801 mg Cs_2CO_3 (2.46 mmol, 3.0 eq), 60 mg $PdCl_2$(dppf) (82 µmol, 10 mol%) and 300 mg core building block **27** (819 µmol, 1.0 eq) in 5 mL dry, degassed CH_3CN. After quantitative conversion was detected via GC-MS (20 h) the catalyst was removed by filtration through a pad of silica gel (eluted with 100 mL MeOH). The solvent was removed under reduced pressure and the crude product was purified via flash column chromatography (25 g SiO_2, 3.0 x 15 cm, eluent: cyclohexane/EtOAc = 1/3, R_f = 0.17, UV and CAM).
Yield: 162 mg (80%), brown oil, $C_{17}H_{14}F_3N_2$ [246.31 g/mol].

^{1}H NMR (300 MHz, $CDCl_3$): δ = 8.87 (s, 1H; H^{Ar}), 8.60 (m, 3H; 3x H^{Ar}), 7.91 (d, $^3J_{H,H}$ = 7.7 Hz, 1H; H^{Ar}), 7.68 (d, $^3J_{H,H}$ = 7.6 Hz, 1H; H^{Ar}), 7.54-7.43 (m, 2H; 2x H^{Ar}), 7.42-7.27 (m, 3H; 3x H^{Ar}), 2.33 (s, 3H; CH_3) ppm; **^{13}C NMR** (76 MHz, $CDCl_3$, APT): δ = 149.6 (C^{Ar}), 148.3 (C^{Ar}), 148.2 (C^{Ar}), 148.0 (C^{Ar}), 138.0 (C_q; C^{Ar}), 137.6 (C_q; C^{Ar}), 137.0 (C_q; C^{Ar}), 136.7 (C^{Ar}), 136.6 (C_q; C^{Ar}), 136.3 (C_q; C^{Ar}), 134.7 (C^{Ar}), 130.7 (C^{Ar}), 129.4 (C^{Ar}), 124.9 (C^{Ar}), 123.8 (C^{Ar}), 123.3 (C^{Ar}), 20.6 (CH_3) ppm**; GC-MS** (EI, 70 EV; MT_50_S): t_R = 8.141 min, *m/z* (%) = 246 (100) [M^+]; **TLC:** R_f = 0.20 (cyclohexane/EtOAc = 1/4, UV and CAM); **HRMS** (MALDI): calcd *(m/z)* for [M^+]: 246.1157; found: 246.1158.

5 Experimental Procedures and Analytical Data for Teraryl Synthesis

5.1 3-Bromo-5-isopropylpyridine

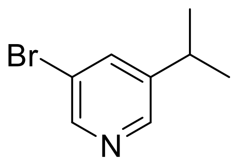

31a

A two-neck round-bottom flask was charged with 4.62 g Zn-powder (70.6 mmol, 2.0 eq). Subsequently the flask was evacuated, heated with a heat gun and after cooling to RT back flushed with argon three times. The Zn was suspended in 10 mL dry THF and 0.3 mL 1,2-dibromoethane (3.53 mmol, 0.05 eq) were added. The mixture was heated to reflux temperature and cooled again to RT for three times. After the third cycle, 450 µL TMSCl (3.53 mmol, 0.05 eq) were added and stirred at RT for 10 min. A solution of 3.50 mL 2-iodopropane (35.3 mmol, 1.0 eq) in 10 mL dry THF was added slowly via a dropping funnel. A water bath was used to keep the reaction at RT. When addition was finished, the reaction was stirred at RT for another 2 h. The conversion of halide was measured by GC-FID. For the GC-sample an aliquot of the reaction mixture was quenched with satd. NH_4Cl solution and extracted with DCM. The concentration of *i*PrZnI was not determined.

A flame dried and argon flushed Schlenk-flask was charged with 5.33 g 3,5-dibromopyridine (22.5 mmol, 1.0 eq), 181 mg $PdCl_2$(dppf) (225 µmol, 0.01 eq) and 20 mL dry THF. The previously prepared organozinc solution (1.0 eq) was added to this orange suspension and the brown solution was stirred at 70 °C overnight (16 h). At 95% conversion (16 h) already 10% dialkylation was detected. The catalyst was removed by filtration through a pad of silica gel and the product was eluted with EtOAc (3 x 250 mL). The solvent was removed under reduced pressure and the brown oil was purified via flash column chromatography (200 g SiO_2, 5.0 x 22 cm, eluent: cyclohexane/EtOAc = 9/1, R_f = 0.30, UV and CAM).

Yield: 2.34 g (52%), yellow oil, $C_8H_{10}BrN$ [200.08 g/mol].

^{1}H NMR (300 MHz, $CDCl_3$): δ = 8.63 (s, 1H; H^{Ar}), 8.42 (d, $^3J_{H,H}$ = 5.0 Hz, 1H; H^{Ar}), 7.18 (d, $^3J_{H,H}$ = 5.0 Hz, 1H; H^{Ar}), 3.30 (h, $^3J_{H,H}$ = 6.8 Hz, 1H; CH), 1.24 (d, $^3J_{H,H}$ = 6.9 Hz, 6H; CH_3) ppm; **^{13}C NMR** (76 MHz, $CDCl_3$, APT): δ = 156.1 (C_q; C^{Ar}), 152.0 (C^{Ar}), 148.7 (C^{Ar}), 123.0 (C_q; C^{Ar}),

121.9 (C^{Ar}), 32.7 (CH), 22.0 (CH_3) ppm; **GC-MS** (EI, 70 eV; MT_50_S): t_R = 4.72 min; *m/z* (%): 203 (76) [M^+], 201 (77) [M^+], 186 (91) [M^+−CH_3], 184 (93) [M^+−CH_3], 120 (19) [M^+−Br], 104 (100) [M^+−CH_3Br]; **TLC:** R_f = 0.30 (cyclohexane/EtOAc = 9/1, UV and CAM); **HRMS** (EI): calcd for [M^+]: 198.9997; found: 199.0001.

5.2 3-Isopropyl-5-(4,4,5,5-tetramethyl-1,3,2-dioxaborolan-2-yl)pyridine

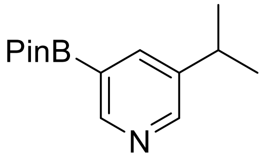

31

A Schlenk-flask was charged with 1.67 g 3-bromo-5-isopropylpyridine (**31a**) (8.35 mmol, 1.0 eq) dissolved in 20 mL dry THF. The reaction mixture was cooled to 0 °C and 6.6 mL *i*PrMgCl.LiCl solution (1.51 M in THF) (10.0 mmol, 1.2 eq) were added dropwise. When complete metal-halogen exchange was detected via GC-MS (an aliquot of the reaction mixture was quenched with satd. NH_4Cl solution and extracted with DCM after 2 h), 2.3 mL PinBO*i*Pr (2.10 g, 11.3 mmol, 1.4 eq) was added to the reaction mixture. The reaction was allowed to warm up in the cooling bath overnight and full conversion was detected via GC-MS (24 h). The reaction mixture was quenched by the addition of 50 mL satd. NH_4Cl solution. The phases were separated and the aqueous layer extracted with DCM (4 x 50 mL). The combined organic layers were washed with brine (1 x 50 mL), dried over Na_2SO_4 and concentrated in vacuum. The crude product was purified via recrystallization from pentane.

Yield: 482 mg (21%), colorless powder, $C_{14}H_{22}BNO_2$ [247.15 g/mol].

^{1}H NMR (300 MHz, $CDCl_3$): δ = 8.82 (s, 1H; H^{Ar}), 8.54 (d, $^3J_{H,H}$ = 5.2 Hz, 1H; H^{Ar}), 7.18 (d, $^3J_{H,H}$ = 5.2 Hz, 1H; H^{Ar}), 3.62 (h, $^3J_{H,H}$ = 6.8 Hz, 1H; CH), 1.35 (s, 12H; CH_3), 1.21 (d, $^3J_{H,H}$ = 6.9 Hz, 6H; CH_3) ppm; **^{13}C NMR** (76 MHz, $CDCl_3$, APT): δ = 164.4 (C_q; C^{Ar}), 156.3 (C^{Ar}), 151.9 (C^{Ar}), 83.9 (C_q), 31.5 (CH), 24.9 (CH_3), 23.6 (CH_3) ppm;[2] **GC-MS** (EI, 70 eV; MT_50_XS): t_R = 14.91 min; *m/z* (%): 274 (16) [M^+], 232 (16) [M^+−CH_3], 147 (100) [M^+− $C_6H_{12}O$], 132 (29) [M^+−$C_6H_{13}O_2$]; **m.p.**$^{exp.}$ = 81-82 °C; **HRMS** (EI): calcd (*m/z*) for [M^+]: 247.1746; found: 247.1751.

[2] Signal for the quaternary *ipso*-pyridine carbon (C_q; C^{Ar}) at the boronic acid pinacol ester function was not observed.

5.3 Gly-Met-Val

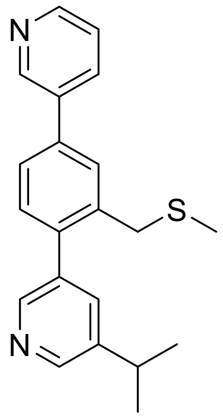

32

32 was prepared according to general procedure 2.3 from 51.6 mg pyridine building block **28** (252 µmol, 1.1 eq), 65.3 mg K_2CO_3 (473 µmol, 2.0 eq), 9.2 mg $PdCl_2$(dppf) (13 µmol, 5 mol%) and 104 mg **12** (245 µmol, 1.0 eq) in 2 mL dry, degassed CH_3CN. The crude product was purified via column chromatography (8 g SiO_2, 2.5 x 10 cm, eluent: cyclohexane/EtOAc = 2/1) to deliver 77 mg (83%) diaryl intermediate.

The second coupling was performed according to general procedure 2.4 from 42.4 mg pyridine building block **31** (172 µmol, 1.2 eq), 93.7 mg Cs_2CO_3 (288 µmol, 2.0 eq), 5.3 mg $PdCl_2$(dppf) (7.2 µmol, 5 mol%), 53.8 mg previously prepared intermediate (143 µmol, 1.0 eq) and 2 mL dry, degassed CH_3CN. The crude product was purified via column chromatography (5 g SiO_2, 1.5 x 20 cm, eluent: cyclohexane/EtOAc = 1/2).

Yield: 32 mg (53% over 2 steps), yellow oil, $C_{22}H_{24}N_sS$ [348.51 g/mol].

^{1}H NMR (300 MHz, $CDCl_3$): δ = 8.89 (s, 1H; H^{Ar}), 8.62 (d, $^4J_{H,H}$ = 4.5 Hz, 1H; H^{Ar}), 8.53 (s, 1H; H^{Ar}), 8.45 (s, 1H; H^{Ar}), 7.93 (d, $^3J_{H,H}$ = 7.7 Hz, 1H; H^{Ar}), 7.56-7.51 (m, 3H; H^{Ar}), 7.42-7.38 (m, 1H; H^{Ar}), 7.33 (d, $^3J_{H,H}$ = 7.8 Hz, 1H; H^{Ar}), 3.07-2.91 (m, 3H; CH_2 & CH), 2.61-2.57 (m, 2H; CH_2), 1.90 (s, 3H; CH_3), 1.30 (d, $^3J_{H,H}$ = 6.9 Hz, 6H; CH_3) ppm; **^{13}C NMR** (76 MHz, $CDCl_3$, APT): δ = 148.7 (C^{Ar}), 148.3 (C^{Ar}), 147.3 (C^{Ar}), 147.1 (C^{Ar}), 143.6 (C_q; C^{Ar}), 139.3 (C_q; C^{Ar}), 138.3 (C_q; C^{Ar}), 138.0 (C_q; C^{Ar}), 136.4 (C_q; C^{Ar}), 136.2 (C_q; C^{Ar}), 134.9 (C^{Ar}), 134.7 (C^{Ar}), 131.2 (C^{Ar}), 128.7 (C^{Ar}), 125.5 (C^{Ar}), 123.8 (C^{Ar}), 35.4 (CH_2), 33.2 (CH_2), 31.9 (CH), 23.8 (CH_3), 15.6 (CH_3) ppm; **TLC**: R_f = 0.20 (cyclohexane/EtOAc = 1/2, UV and CAM), **HRMS** (MALDI): calcd for [M^++H]: 349.1733; found: 349.1729.

5.4 Methyl 2-(2-(5-isopropylpyridin-3-yl)-5-(pyridin-3-yl)phenyl)acetate

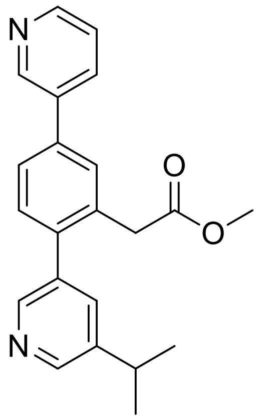

33a

The first coupling step was performed according to general procedure 2.3 from 215 mg pyridine building block **28** (1.05 mmol, 1.1 eq), 552 mg Ag_2CO_3 (2.0 mmol, 2.0 eq), 36.6 mg $PdCl_2$(dppf) (50.0 µmol, 5 mol%) and 424 mg **5** (1.0 mmol, 1.0 eq) in 5 mL abs., degassed MeCN. The crude product was purified via column chromatography (15 g SiO_2, 2 x 15 cm, eluent: cyclohexane/EtOAc = 6/1 → 4/1, R_f = 0.34 (cyclohexane/EtOAc = 3/1, UV)) to give 183 mg (49%) diaryl intermediate as a pale-yellow oil.

The second coupling was performed according to general procedure 2.4 from 86.4 mg pyridine building block **31** (350 µmol, 1.1 eq), 217 mg Cs_2CO_3 (667 µmol, 2.0 eq), 12.1 mg $PdCl_2$(dppf) (16.7 µmol, 5 mol%), 125 mg previously prepared intermediate (333 µmol, 1.0 eq) and 3 mL abs., degassed toluene. The crude product was purified via column chromatography (10 g SiO_2, 2 x 20 cm, eluent: cyclohexane/EtOAc = 1/1 → 1/3).

Yield: 64 mg (27% over 2 steps), pale-yellow oil, $C_{22}H_{22}N_2O_2$ [346.42 g/mol].

^{1}H NMR (300 MHz, $CDCl_3$): δ = 9.11 (s, 1H; H^{Ar}), 8.80 (s, 1H; H^{Ar}), 8.70 (m, 2H; H^{Ar}), 8.44 (d, ^3J (H,H) = 7.6 Hz, 1H; H^{Ar}), 8.23 (s, 1H; H^{Ar}), 7.95 – 7.83 (m, 1H; H^{Ar}), 7.78 – 7.63 (m, 2H; H^{Ar}), 7.48 (d, ^3J (H,H) = 7.6 Hz, 1H; H^{Ar}), 3.69 (s, 3H; CH_3), 3.62 (s, 2H; CH_2), 3.30 – 3.10 (m, 1H; CH), 1.40 (d, ^3J (H,H) = 6.9 Hz, 6H; CH_3); **^{13}C NMR** (76 MHz, $CDCl_3$): δ = 171.1 (C_q; C=O), 147.8 (C_q; C^{Ar}), 142.8 (C^{Ar}), 142.5 (C^{Ar}), 142.4 (C^{Ar}), 140.6 (C^{Ar}), 140.5 (C^{Ar}), 140.2 (C^{Ar}), 138.8 (C_q; C^{Ar}), 138.5 (C_q; C^{Ar}), 137.1 (C_q; C^{Ar}), 136.4 (C_q; C^{Ar}), 133.9 (C_q; C^{Ar}), 131.8 (C^{Ar}), 130.3 (C^{Ar}), 126.9 (C^{Ar}), 126.4 (C^{Ar}), 52.7 (CH_3), 38.7 (CH_2), 32.1 (CH), 23.3 (CH_3) ppm; **TLC**: R_f = 0.16 (cyclohexane/EtOAc = 1/2, UV and CAM), **HRMS** (EI): calcd for [M^+]: 346.1681; found: 346.1687.

5.5 Gly-Asp-Val

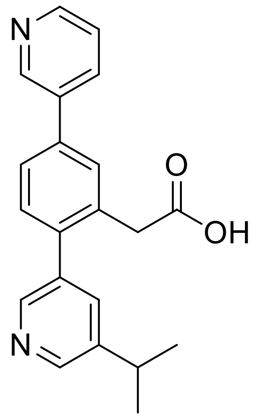

33

In a 10 mL round-bottom flask 34.6 mg compound **33a** (100 µmol, 1.0 eq) were dissolved in 2 mL THF/H_2O (2/1). 42 mg LiOH·H_2O (1.00 mmol, 10 eq) were added and the resulting heterogenic mixture was stirred at RT. After full conversion was detected via TLC 1.0 mL aq. HCl (1M) was added. The solution was extracted with DCM (3 x 10 mL). The organic layer was dried over Na_2SO_4, filtered and evaporated under reduced pressure. The crude product was purified via semi-preparative HPLC (MV_NucleodurC18_001HCOOH_10to100).

Yield: 25 mg (75%), colorless foam, $C_{21}H_{20}N_2O_2$ [332.40 g/mol].

^{1}H NMR (300 MHz, [D$_6$]DMSO): δ = 9.15 (s, 1H; H^{Ar}), 8.79 (m, 2H; H^{Ar}), 8.71 (d, ^3J (H,H) = 1.4 Hz, 1H; H^{Ar}), 8.53 (d, ^3J (H,H) = 8.0 Hz, 1H; H^{Ar}), 8.25 (s, 1H; H^{Ar}), 8.00 – 7.79 (m, 3H; H^{Ar}), 7.56 (d, ^3J (H,H) = 7.9 Hz, 1H; H^{Ar}), 3.71 (s, 2H; CH_2), 3.27 – 3.05 (m, 1H; CH), 1.30 (d, ^3J (H,H) = 6.9 Hz, 6H; CH_3) ppm; **^{13}C NMR** (76 MHz, [D$_6$]DMSO): δ = 172.4 (C_q; C=O), 145.7 (C_q; C^{Ar}), 145.3 (C^{Ar}), 144.3 (C^{Ar}), 142.1 (C^{Ar}), 142.1 (C^{Ar}), 140.1 (C^{Ar}), 138.3 (C^{Ar}), 137.5 (C_q; C^{Ar}), 137.1 (C_q; C^{Ar}), 136.2 (C_q; C^{Ar}), 136.0 (C_q; C^{Ar}), 134.4 (C_q; C^{Ar}), 131.2 (C^{Ar}), 130.1 (C^{Ar}), 126.0 (C^{Ar}), 125.5 (C^{Ar}), 40.4 (CH; overlaps with solvent signal), 31.0 (CH), 23.0 (CH_3) ppm; **HRMS** (EI): calcd for [M^+]: 332.1525; found: 332.1512.

5.6 3-(2-(4-((tert-Butyldiphenylsilyl)oxy)benzyl)-4-(pyridin-3-yl)phenyl)-5-isopropylpyridine

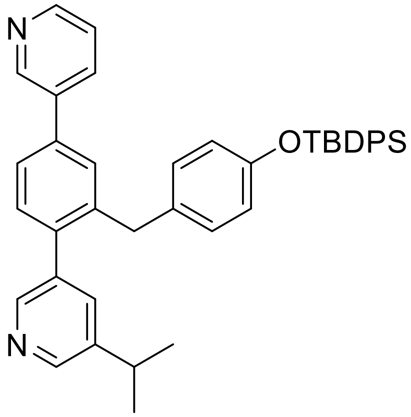

34a

The first coupling was performed according to general procedure 2.3 from 103 mg pyridine building block **28** (550 µmol, 1.1 eq), 276 mg Ag_2CO_3 (1.0 mmol, 2.0 eq), 18.9 mg $PdCl_2$(dppf) (25.0 µmol, 5 mol%) and 348 mg core building block **20** (500 µmol, 1.0 eq) in 5 mL abs., degassed toluene. The reaction was stopped after 3 h by filtration through a short pad of SiO_2 to avoid hydrolysis of the triflate group. The crude product was purified via column chromatography (15 g SiO_2, 2 x 15 cm, eluent: cyclohexane/EtOAc = 2/1, R_f = 0.36, UV) to give 150 mg (46%) diaryl intermediate as a brown oil.

The second coupling was performed according to general procedure 2.4 from 60 mg pyridine building block **31** (240 µmol, 1.1 eq), 149 mg Cs_2CO_3 (460 µmol, 2.0 eq), 11.7 mg $PdCl_2$(dppf) (11.5 µmol, 5 mol%), 150 mg previously prepared intermediate (230 µmol, 1.0 eq) and 3 mL abs., degassed toluene. The crude product was purified via column chromatography (10 g SiO_2, 2 x 20 cm, eluent: cyclohexane/EtOAc = 2/1 → 1/1). The product was used in the following step without further purification.

Yield: 108 mg (35% crude yield over two steps), colorless oil, $C_{42}H_{42}N_2OSi$ [618.90 g/mol].

5.7 Gly-Tyr-Val

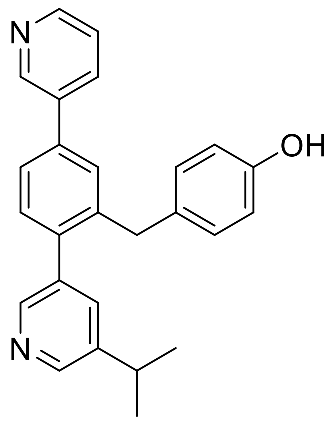

34

In a 10 mL round-bottom flask 48 mg compound **34a** (77.6 µmol, 1.0 eq) were dissolved in 3 mL THF. 64 mg TBAF·3H_2O (200 µmol, 2.6 eq) were added and the resulting yellow solution was stirred at RT for 1 h. After full conversion was detected via TLC, 10 mL H_2O were added. The reaction mixture was extracted with DCM (3 x 10 mL). The combined organic layers were dried over Na_2SO_4, filtered and evaporated under reduced pressure. The crude product was purified via column chromatography (10 g SiO_2, 1.2 x 22 cm, eluent: EtOAc).

Yield: 18 mg (60%), colorless oil, $C_{26}H_{24}N_2O$ [380.49 g/mol].

^{1}H NMR (300 MHz, $CDCl_3$): δ = 8.85 (d, ^4J (H,H) = 1.6 Hz, 1H; H^{Ar}), 8.59 (dd, ^3J (H,H) = 4.8 Hz, ^4J (H,H) = 1.3 Hz, 1H; H^{Ar}), 8.45 (d, ^4J (H,H) = 1.8 Hz, 1H; H^{Ar}), 8.34 (d, ^4J (H,H) = 1.8 Hz, 1H; H^{Ar}), 7.93 (d, ^3J (H,H) = 7.9 Hz, 1H; H^{Ar}), 7.52 – 7.50 (m, 2H; H^{Ar}), 7.45 – 7.38 (m, 2H; H^{Ar}), 7.34 (d, ^3J (H,H) = 8.4 Hz, 1H; H^{Ar}), 6.80 – 6.70 (m, 4H; H^{Ar}), 3.91 (s, 2H; CH_2), 2.97 – 2.88 (m, 1H; CH), 1.25 (d, ^3J (H,H) = 6.9 Hz, 6H; CH_3) ppm; **^{13}C NMR** (76 MHz, $CDCl_3$): δ = 155.6 (C_q; C^{Ar}), 148.1 (C^{Ar}), 147.8 (C^{Ar}), 146.6 (C^{Ar}), 146.5 (C^{Ar}), 143.6 (C_q; C^{Ar}), 140.2 (C_q; C^{Ar}), 138.4 (C_q; C^{Ar}), 137.5 (C_q; C^{Ar}), 136.7 (C_q; C^{Ar}), 136.4 (C_q; C^{Ar}), 135.1 (C^{Ar}), 134.8 (C^{Ar}), 131.2 (C_q; C^{Ar}), 131.1 (C^{Ar}), 129.6 (C^{Ar}), 129.4 (C^{Ar}), 125.2 (C^{Ar}), 123.9 (C^{Ar}), 115.6 (C^{Ar}), 38.6 (CH_2), 31.7 (CH), 23.6 (CH_3) ppm; **TLC**: R_f = 0.63 (MeOH/EtOAc = 1/10, UV and CAM); **HRMS** (EI): calcd for [M^++H]: 381.1967; found: 381.1960.

5.8 Gly-Trp-Val

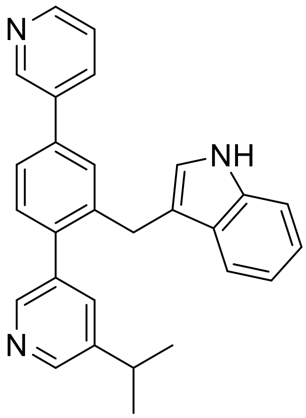

35

The first coupling was performed according to general procedure 2.3 from 173 mg pyridine building block **28** (840 µmol, 1.1 eq), 441 mg Ag_2CO_3 (1.6 mmol, 2.0 eq), 29.4 mg $PdCl_2$(dppf) (40.0 µmol, 5 mol%) and 360 mg core building block **22** (800 µmol, 1.0 eq) in 5 mL abs., degassed DMF. The crude product was purified via column chromatography (15 g SiO_2, 2 x 15 cm, eluent: cyclohexane/EtOAc = 2/1 → 1/1, R_f = 0.13, UV) to give 142 mg (44%) diaryl intermediate as a yellow solid.

The second coupling was performed according to general procedure 2.4 from 84.0 mg pyridine building block **31** (340 µmol, 1.1 eq), 208 mg Cs_2CO_3 (640 µmol, 2.0 eq), 11.7 mg $PdCl_2$(dppf) (16.0 µmol, 5 mol%), 140 mg previously prepared intermediate (320 µmol, 1.0 eq) and 3 mL abs., degassed CH_3CN. The crude product was purified via column chromatography (10 g SiO_2, 2 x 20 cm, eluent: cyclohexane/EtOAc = 1/1 → 1/2).

Yield: 75 mg (24% over two steps), colorless oil, $C_{28}H_{25}N_3$ [403.52 g/mol].

^{1}H NMR (300 MHz, $CDCl_3$): δ = 9.02 (s, 1H; H^{Ar}), 8.86 (s, 1H; NH), 8.71 (d, ^3J (H,H) = 4.5 Hz, 1H; H^{Ar}), 8.39 (s, 2H; H^{Ar}), 8.32 (d, ^3J (H,H) = 7.4 Hz, 1H; H^{Ar}), 7.74 (m, 3H; H^{Ar}), 7.60 (d, ^3J (H,H) = 7.0 Hz, 1H; H^{Ar}), 7.37 – 7.22 (m, 2H; H^{Ar}), 7.16 – 7.00 (m, 2H; H^{Ar}), 6.93 (t, ^3J (H,H) = 7.4 Hz, 1H; H^{Ar}), 6.62 (s, 1H; H^{Ar}), 4.04 (s, 2H; CH_2), 2.91 – 2.74 (m, 1H; CH), 1.09 (d, ^3J (H,H) = 6.9 Hz, 6H; CH_3) ppm; **^{13}C NMR** (76 MHz, $CDCl_3$): δ = 147.3 (C_q; C^{Ar}), 143.3 (C^{Ar}), 142.9 (C^{Ar}), 141.7 (C^{Ar}), 140.8 (C_q; C^{Ar}), 139.8 (C^{Ar}), 139.4 (C_q; C^{Ar}), 139.4 (C^{Ar}), 138.9 (C^{Ar}), 138.3 (C_q; C^{Ar}), 136.4 (C_q; C^{Ar}), 136.4 (C_q; C^{Ar}), 131.5 (C^{Ar}), 129.7 (C^{Ar}), 126.5 (C_q; C^{Ar}), 126.0 (C^{Ar}), 125.8 (C^{Ar}), 123.2 (C^{Ar}), 122.5 (C^{Ar}), 119.7 (C^{Ar}), 118.3 (C^{Ar}), 114.2 (C_q; C^{Ar}), 111.8 (C^{Ar}), 31.7

(CH_2), 30.3 (CH), 22.9 (CH_3) ppm; **TLC**: R_f = 0.39 (cyclohexane/EtOAc = 1/2, UV and CAM), **HRMS** (EI): calcd for [M^+]: 403.2048; found: 403.2056.

6 Literature:

[1] W. G. Kofron, L. M. Baclawski, *J. Org. Chem.* **1976**, *41*, 1879–1880.

[2] M. Trobe, R. Breinbauer, *Monatsh. Chem.* **2016**, *147*, 509–521.

[3] M. Peters, M. Trobe, H. Tan, R. Kleineweischede, R. Breinbauer, *Chem. - Eur. J.* **2013**, *19*, 2442–2449.

[4] M. Hanif, I. Khan, N. H. Rama, S. Noreen, M. I. Choudhary, P. G. Jones, M. Iqbal, *Med. Chem. Res.* **2012**, *21*, 3885–3896.

[5] O. Muñoz-Muñiz, E. Juaristi, *Tetrahedron* **2003**, *59*, 4223–4229.

[6] Z. Huang, X. Yang, F. Yang, T. Lu, Q. Zhou, *Org. Lett.* **2017**, *19*, 3524–3527.

[7] M. Schlosser, *Chem. Ber.* **1964**, *97*, 3219–3233.

[8] C. Quinet, L. Sampoux, I. E. Markó, *Eur. J. Org. Chem.* **2009**, *2009*, 1806–1811.

[9] T. Minami, I. Niki, T. Agawa, *Phosphorus, Sulfur Relat. Elem.* **1977**, *3*, 55–59.

[10] Z. Duchoslavová, R. Sivkova, V. Hanková, J. Sedláček, J. Svoboda, J. Vohlídal, J. Zedník, *Macromol. Chem. Phys.* **2011**, *212*, 1802–1814.

[11] G. Casiraghi, G. Casnati, G. Puglia, G. Sartori, *Synthesis* **1980**, *1980*, 124–125.

[12] M. E. B. Smith, R. M. Gunn, E. Rosivatz, L. H. Mak, R. Woscholski, H. C. Hailes, *Bioorg. Med. Chem.* **2010**, *18*, 4917–4927.

[13] S. G. Davies, D. Pyatt, C. Thomson, *J. Organomet. Chem.* **1990**, *387*, 381–390.

7 NMR

Methyl 2-(2-methoxyphenyl)acetate (**2a**)

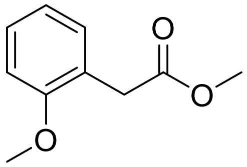

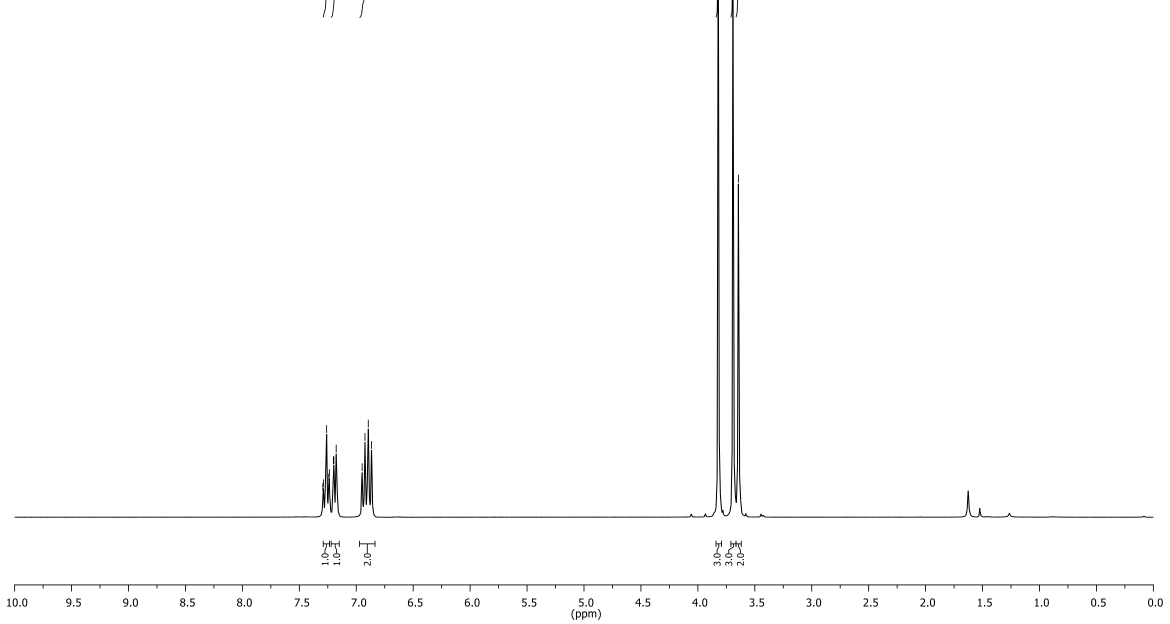

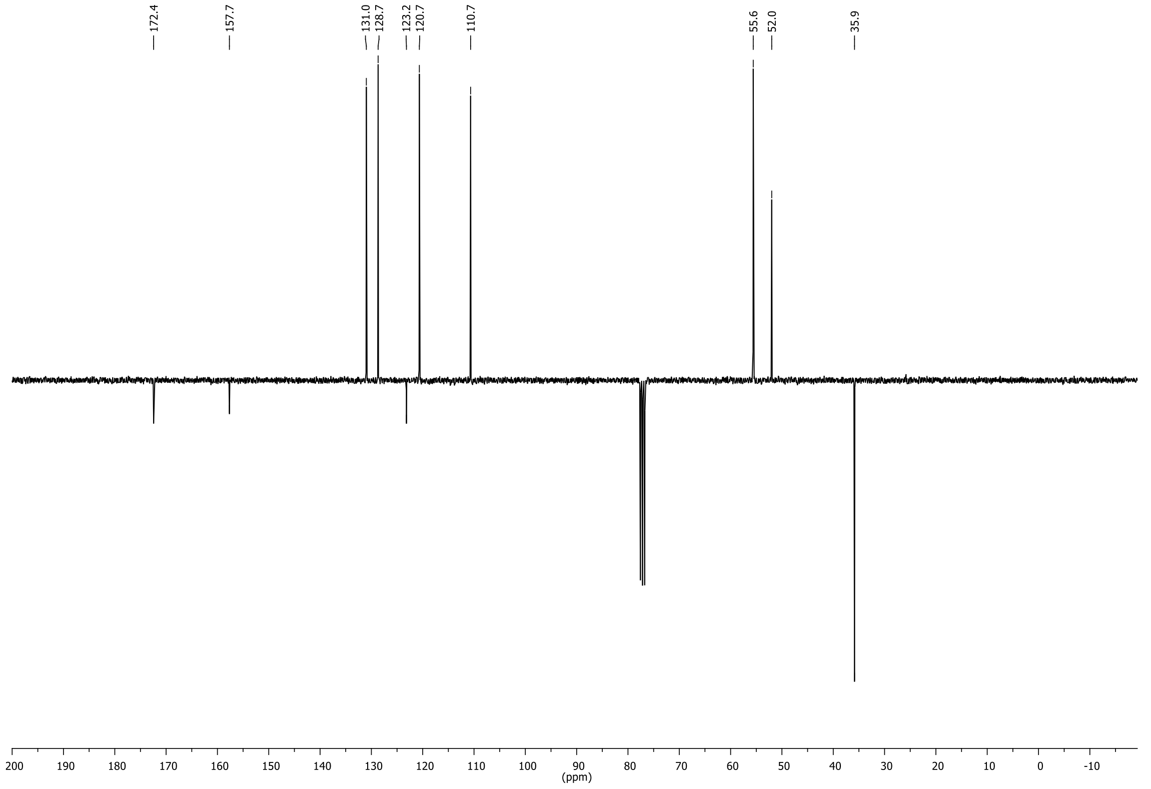

Methyl 2-(5-iodo-2-methoxyphenyl)acetate (**2**)

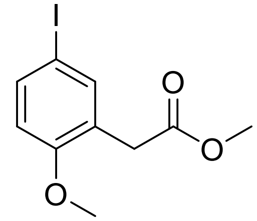

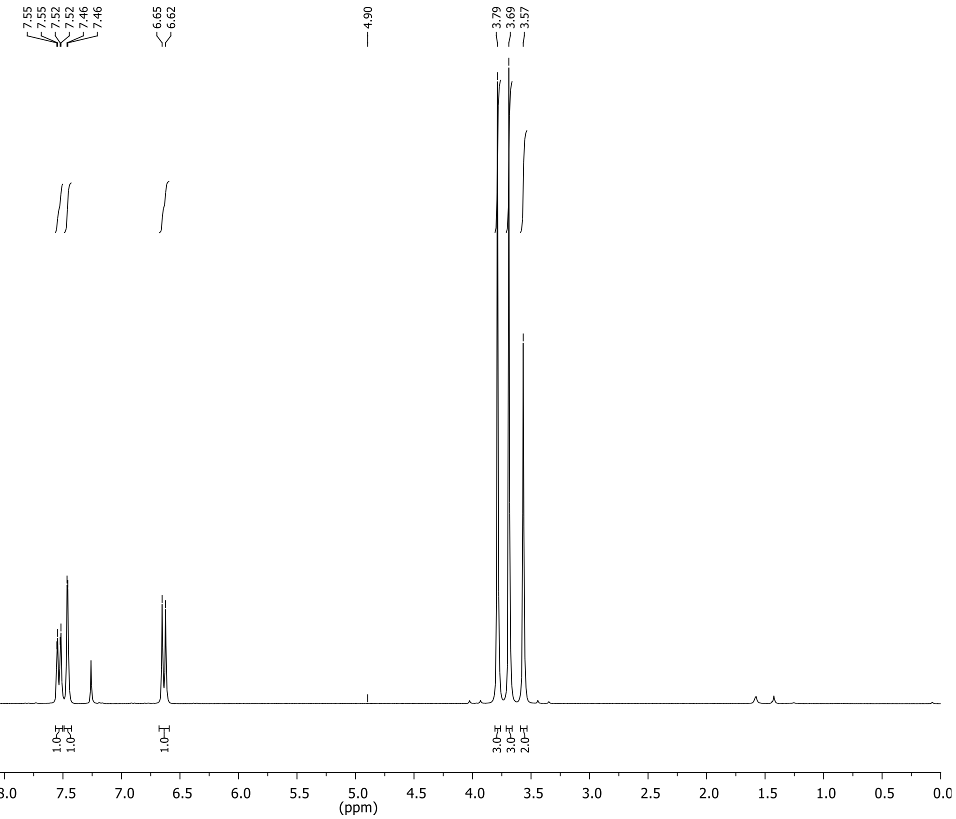

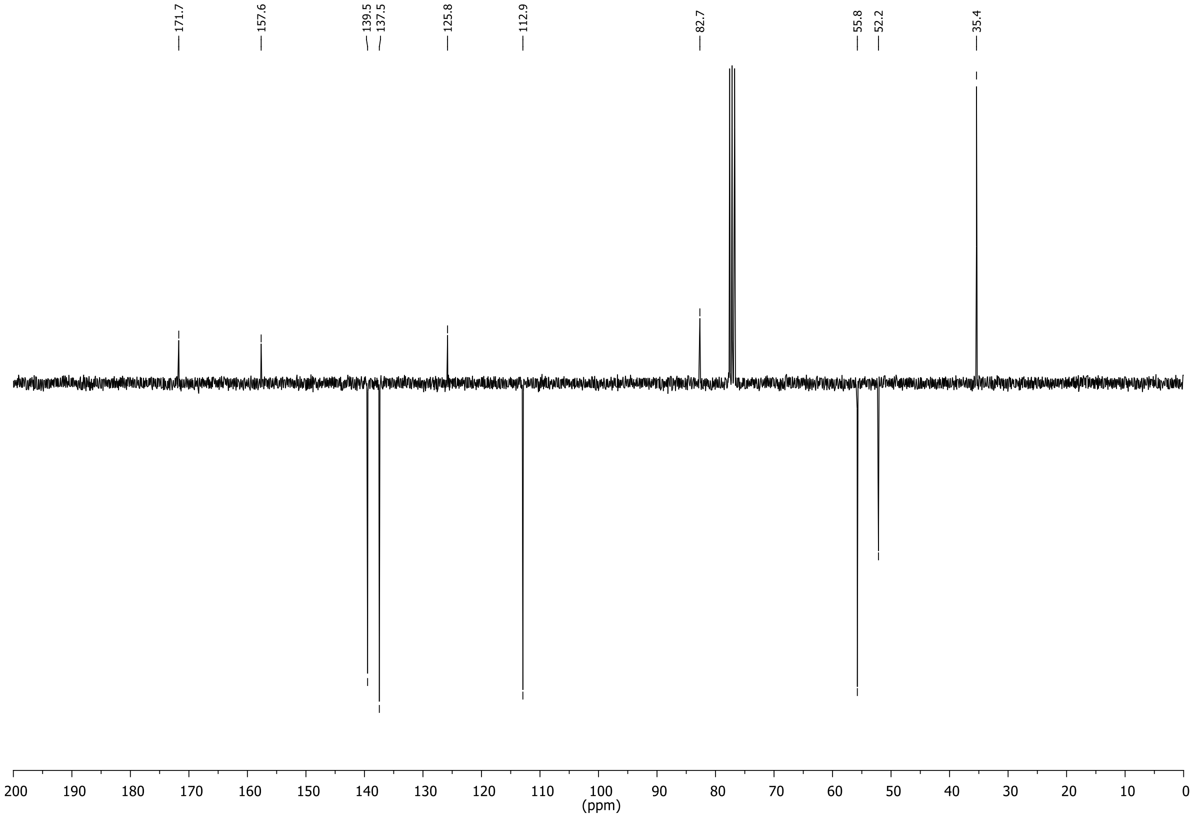

Benzofuran-2(3H)-one (**4a**)

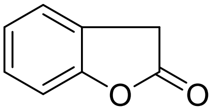

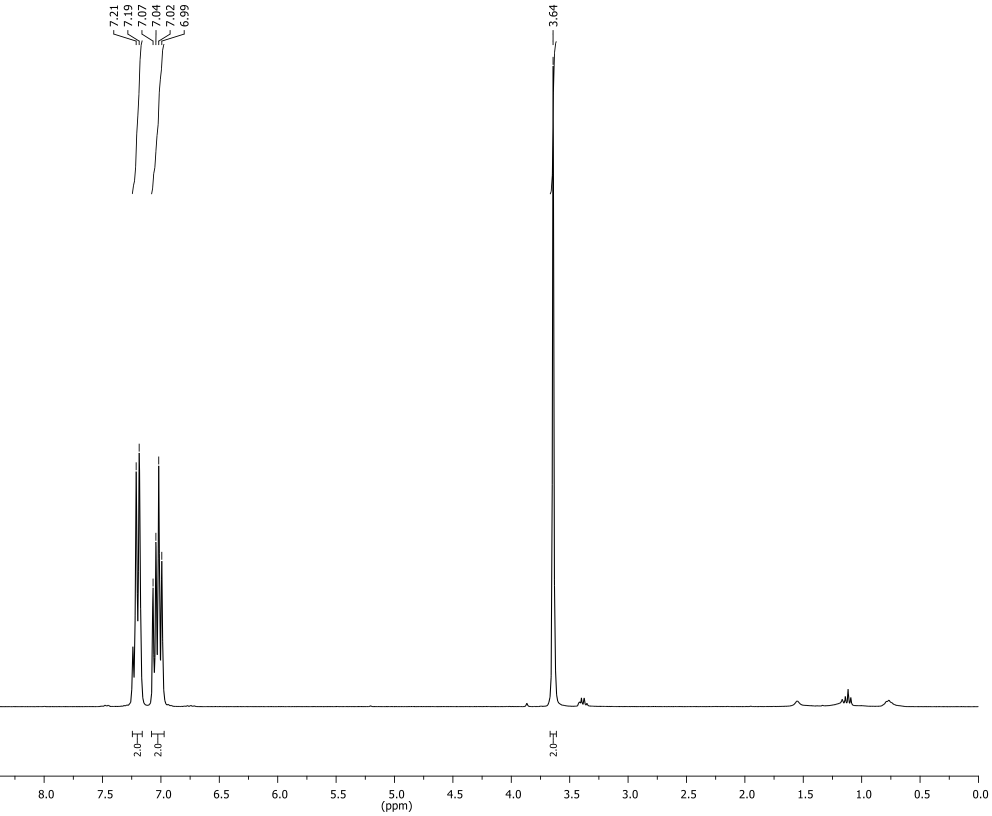

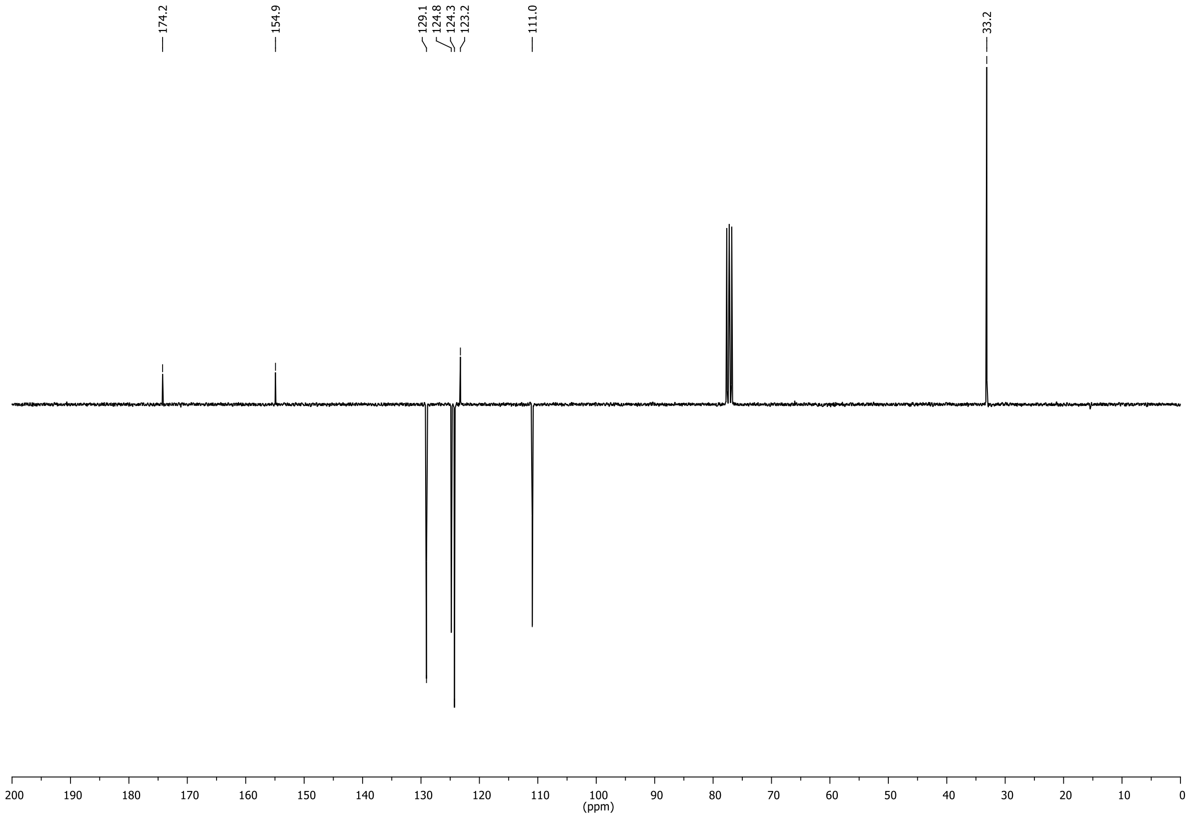

5-Iodobenzofuran-2(3H)-one (**4**)

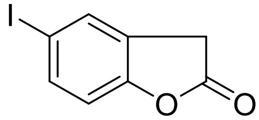

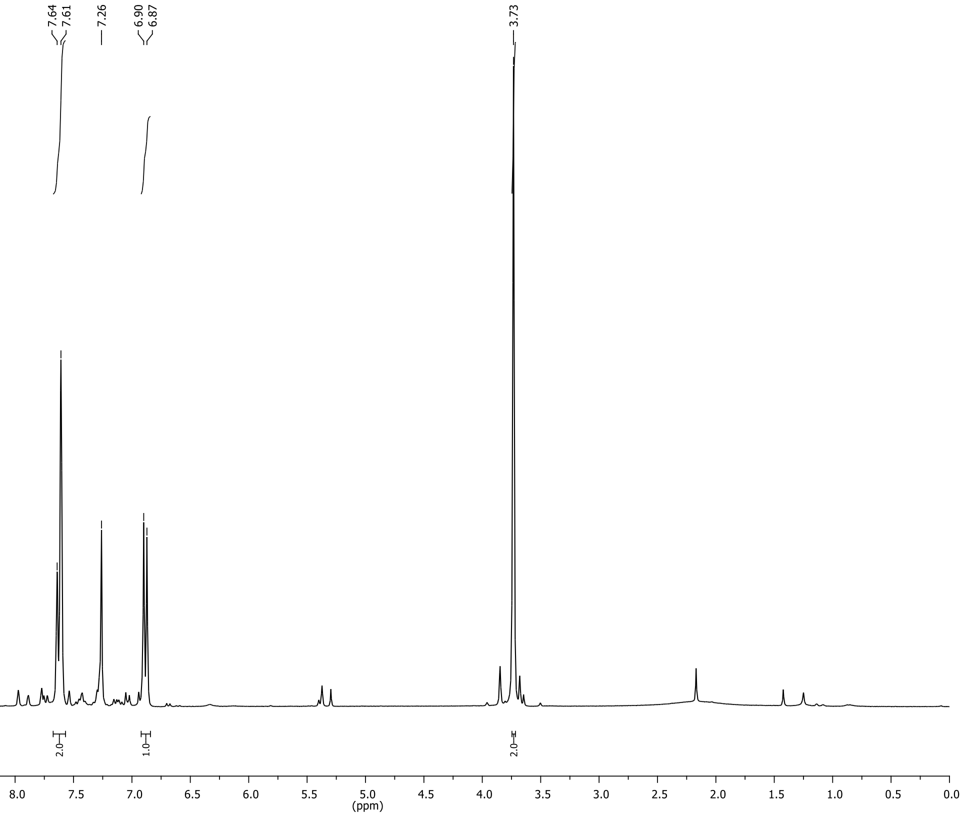

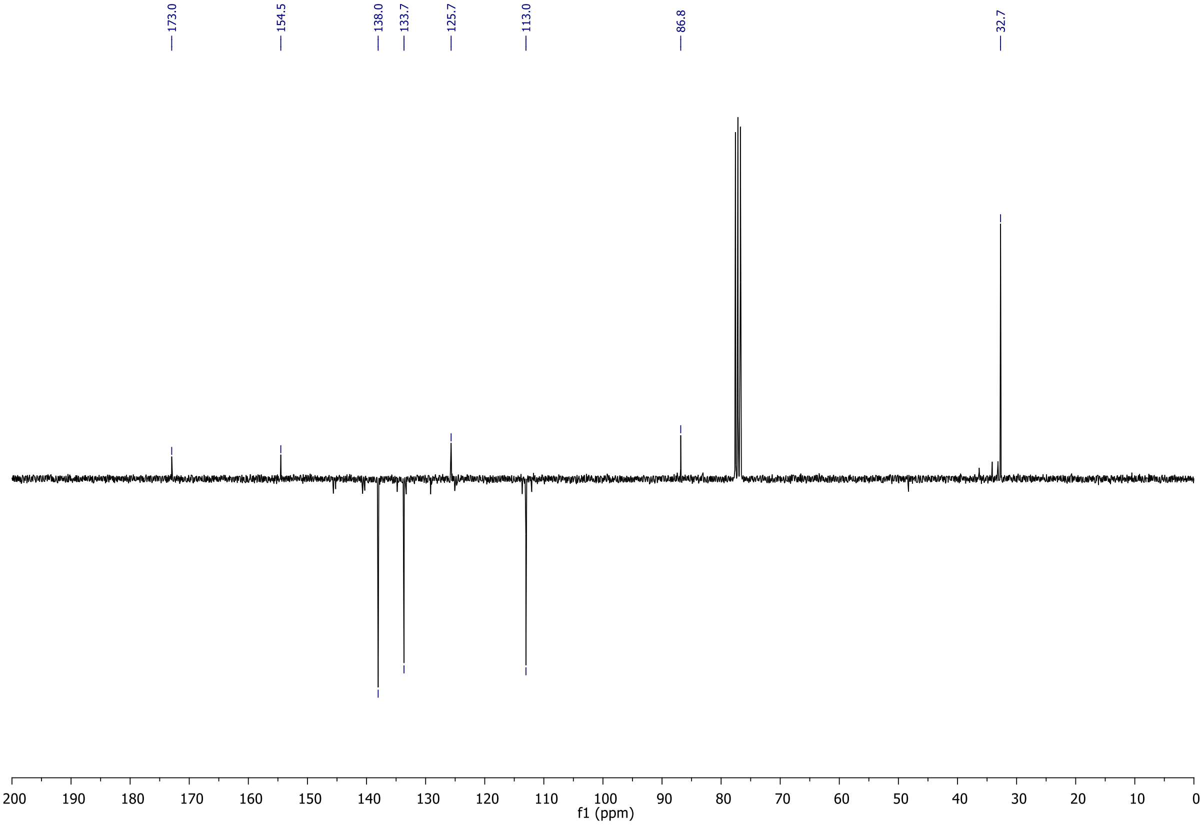

2-(2-Hydroxy-5-iodophenyl)acetic acid (**3a**)

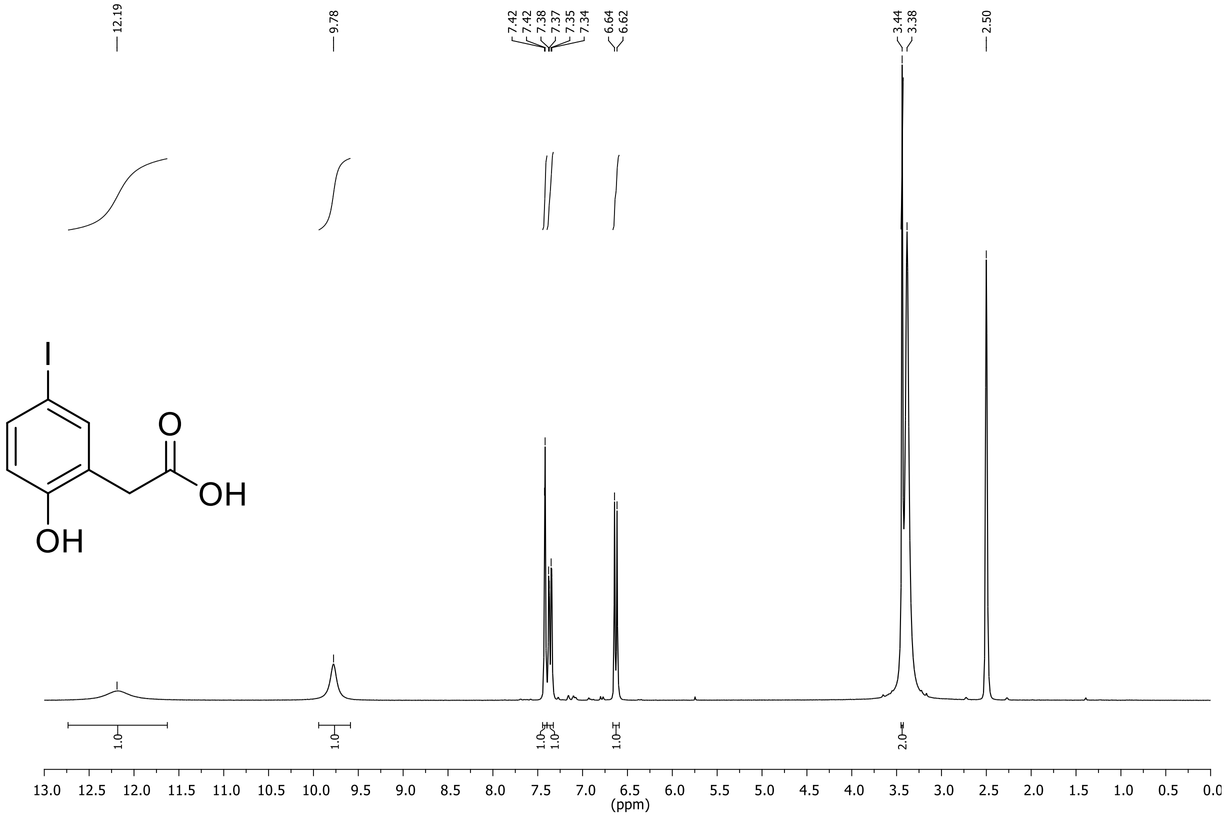

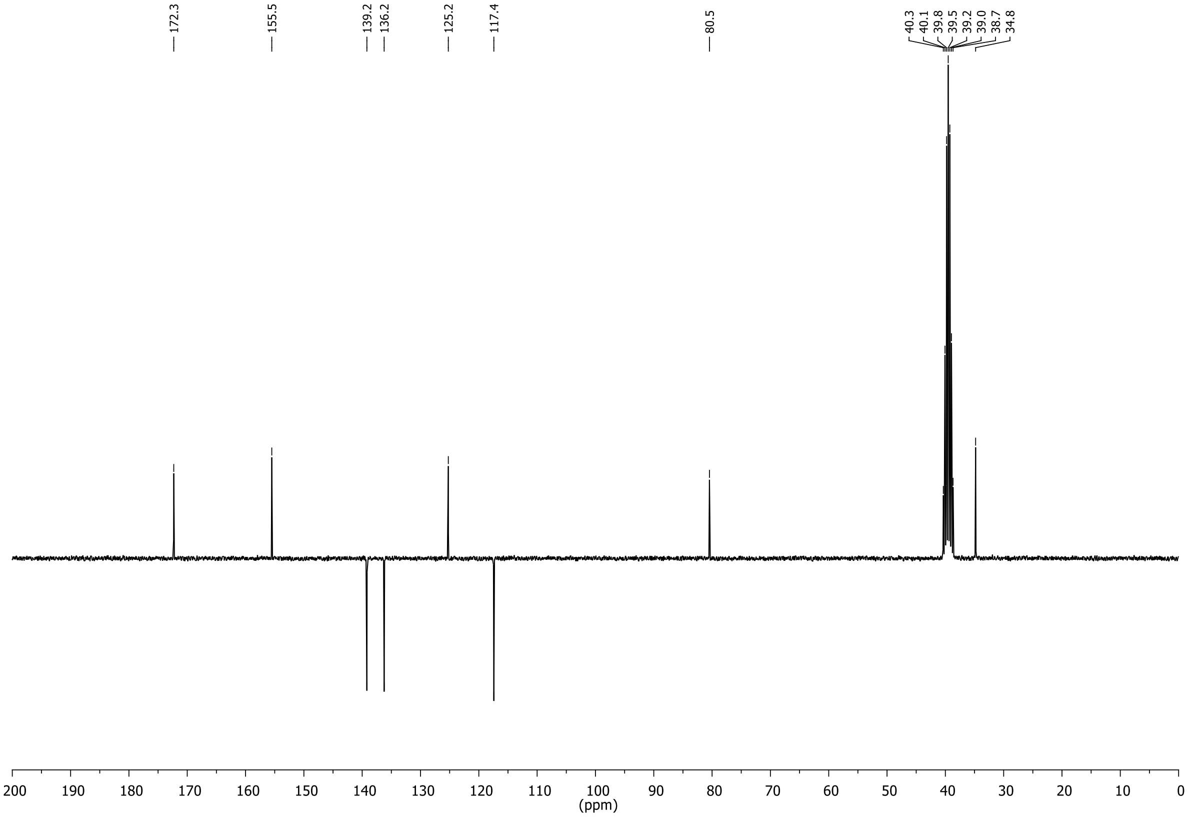

Methyl 2-(2-hydroxy-5-iodophenyl)acetate (**3**)

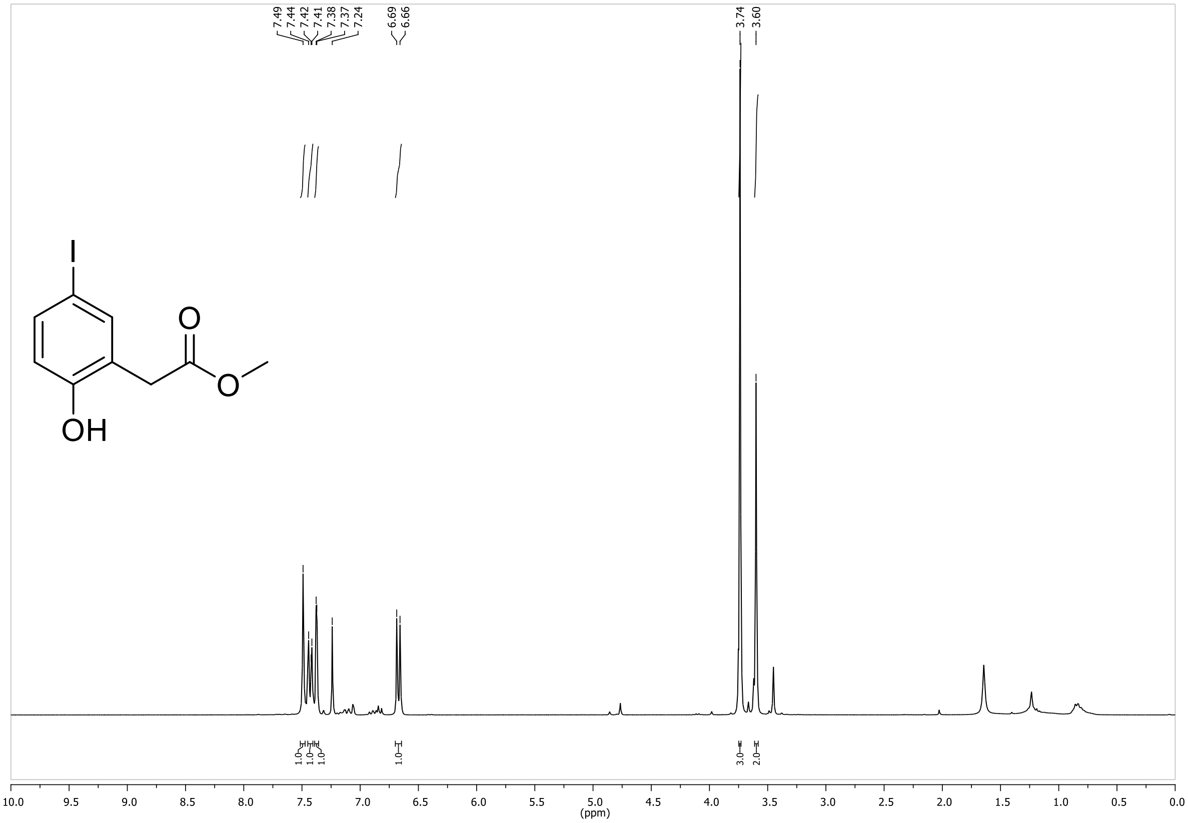

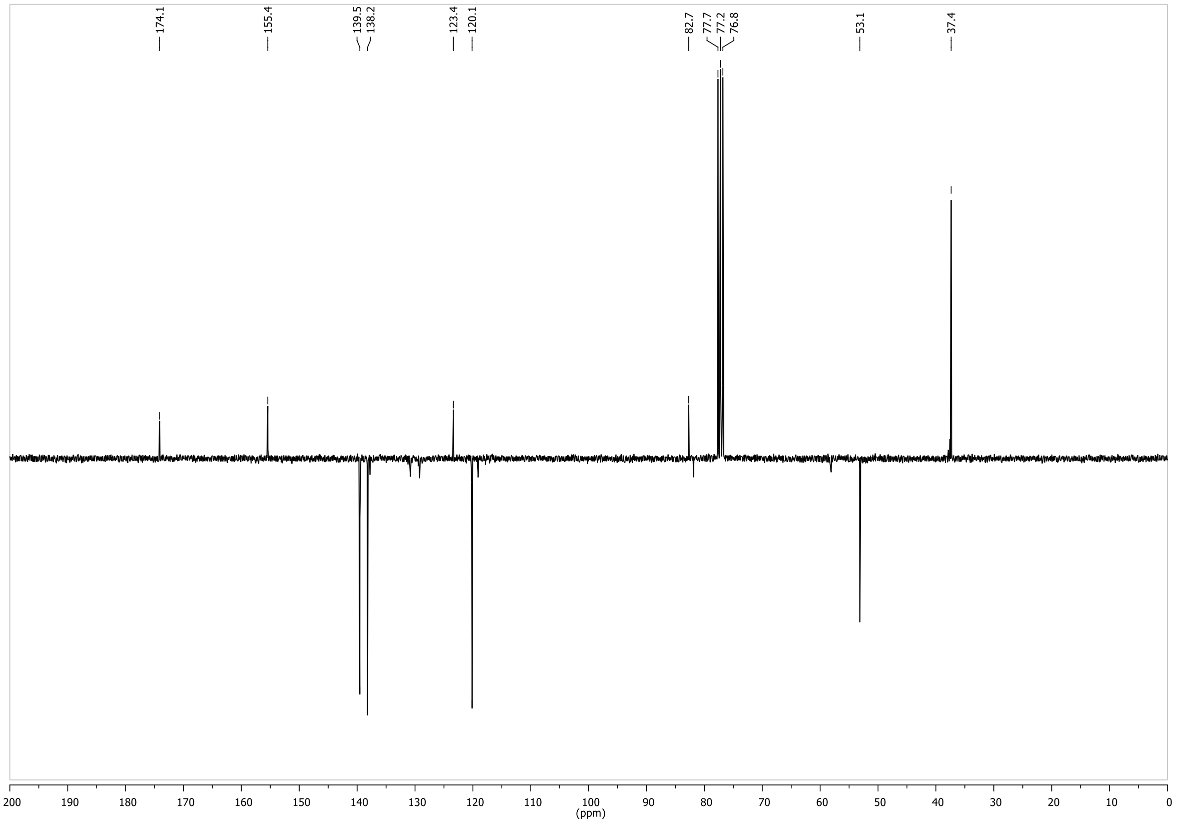

Methyl 2-(5-iodo-2-(((trifluoromethyl)sulfonyl)oxy)phenyl)acetate (**5**)

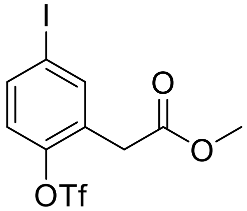

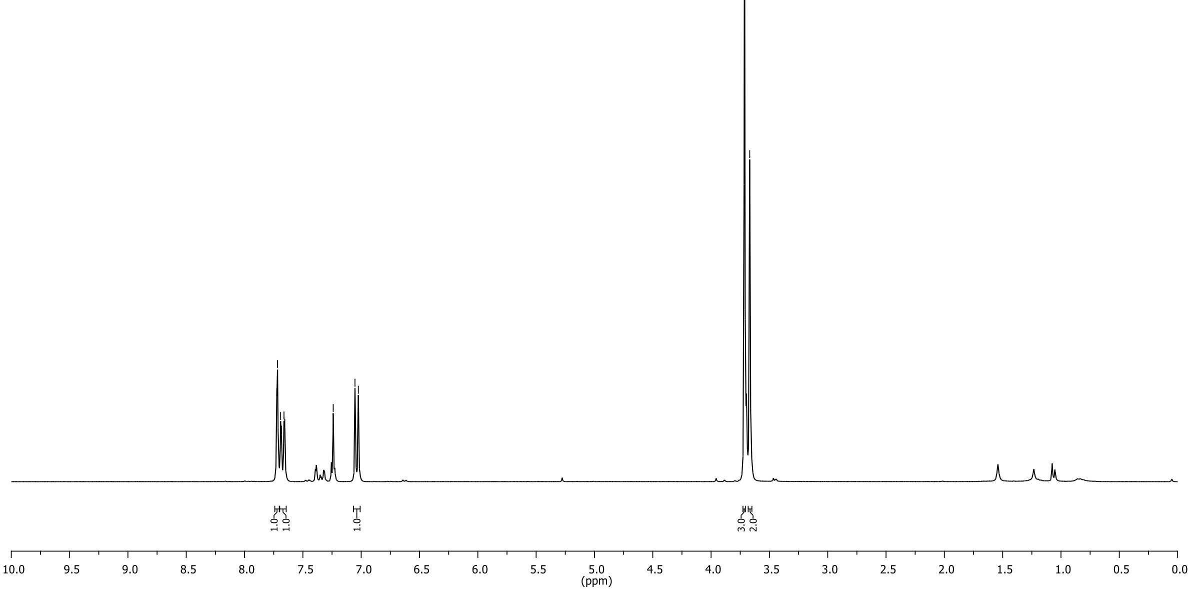

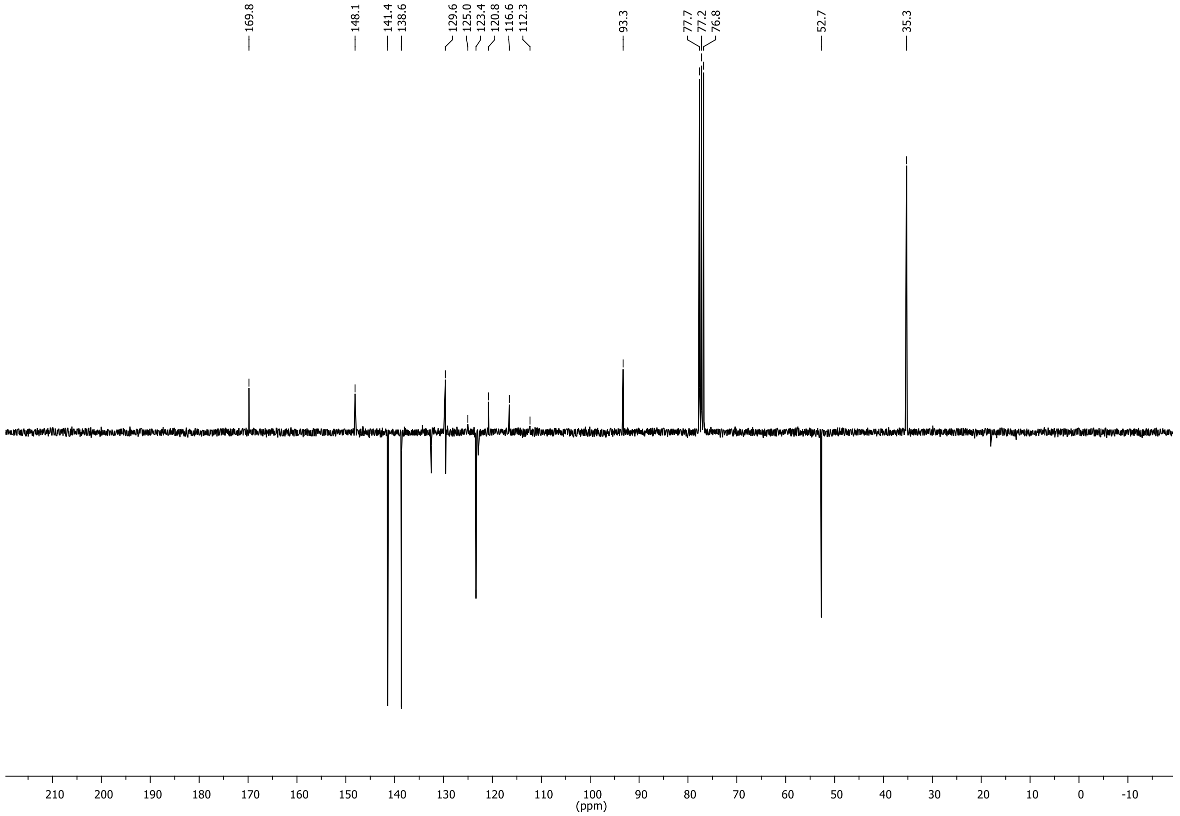

2-(5-Iodo-2-methoxyphenyl)acetonitrile (**7**)

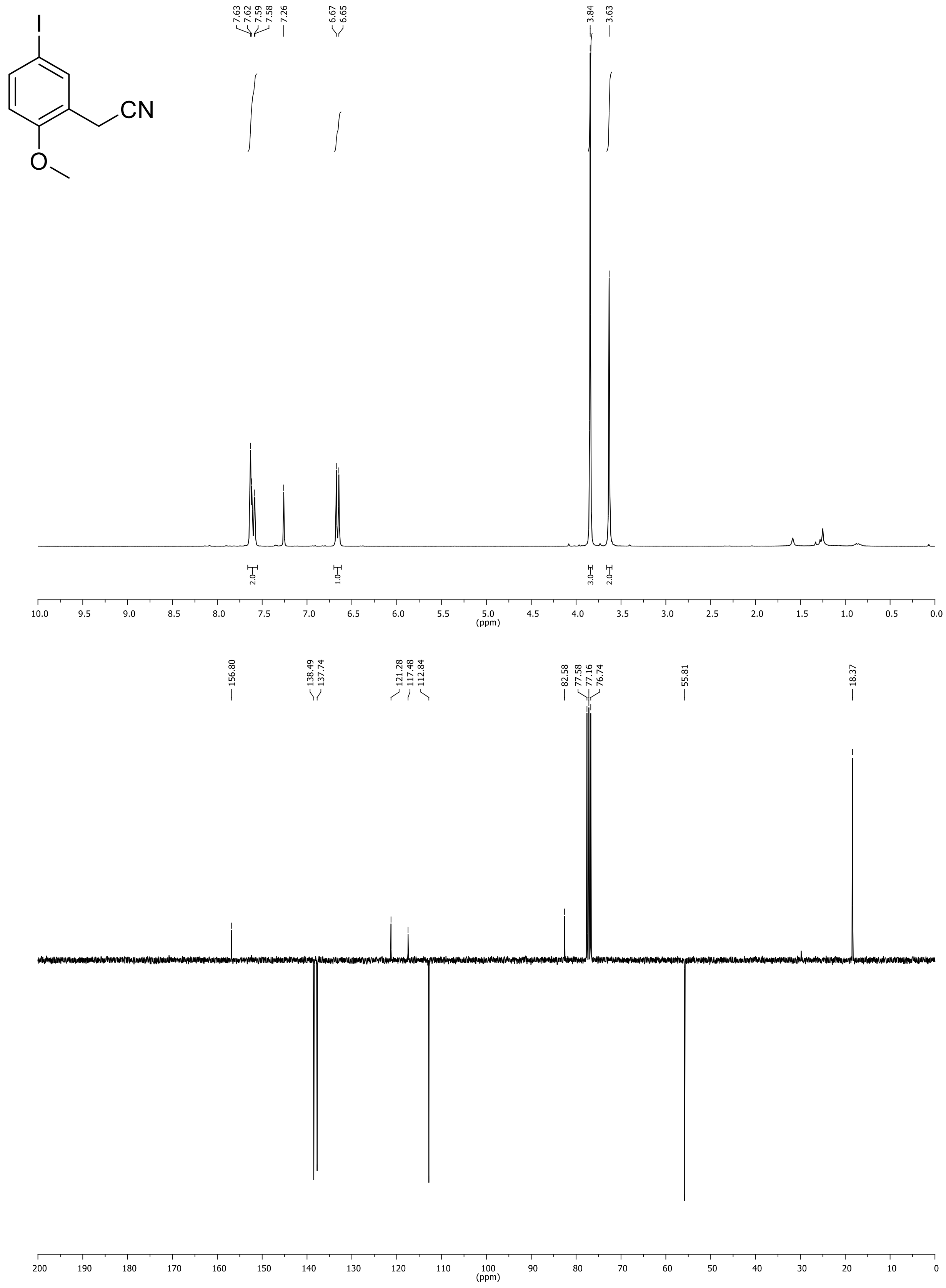

2-(2-Hydroxy-5-iodophenyl)acetonitrile (**8a**)

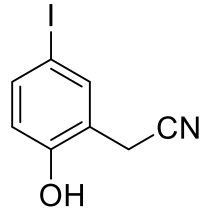

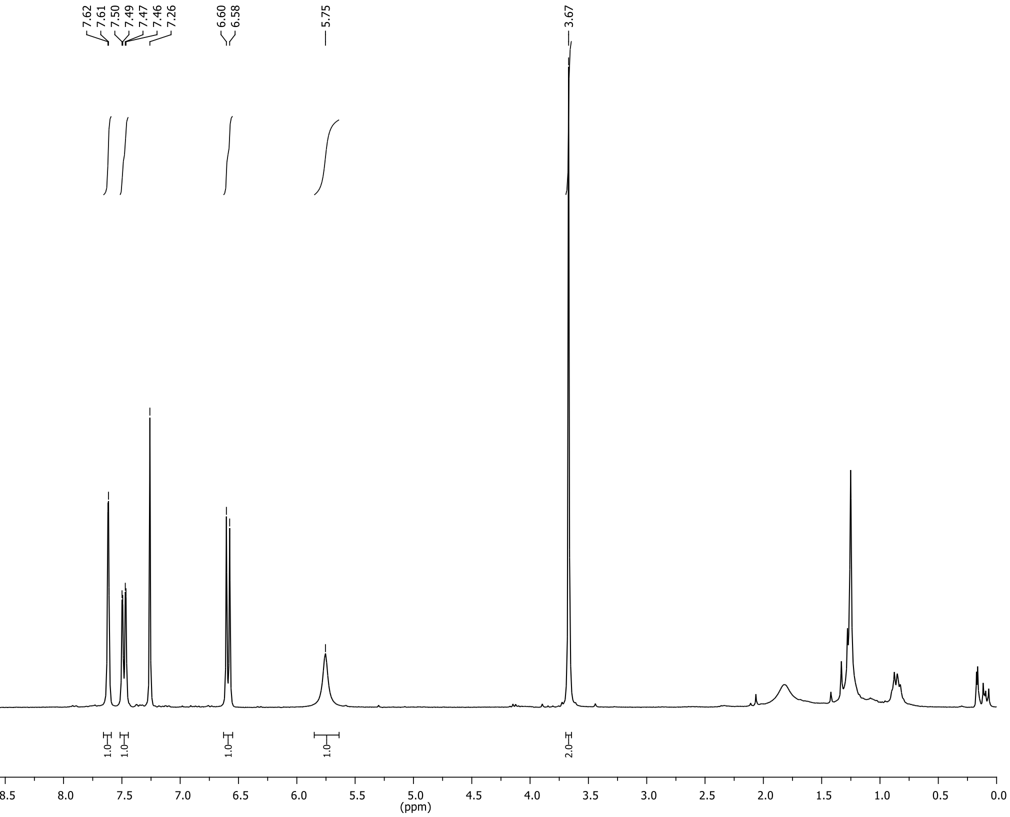

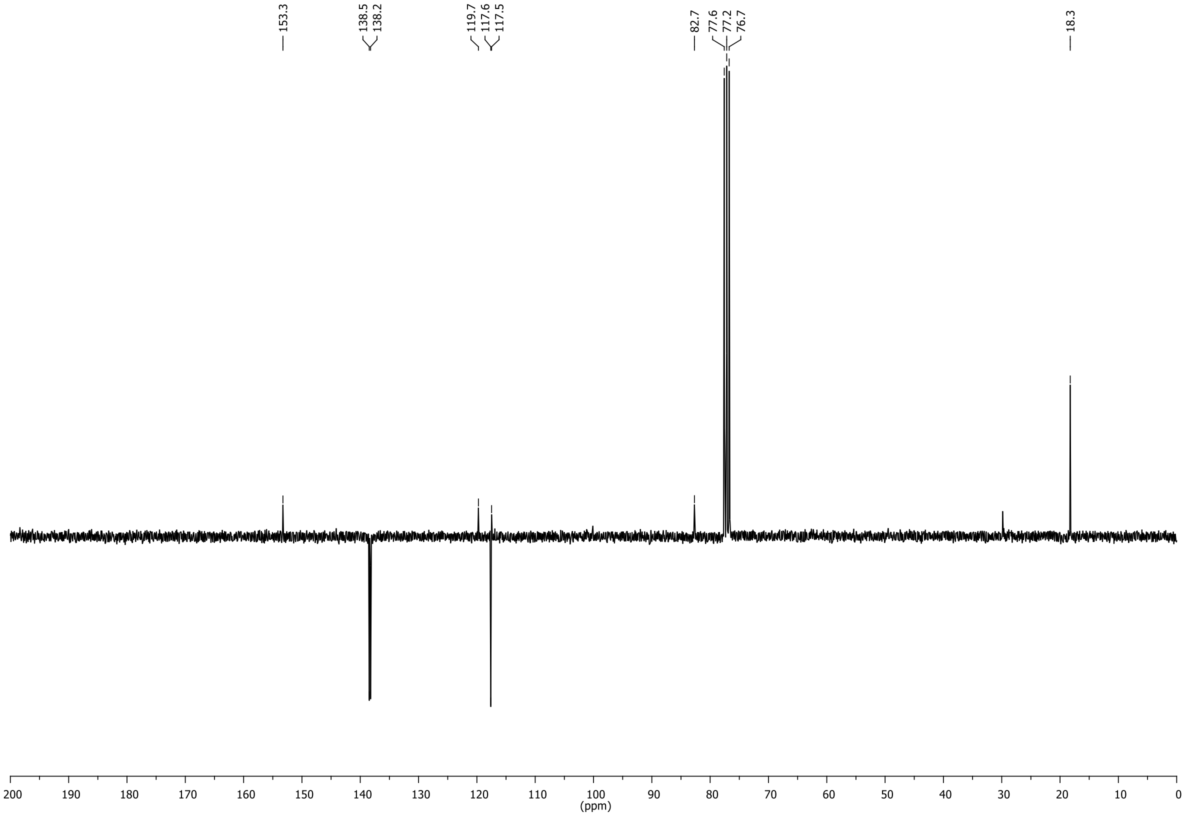

2-(Cyanomethyl)-4-iodophenyl trifluoromethanesulfonate (**8**)

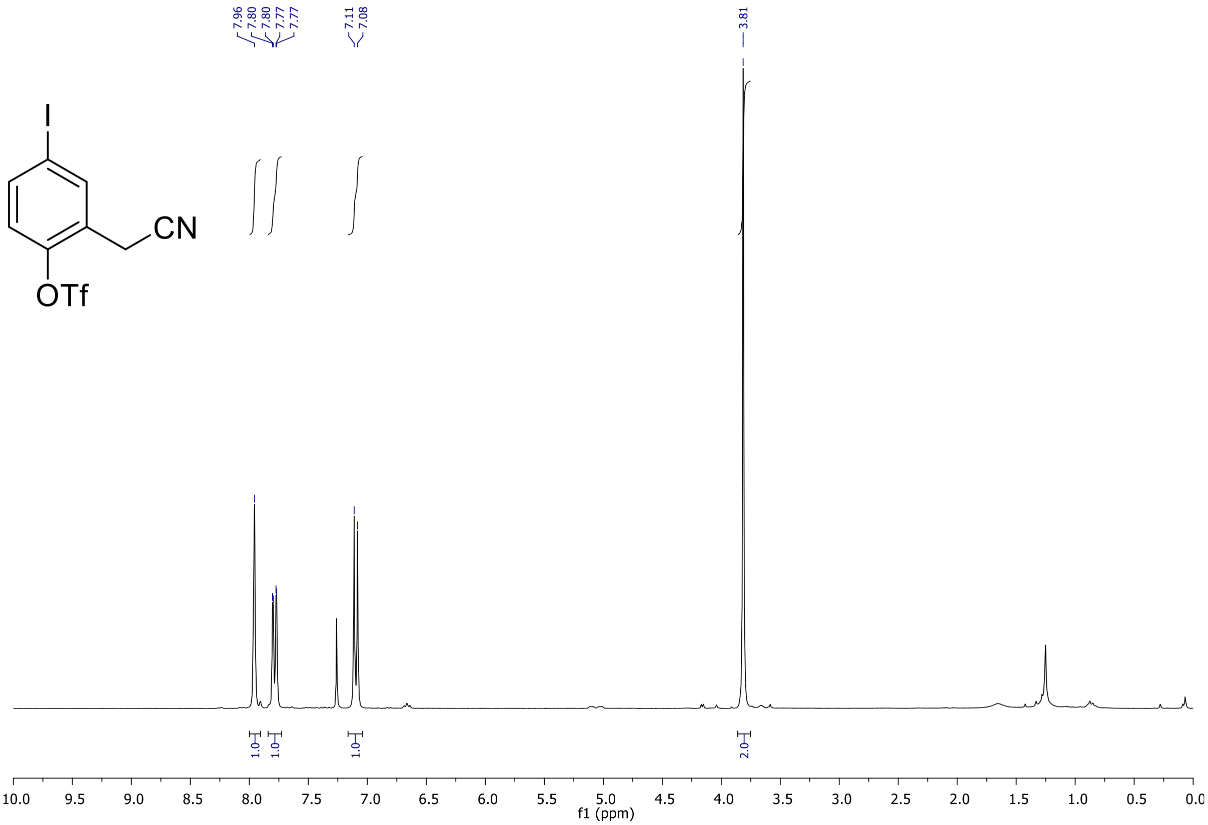

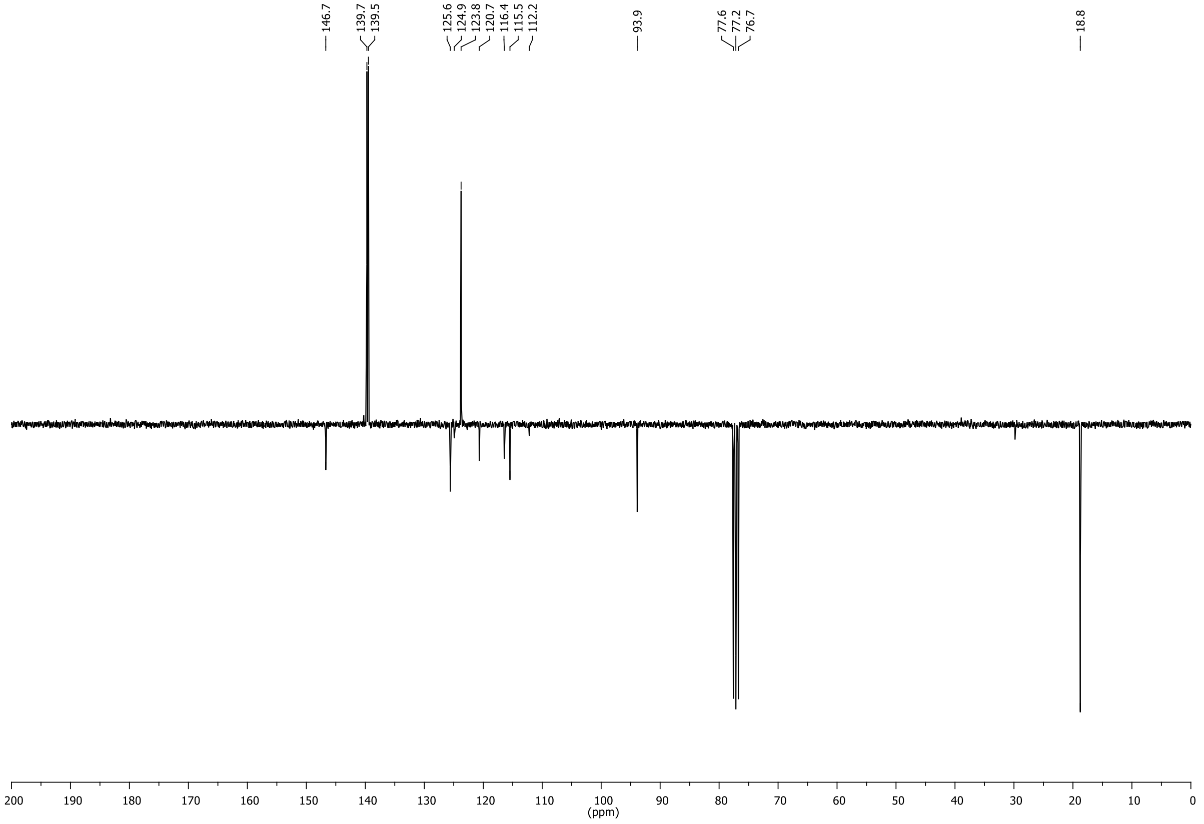

((Methylthio)methyl)triphenylphosphonium chloride (**10**)

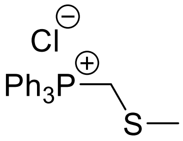

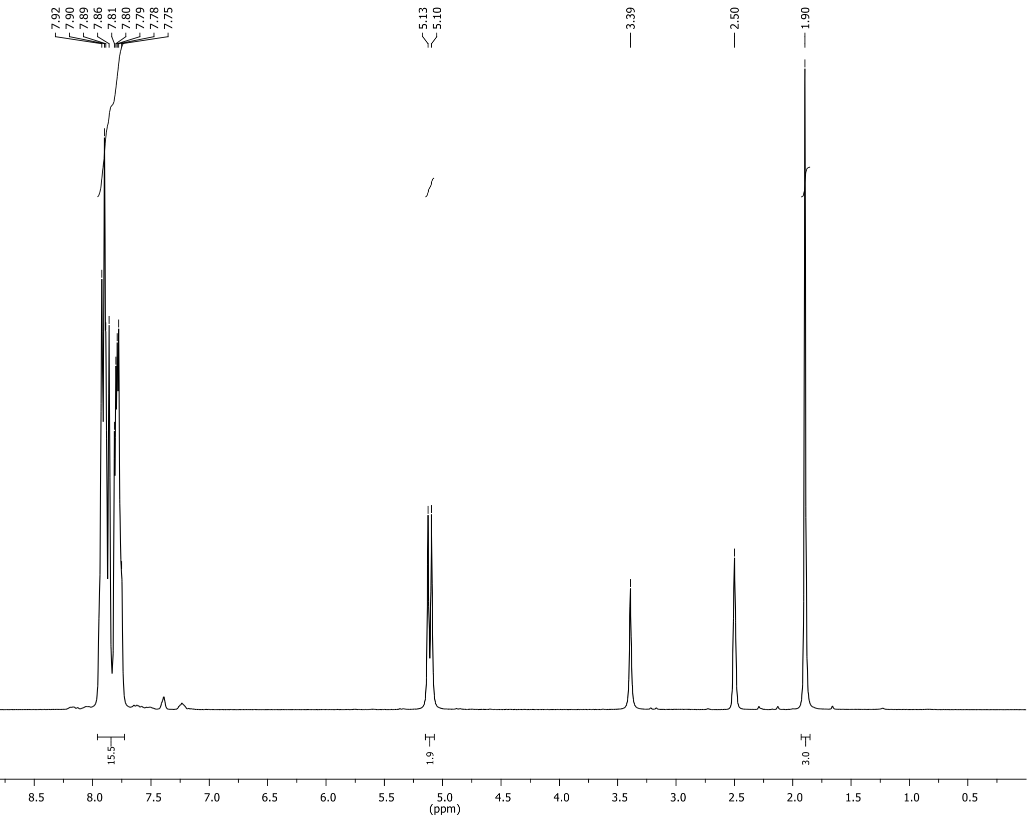

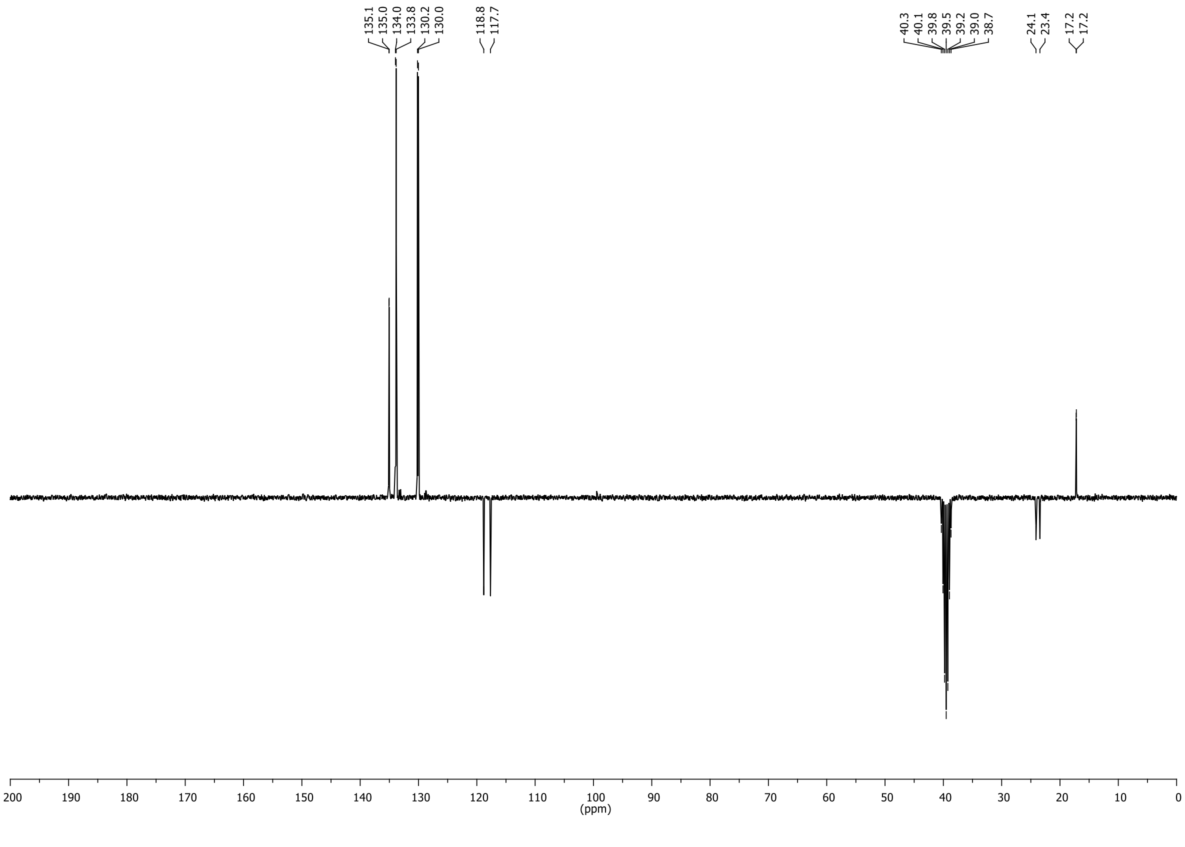

2-(2-(Methylthio)vinyl)phenol (**11a**)

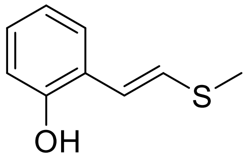

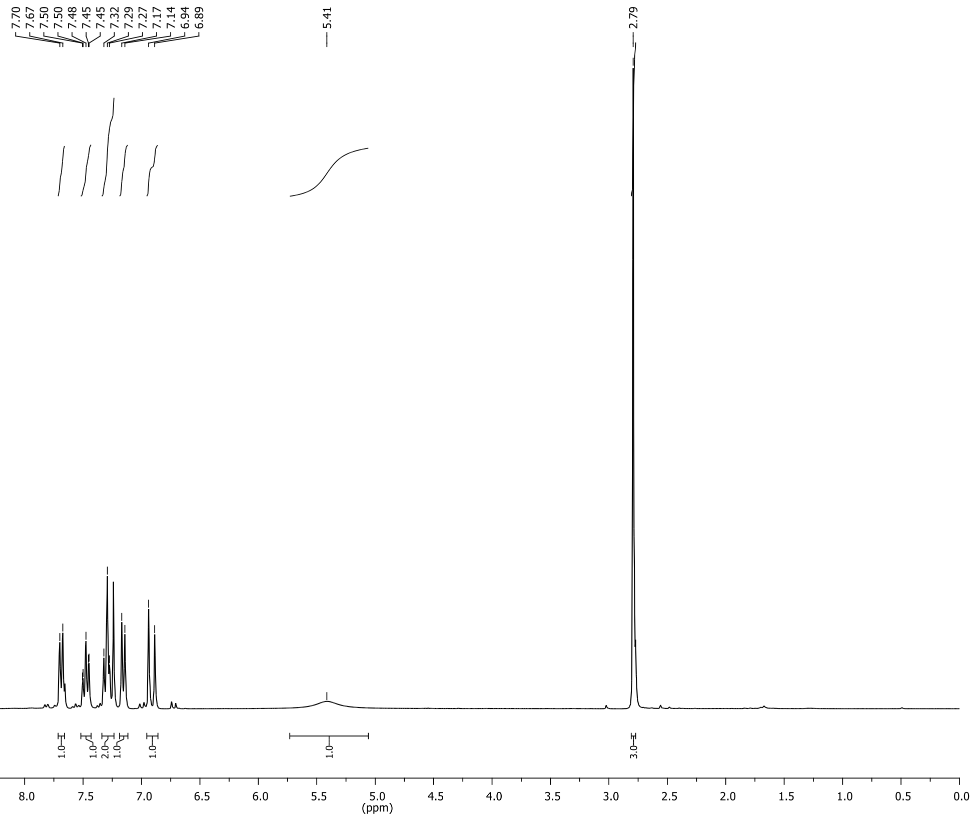

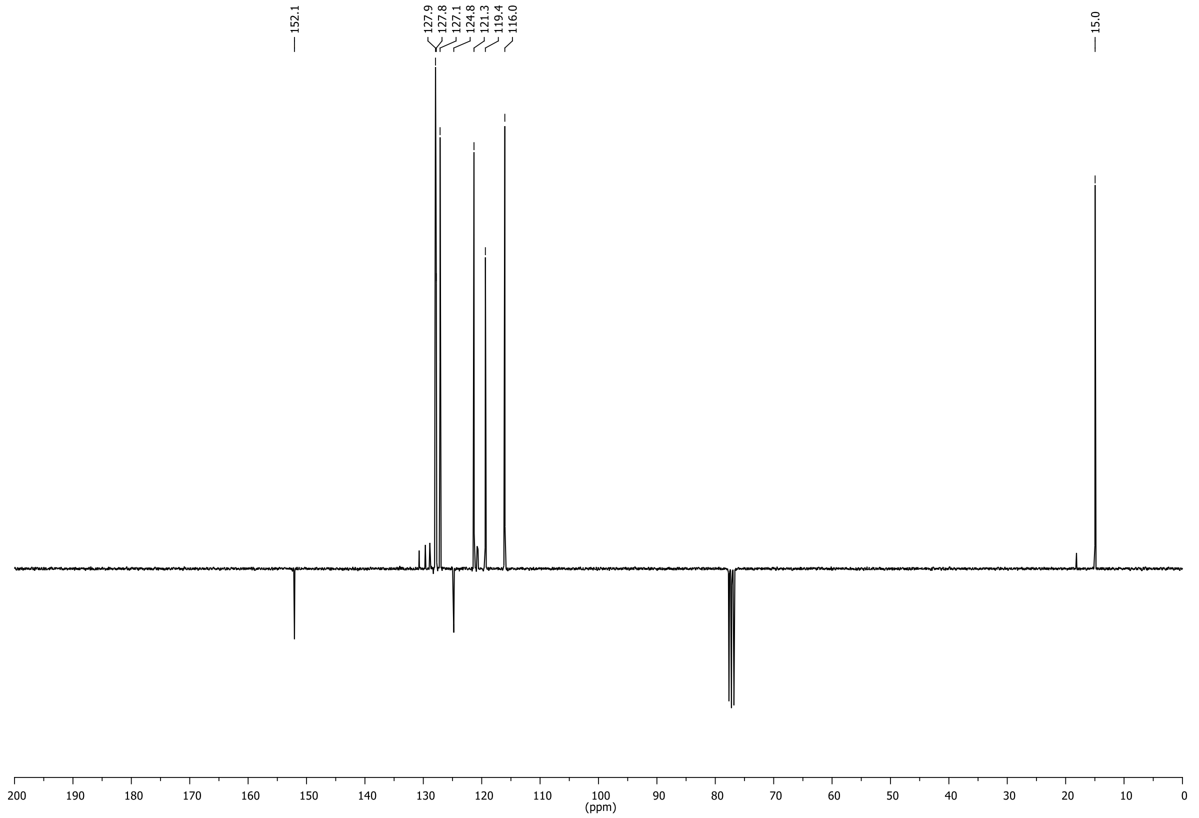

2-(2-(Methylthio)ethyl)phenol (**11**)

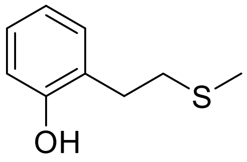

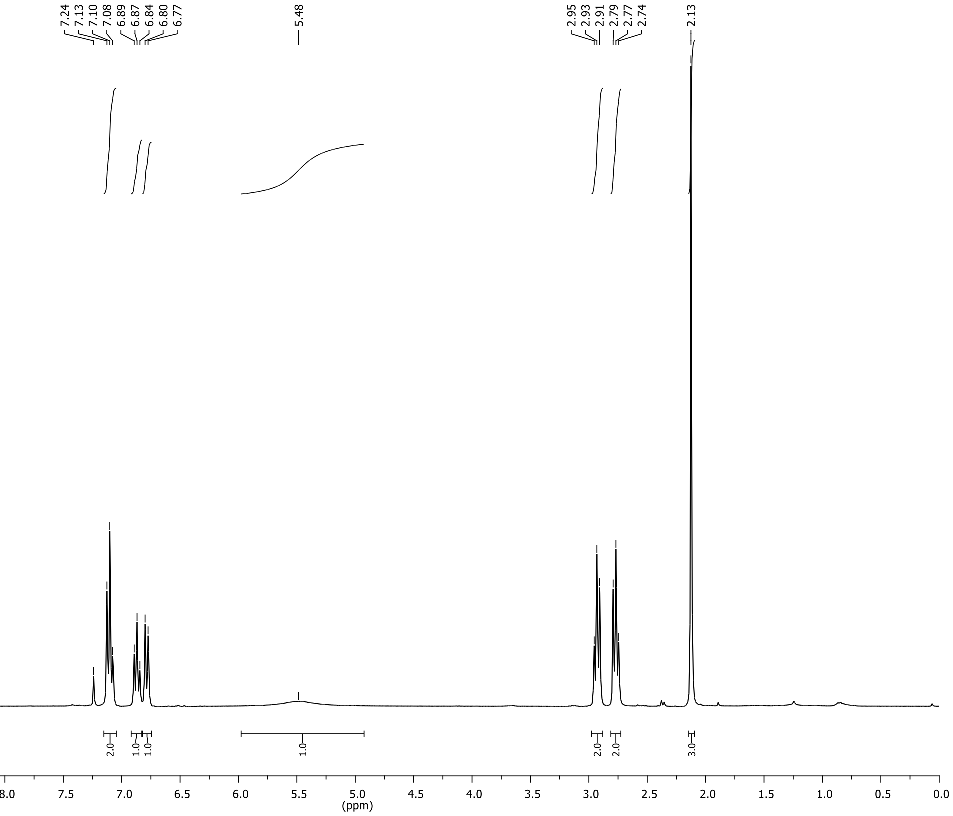

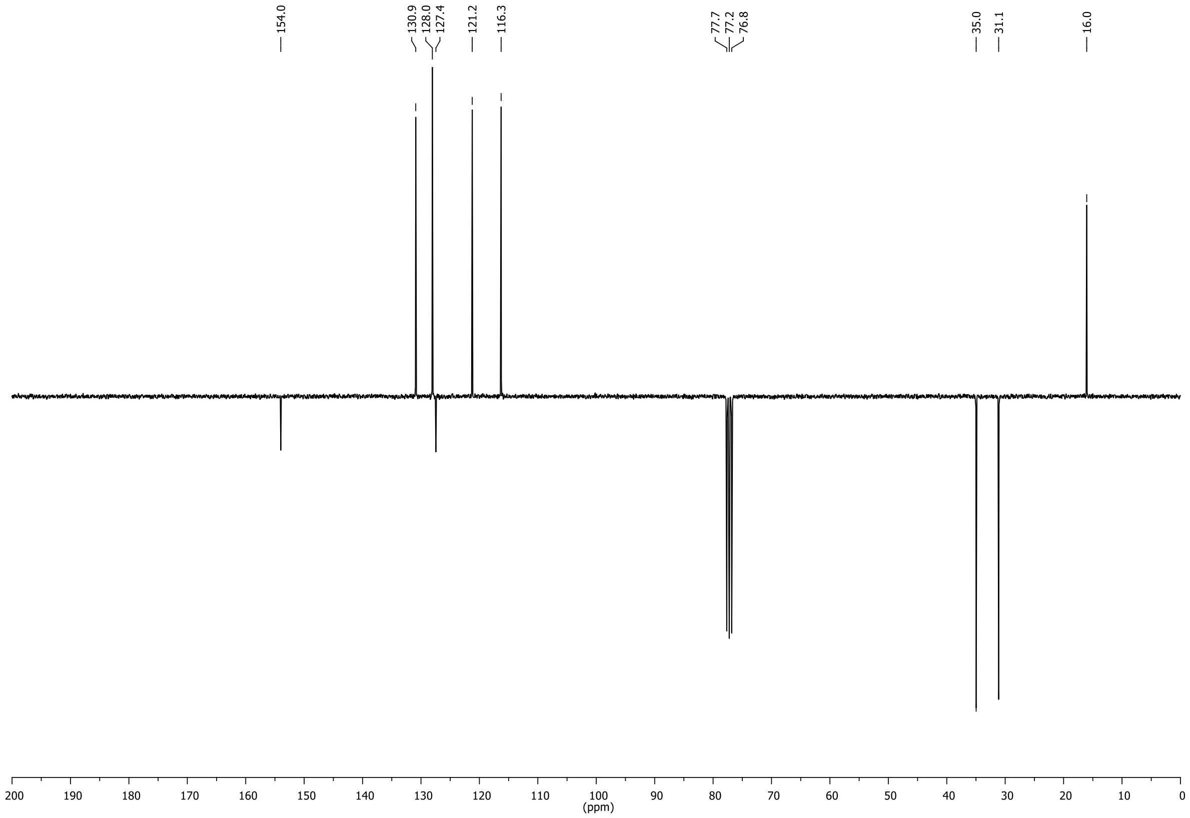

2-Hydroxy-5-iodobenzaldehyde (**13**)

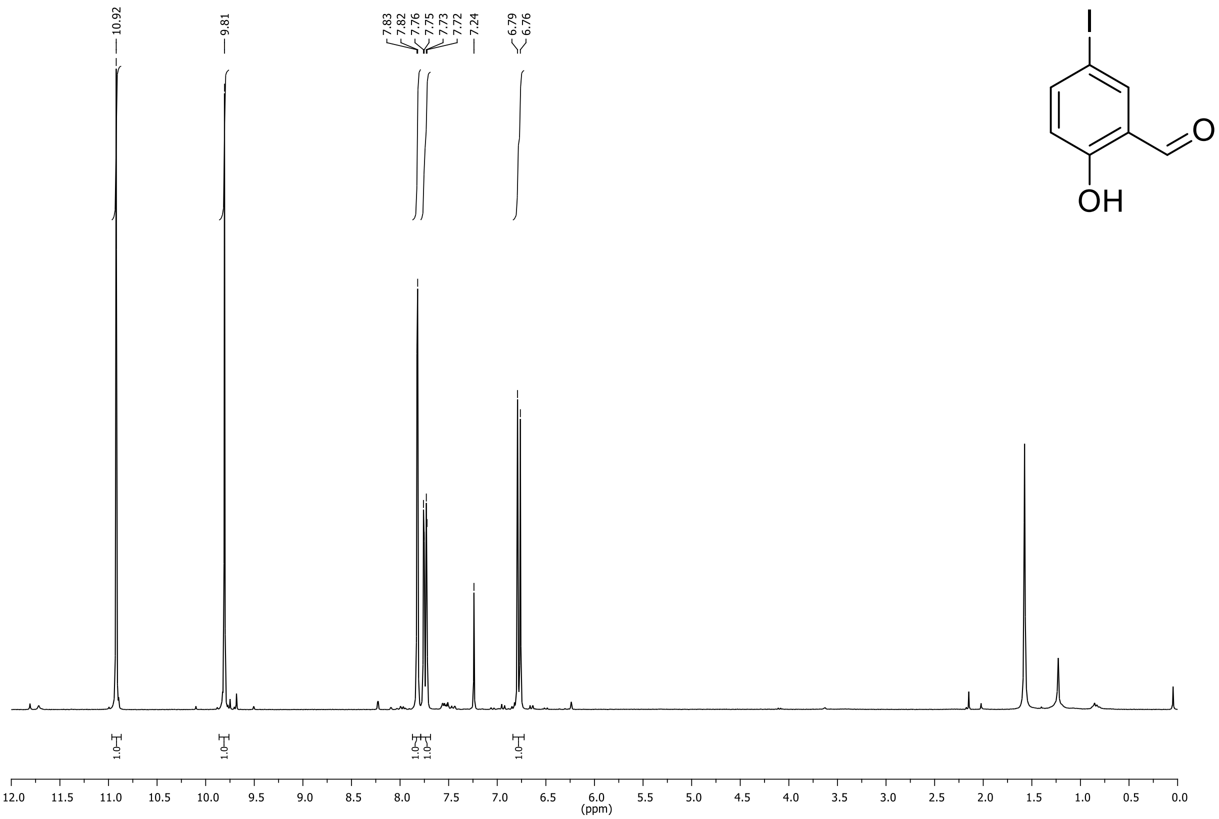

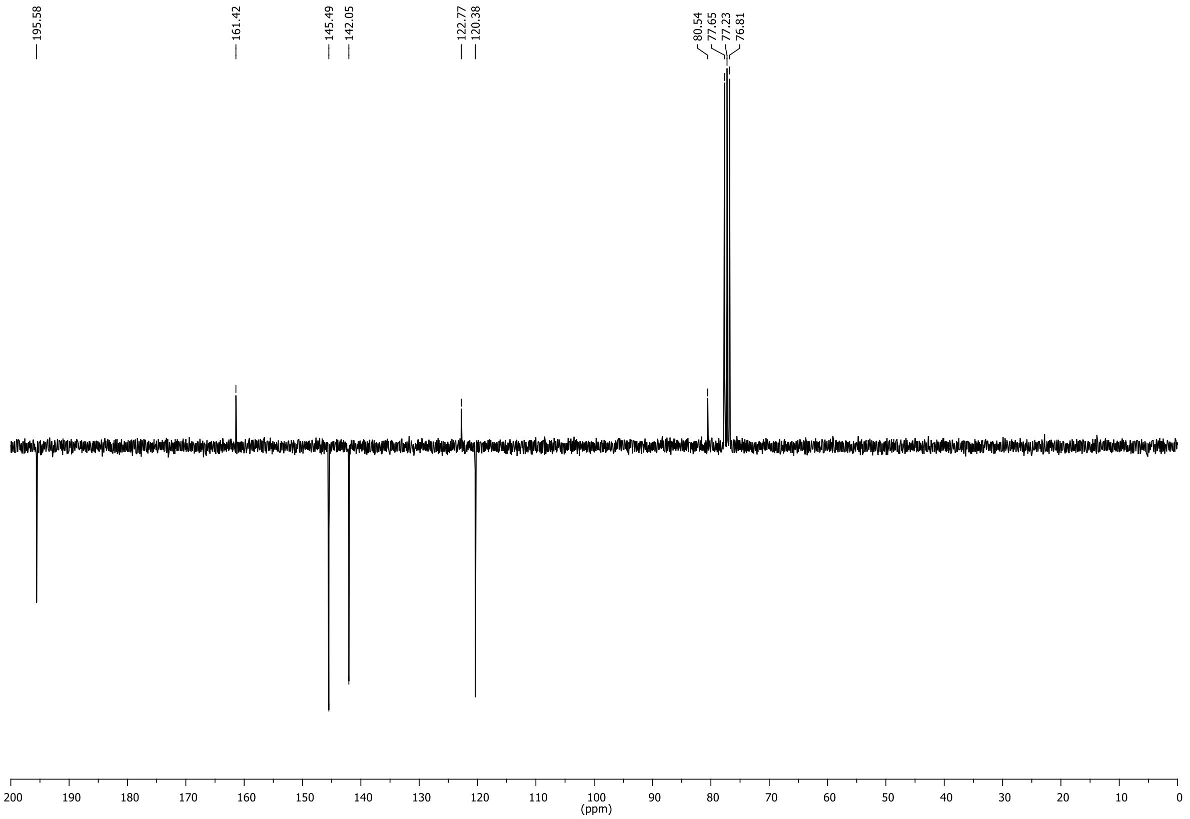

4-Iodo-2-(2-(methylthio)vinyl)phenol (**14**)

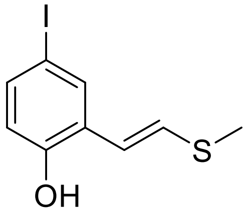

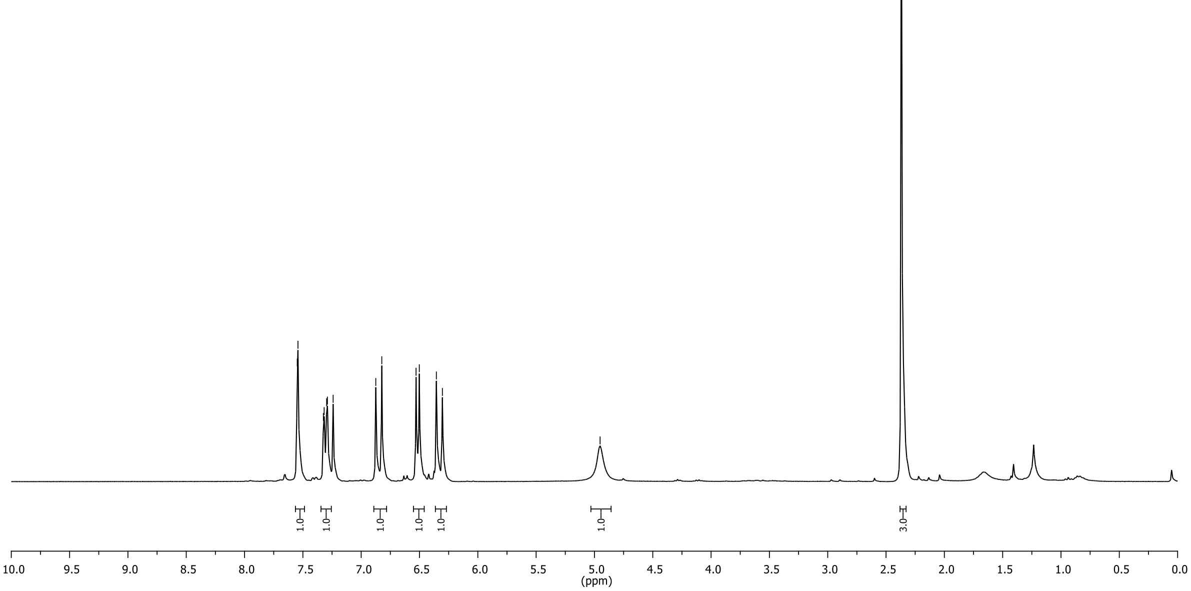

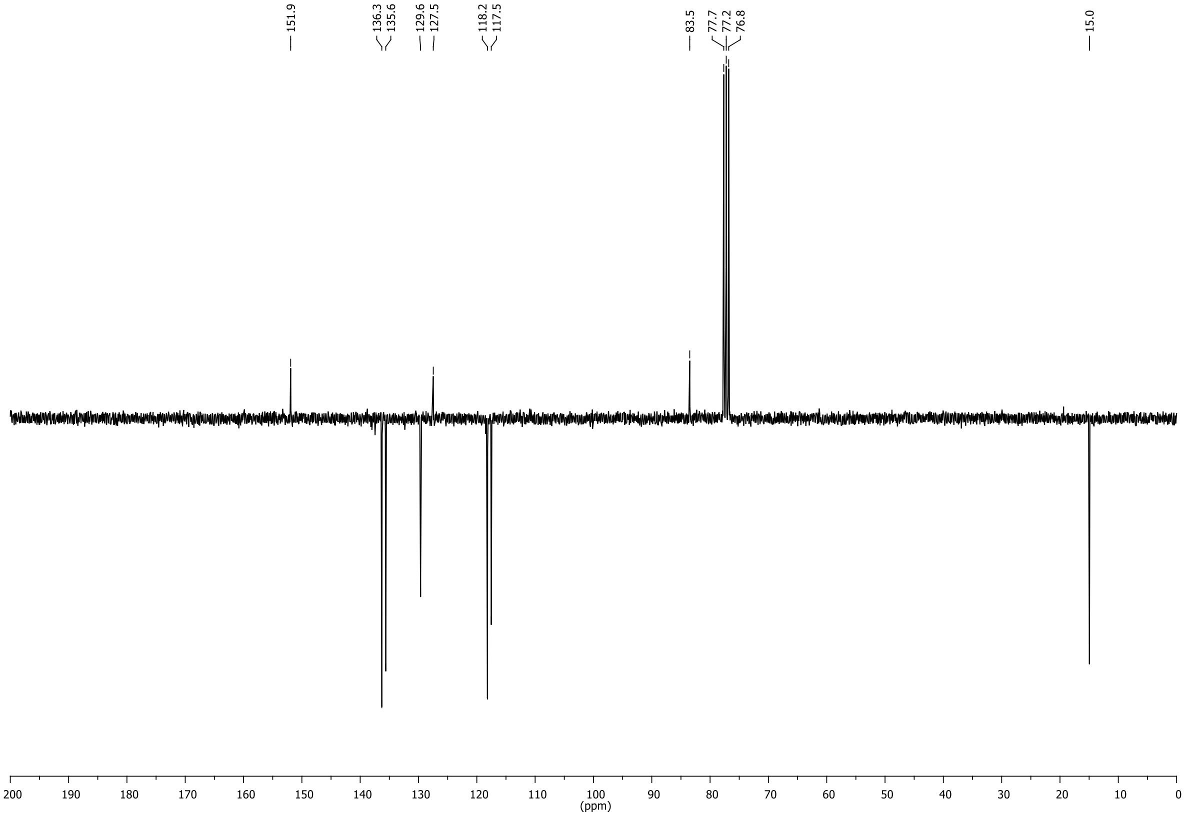

4-Iodo-2-(2-(methylthio)ethyl)phenol (**12a**)

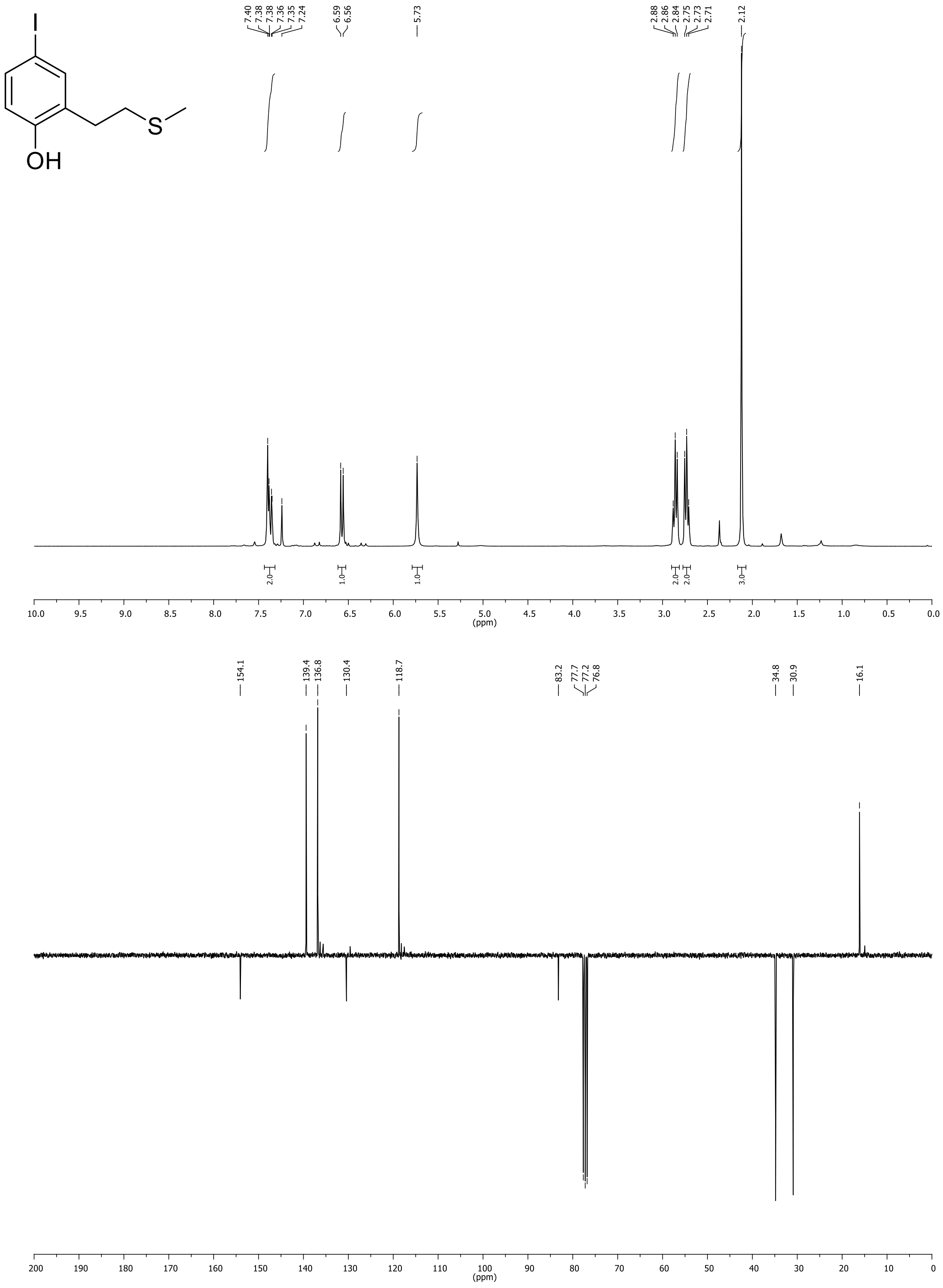

4-Iodo-2-(2-(methylthio)ethyl)phenyl trifluoromethanesulfonate (**12**)

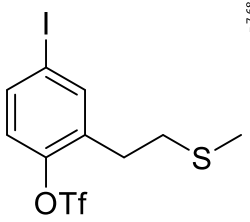

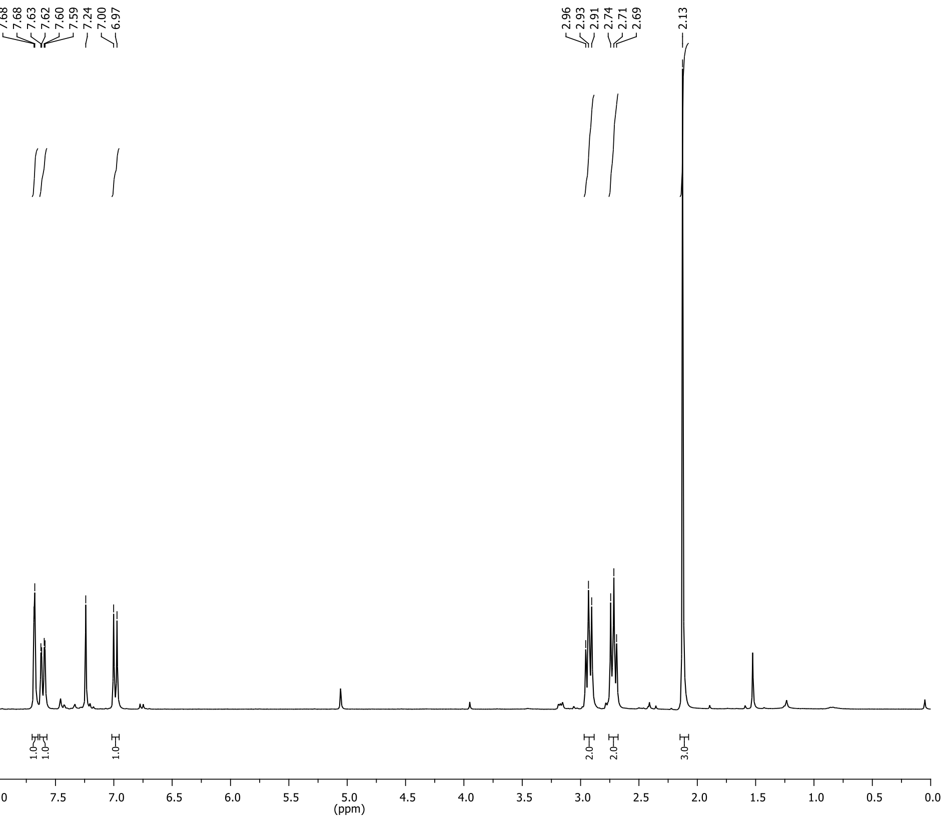

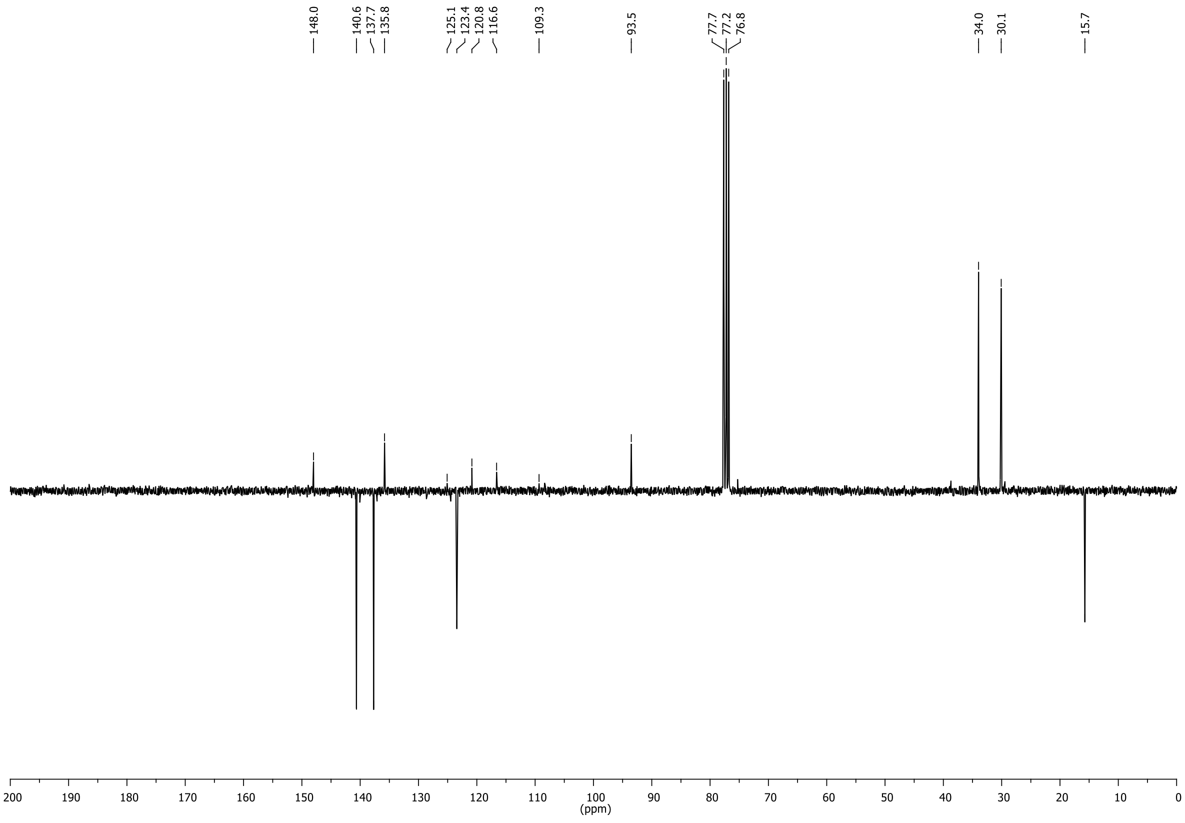

Tert-butyl(4-iodophenoxy)diphenylsilane (**18**)

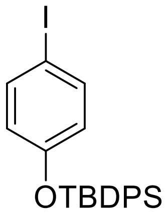

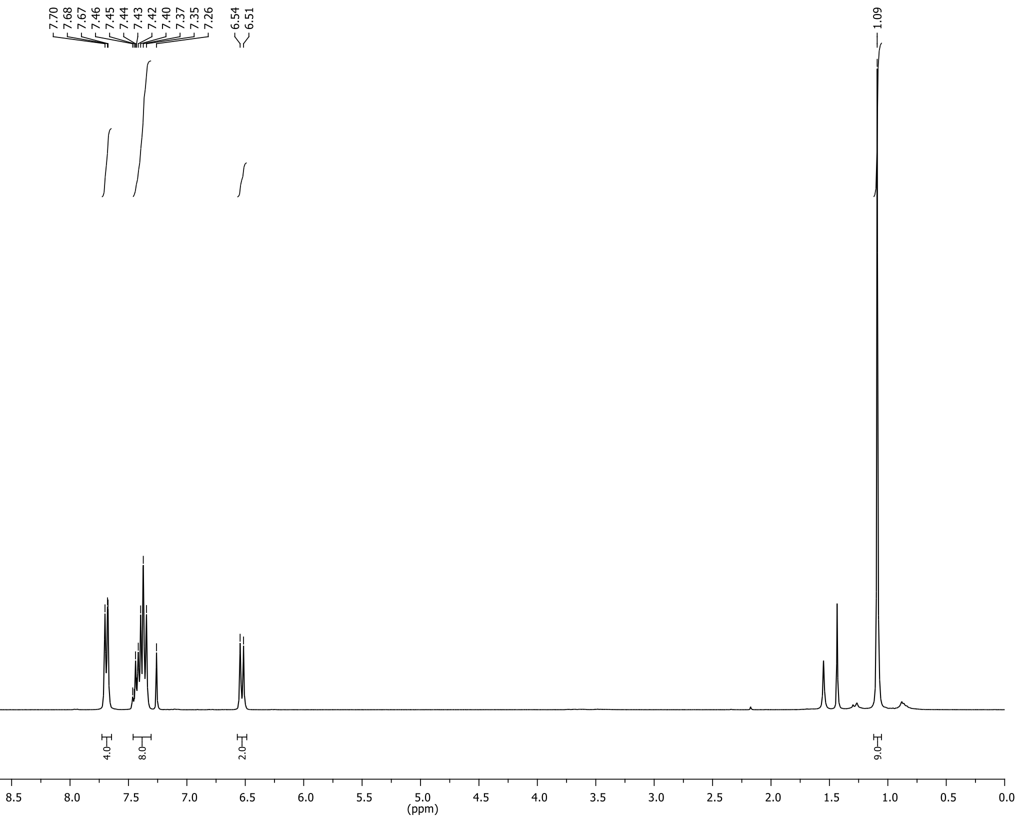

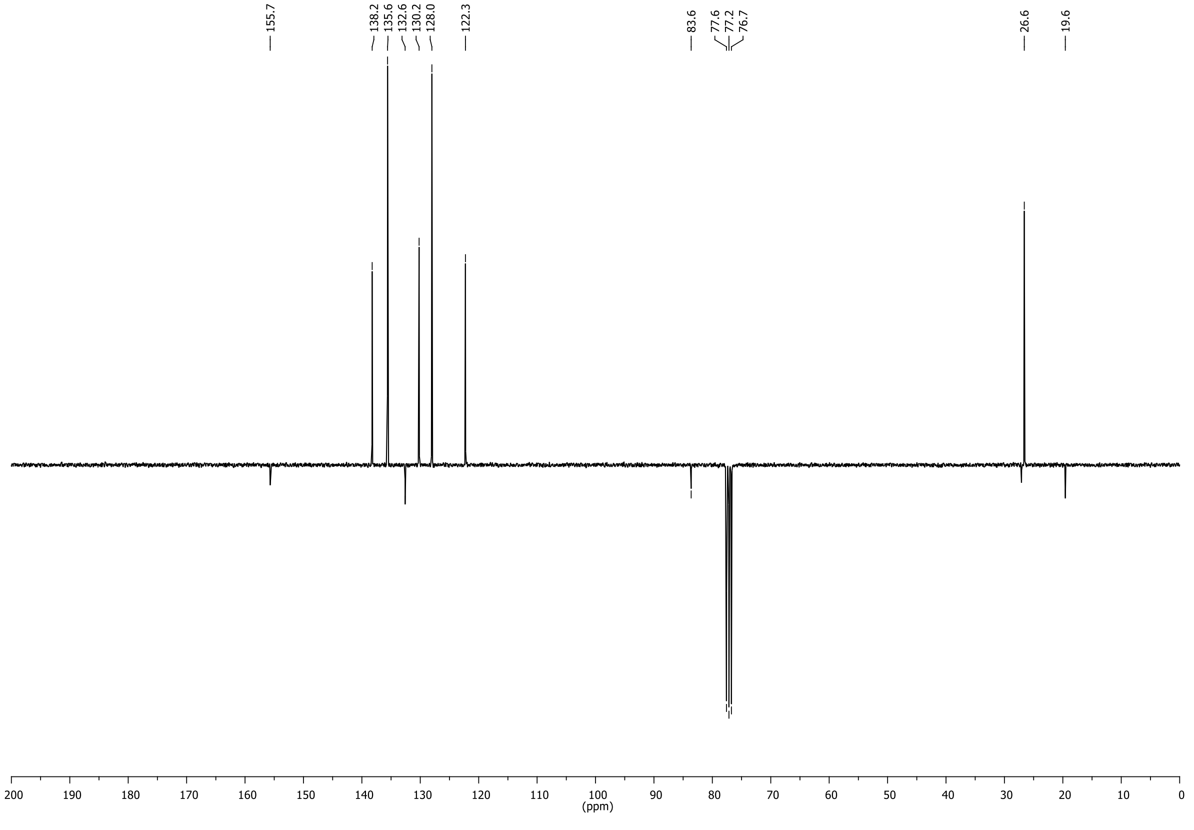

5-Iodo-2-((2-methoxyethoxy)methoxy)benzaldehyde (**17**)

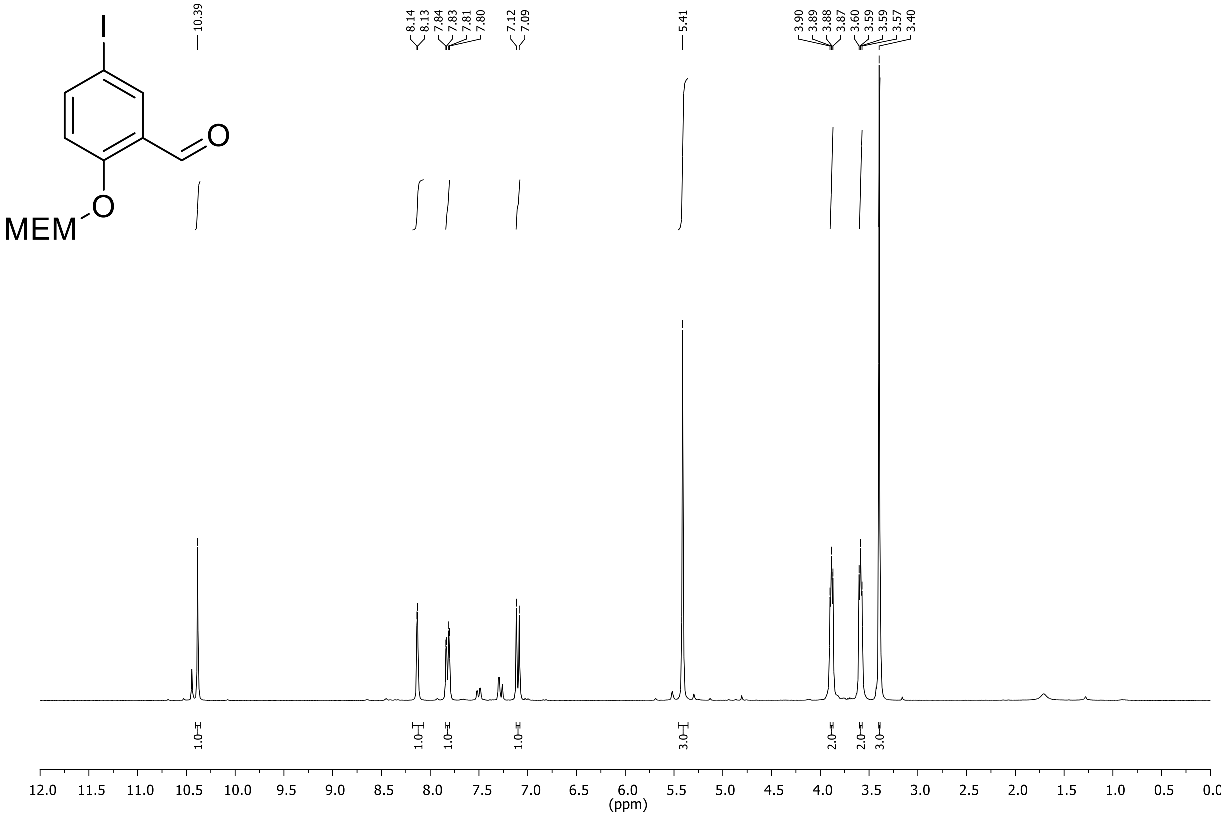

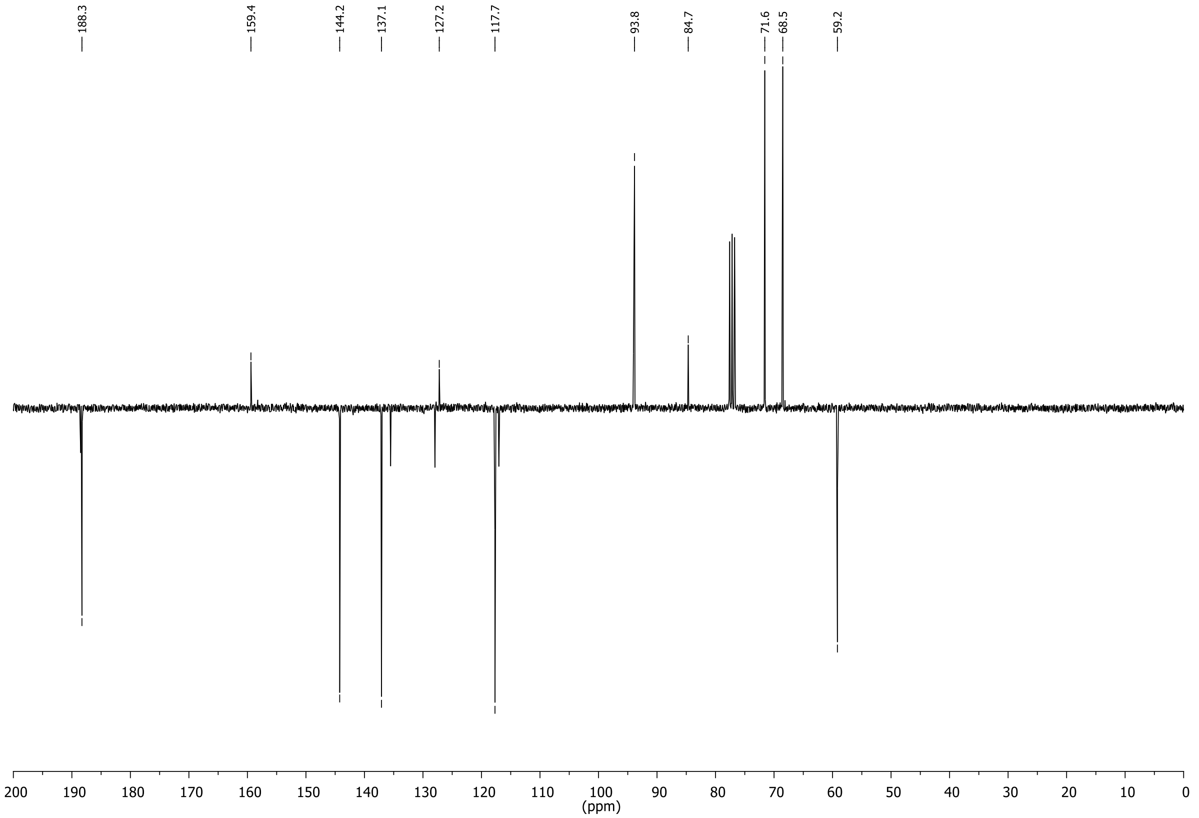

(4-((*Tert*-butyldiphenylsilyl)oxy)phenyl)(5-iodo-2-((2-methoxyethoxy)methoxy)phenyl)methanol (**19**)

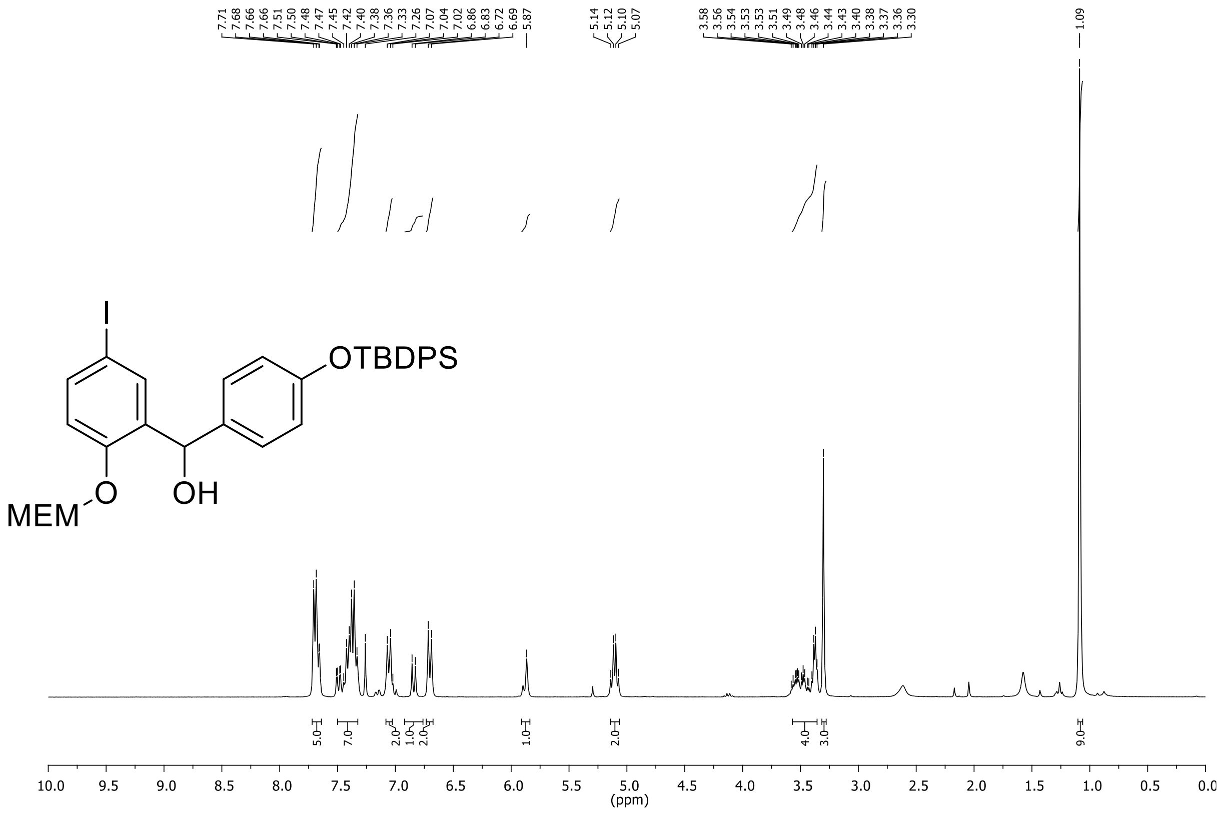

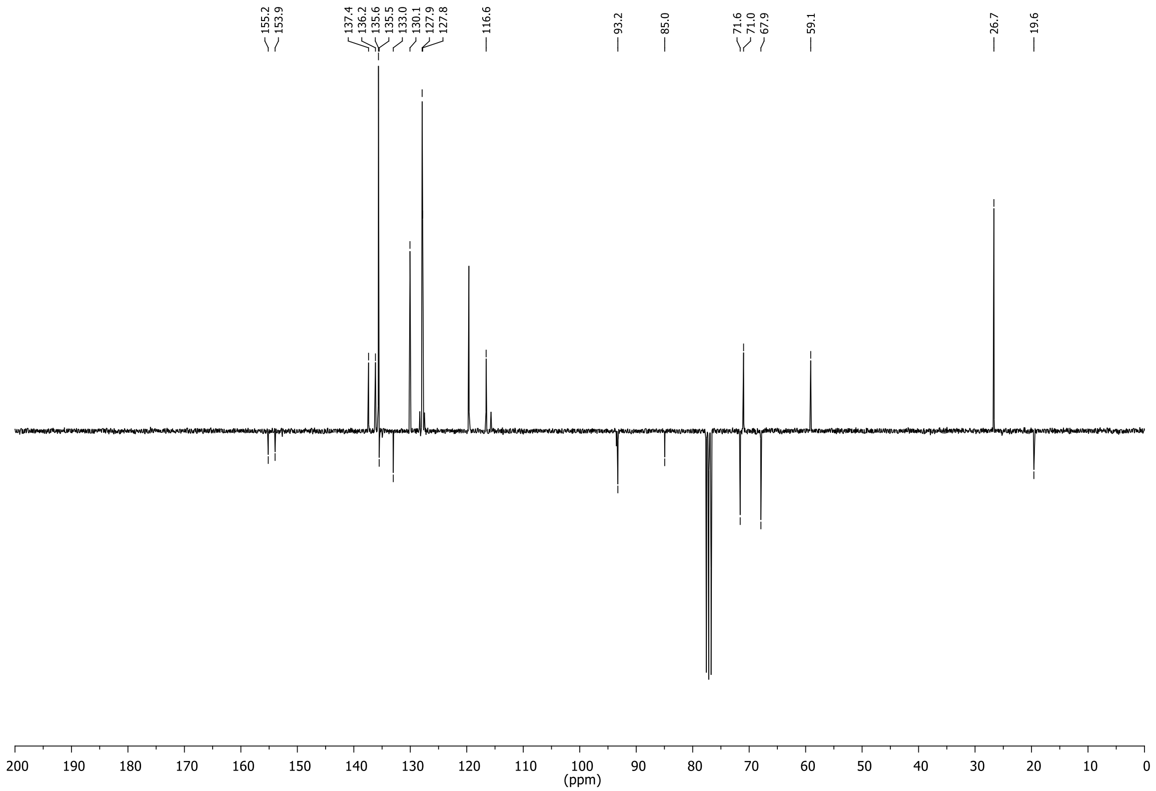

2-(4-((*tert*-Butyldiphenylsilyl)oxy)benzyl)-4-iodophenol (**20a**)

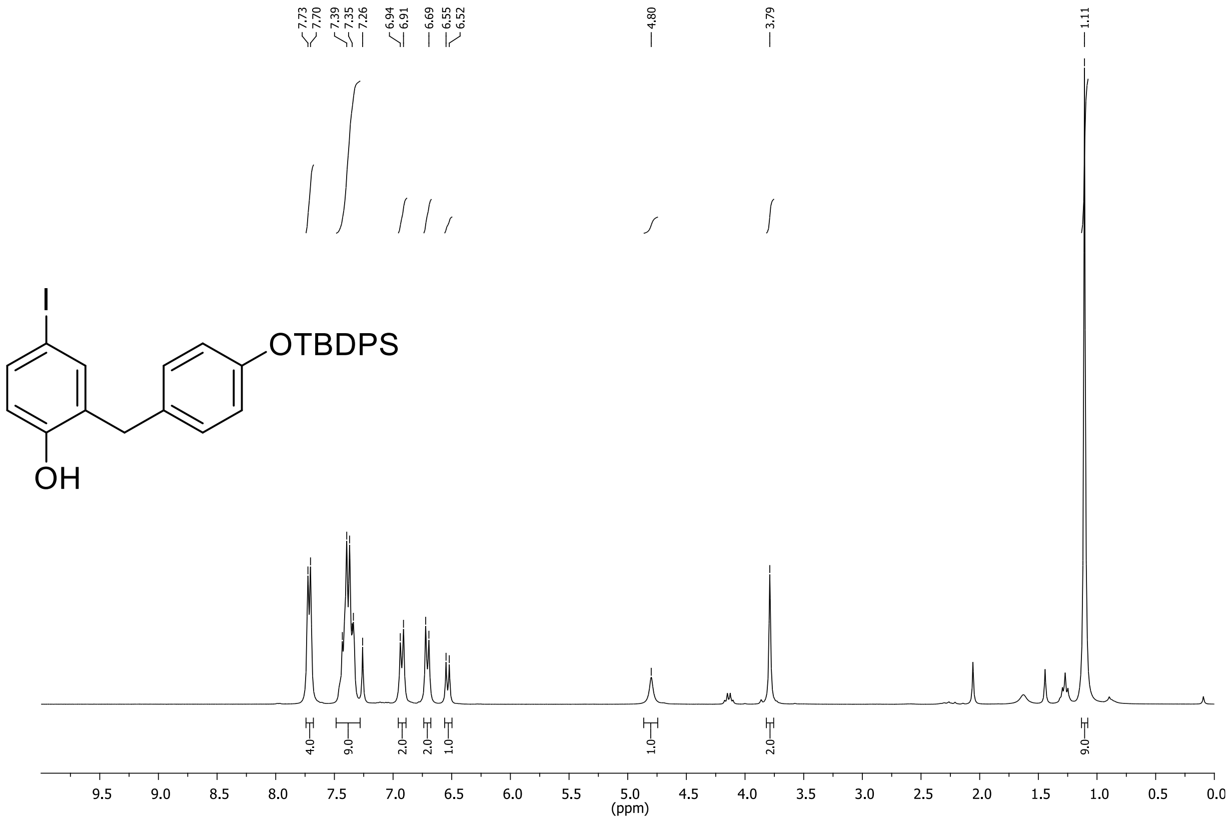

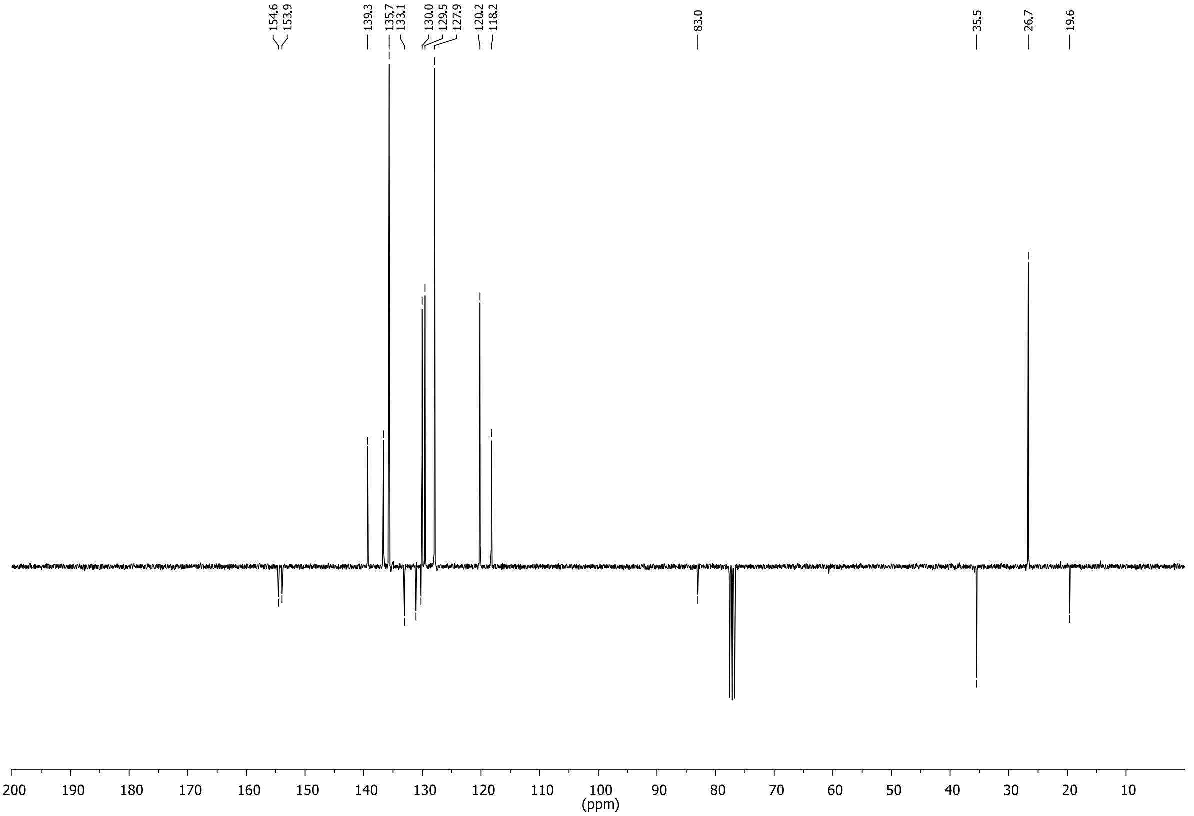

2-(4-((*tert*-Butyldiphenylsilyl)oxy)benzyl)-4-iodophenyl trifluoromethanesulfonate (**20**)

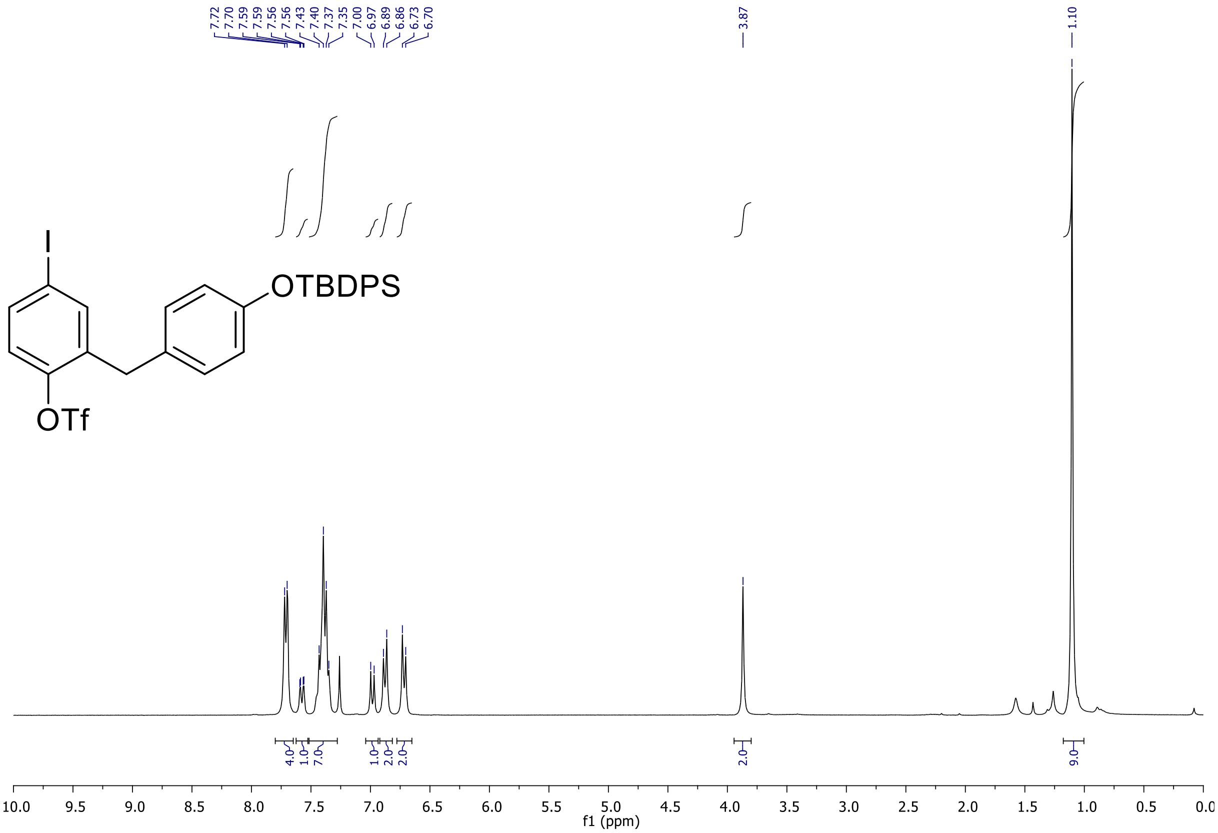

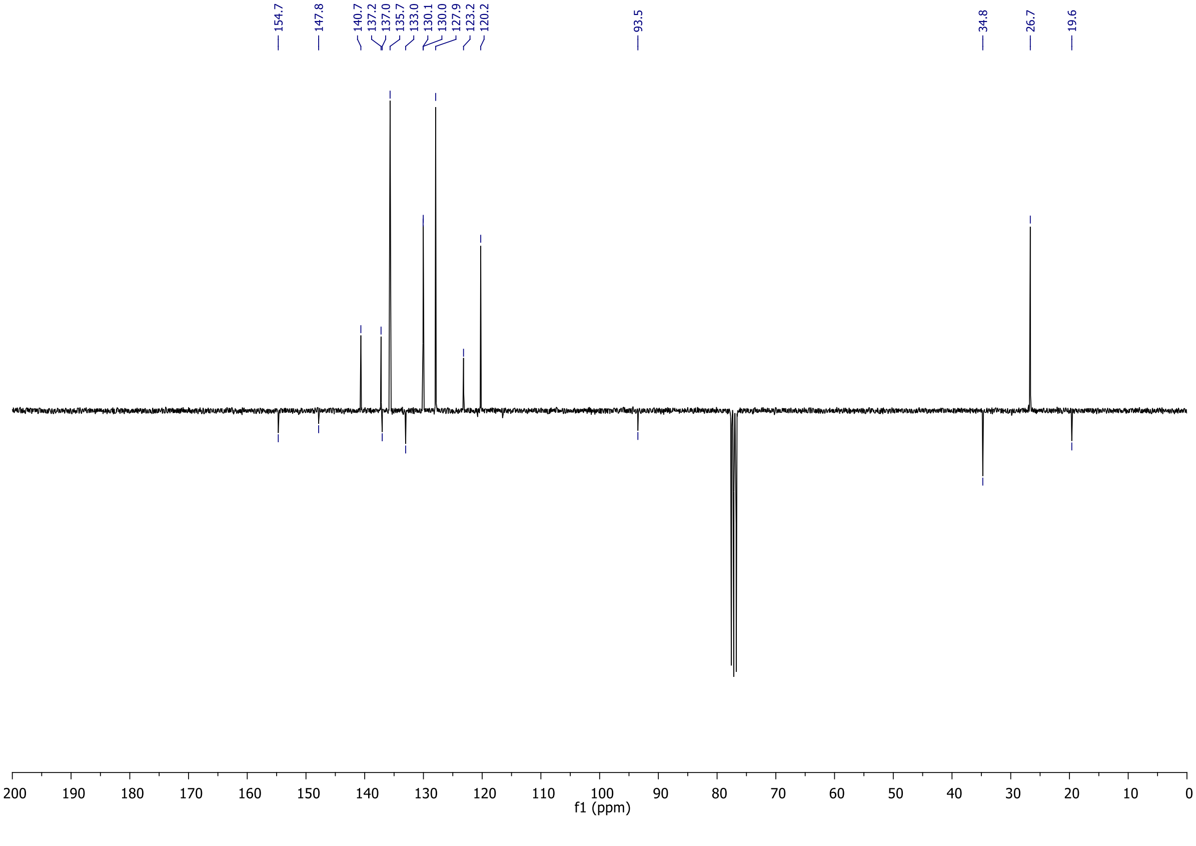

2-(hydroxymethyl)-4-iodophenol (**21**)

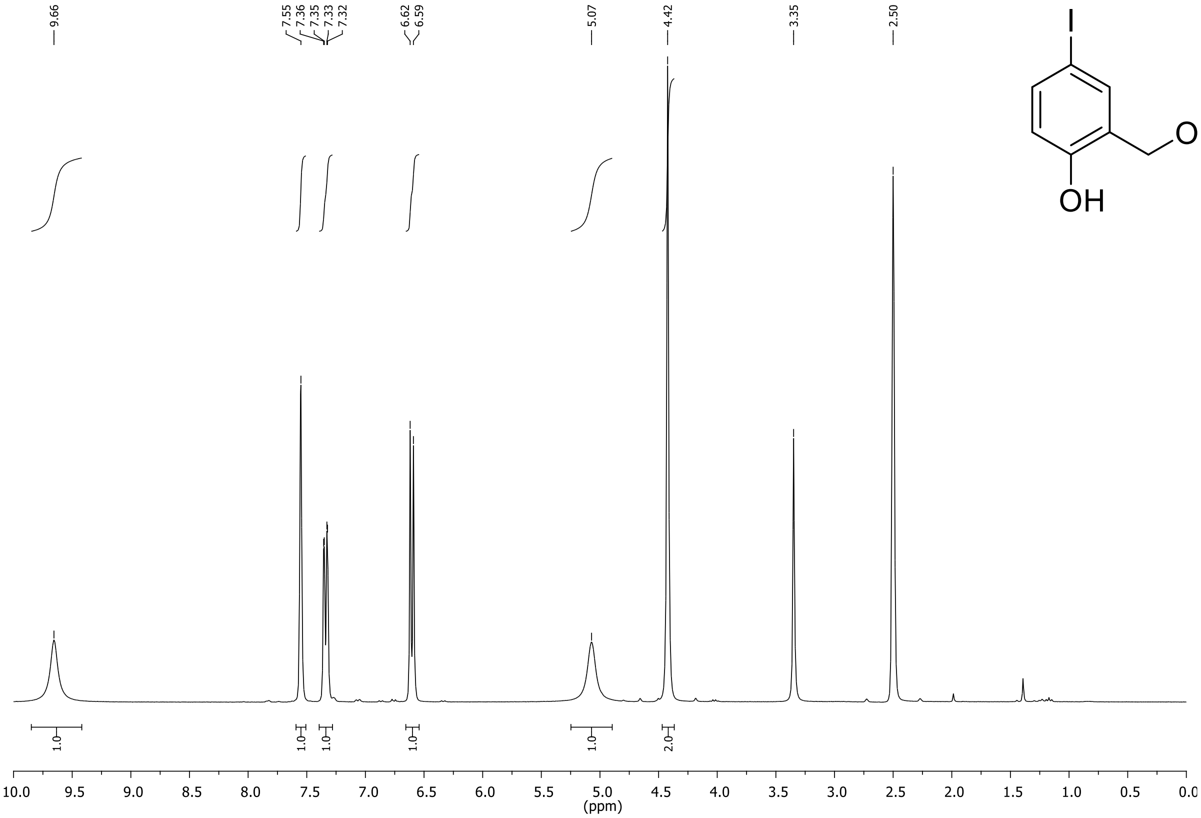

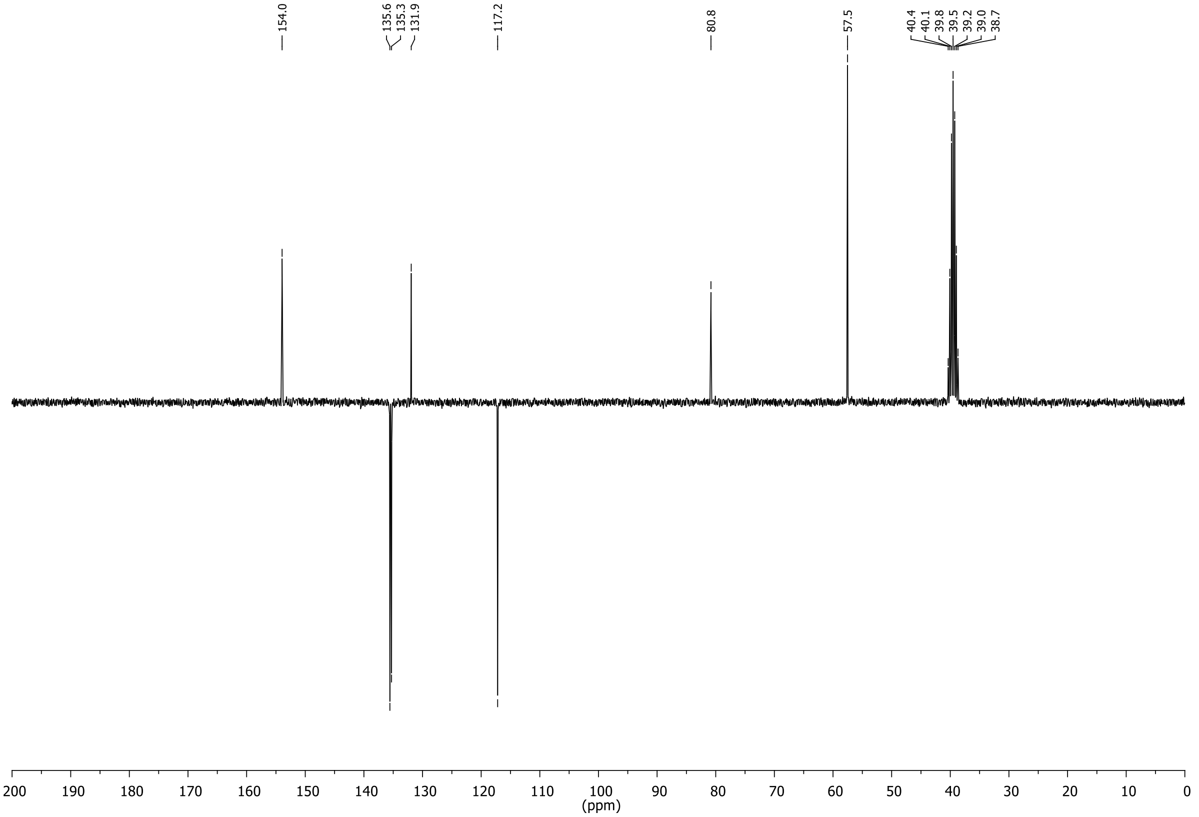

2-((1H-Indol-3-yl)methyl)-4-iodophenol (**22a**)

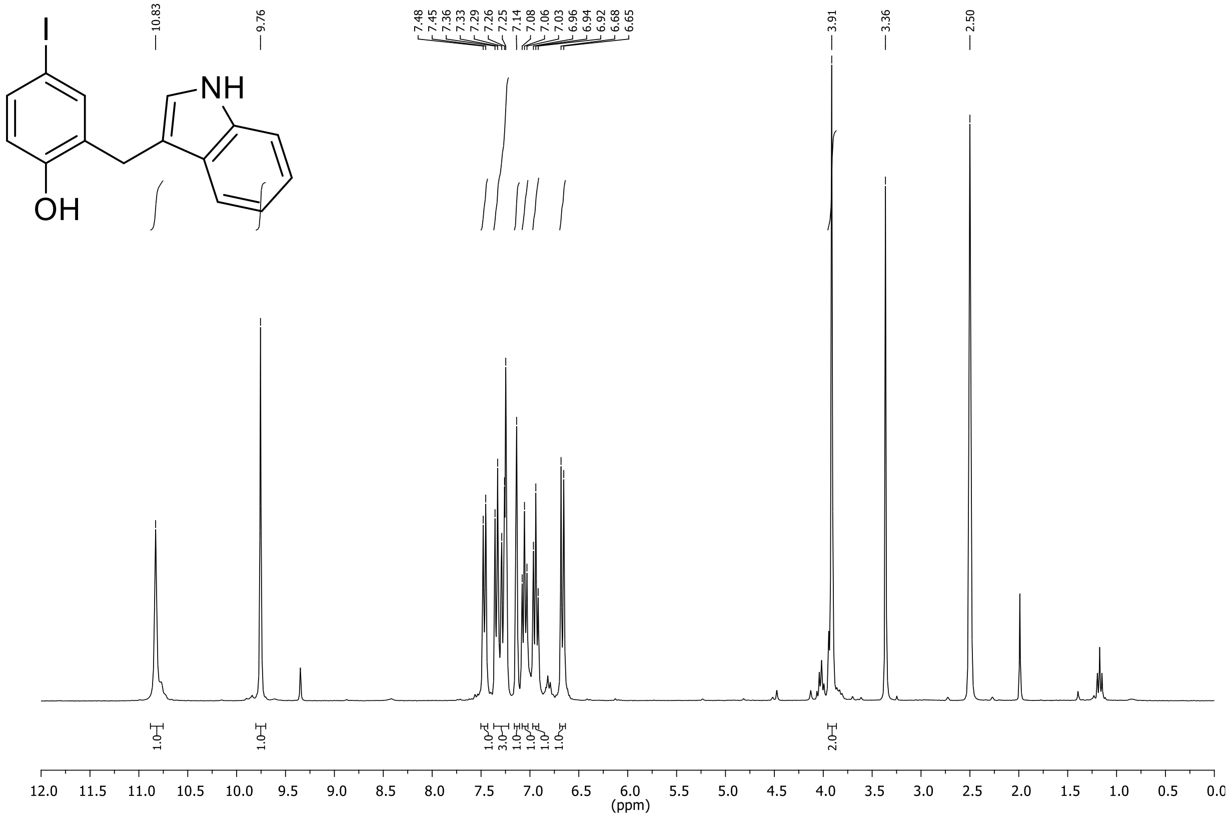

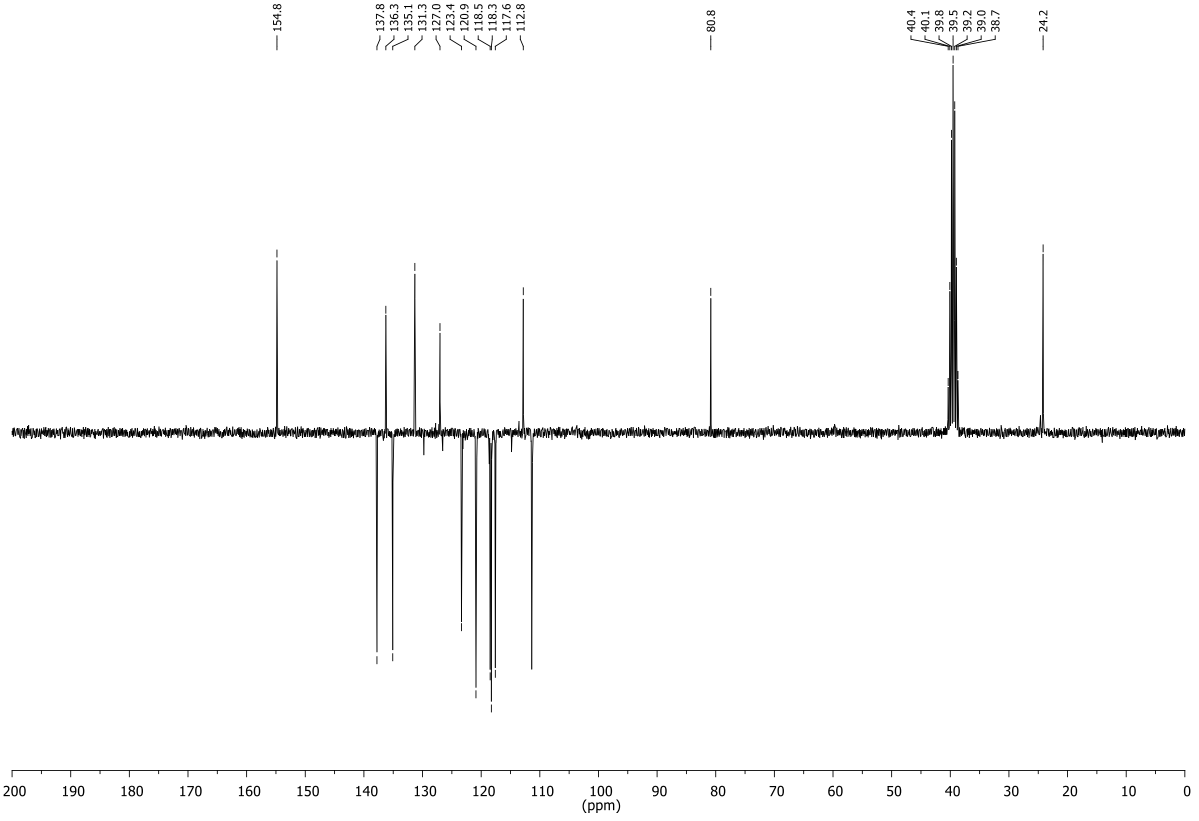

2-((1H-Indol-3-yl)methyl)-4-iodophenyl trifluoromethanesulfonate (**22**)

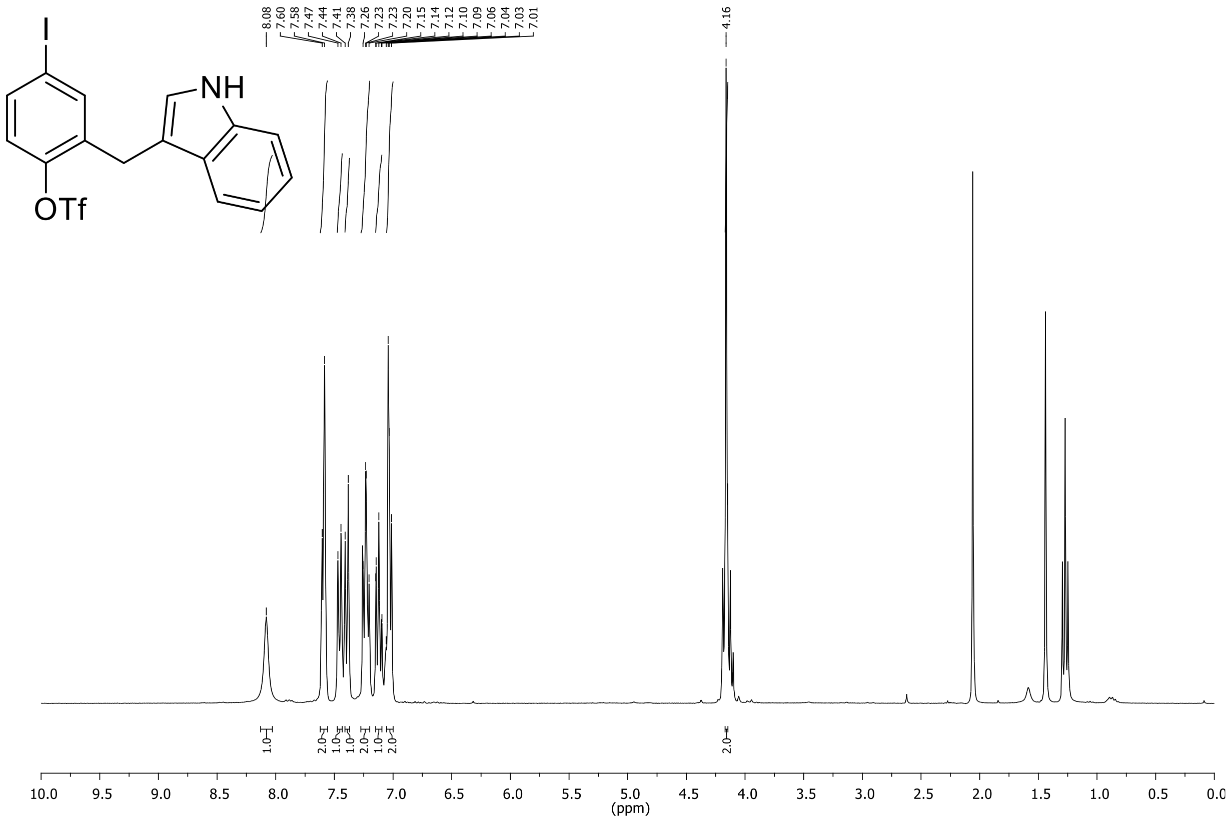

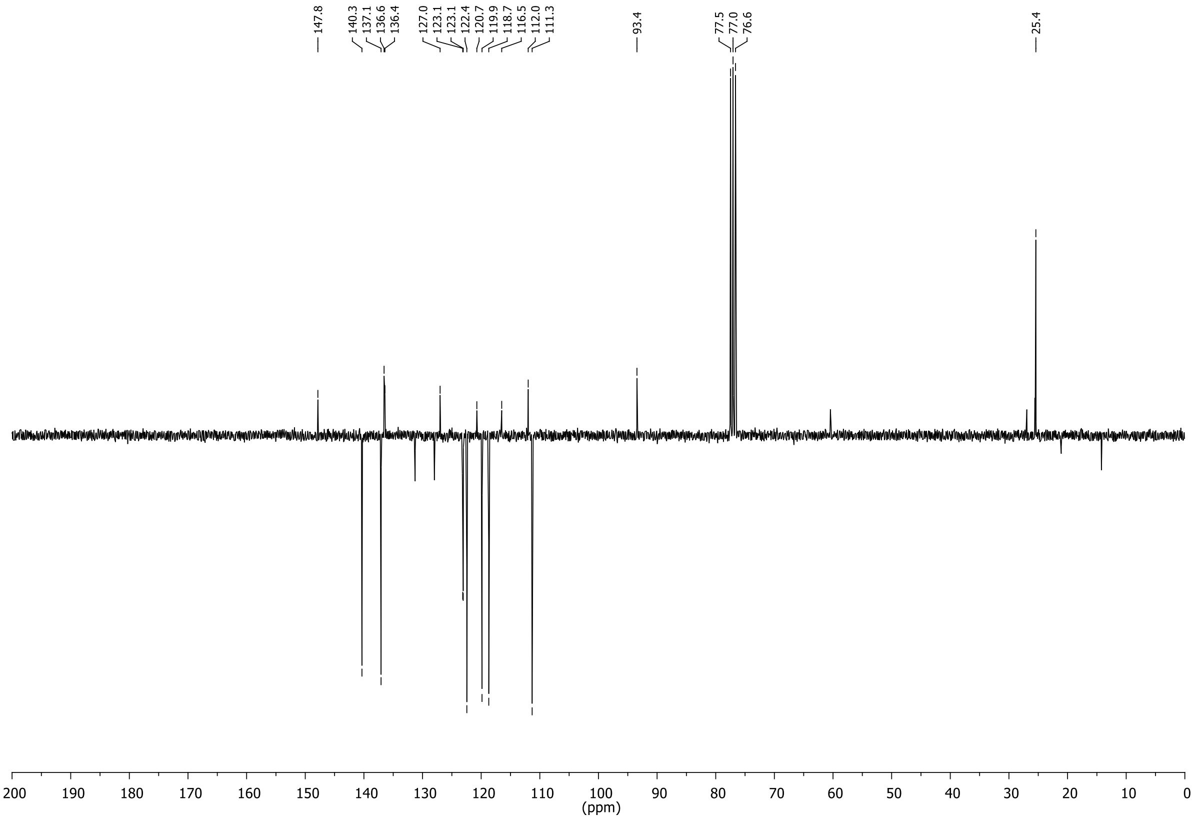

6-Iodochroman-2-one (**23a**)

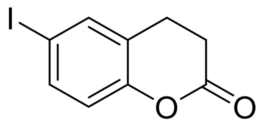

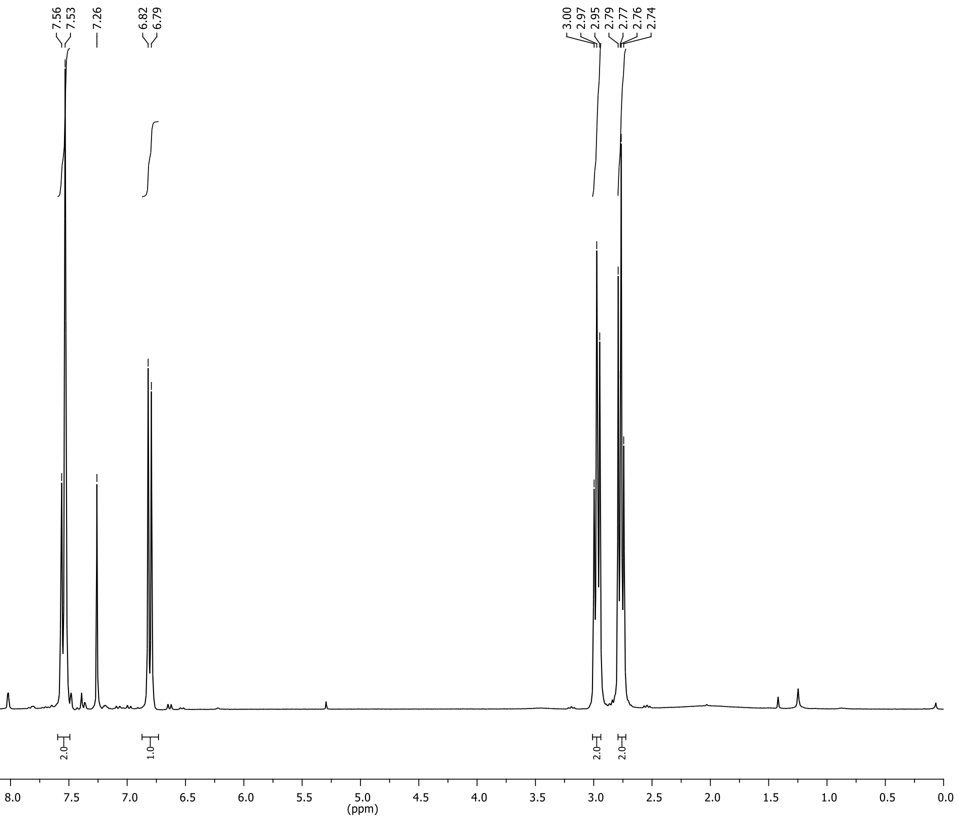

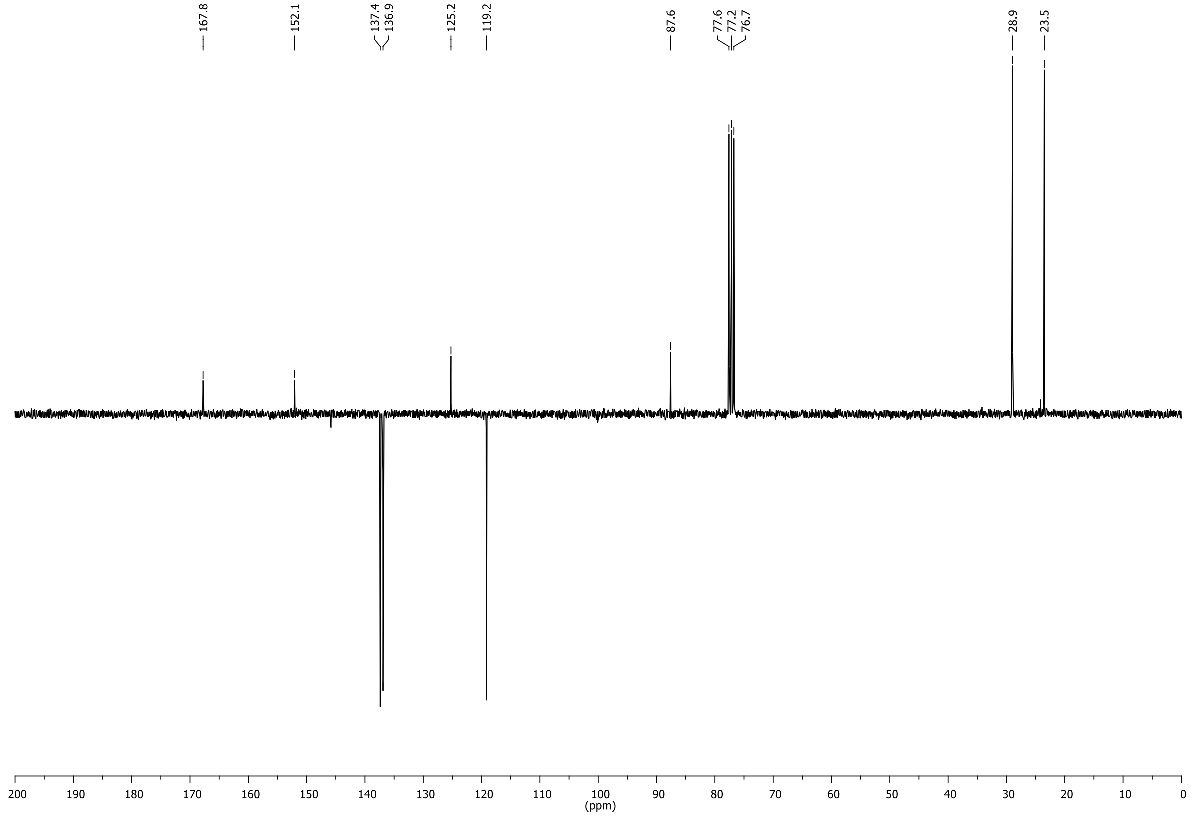

Methyl 3-(2-hydroxy-5-iodophenyl)propanoate (**23b**)

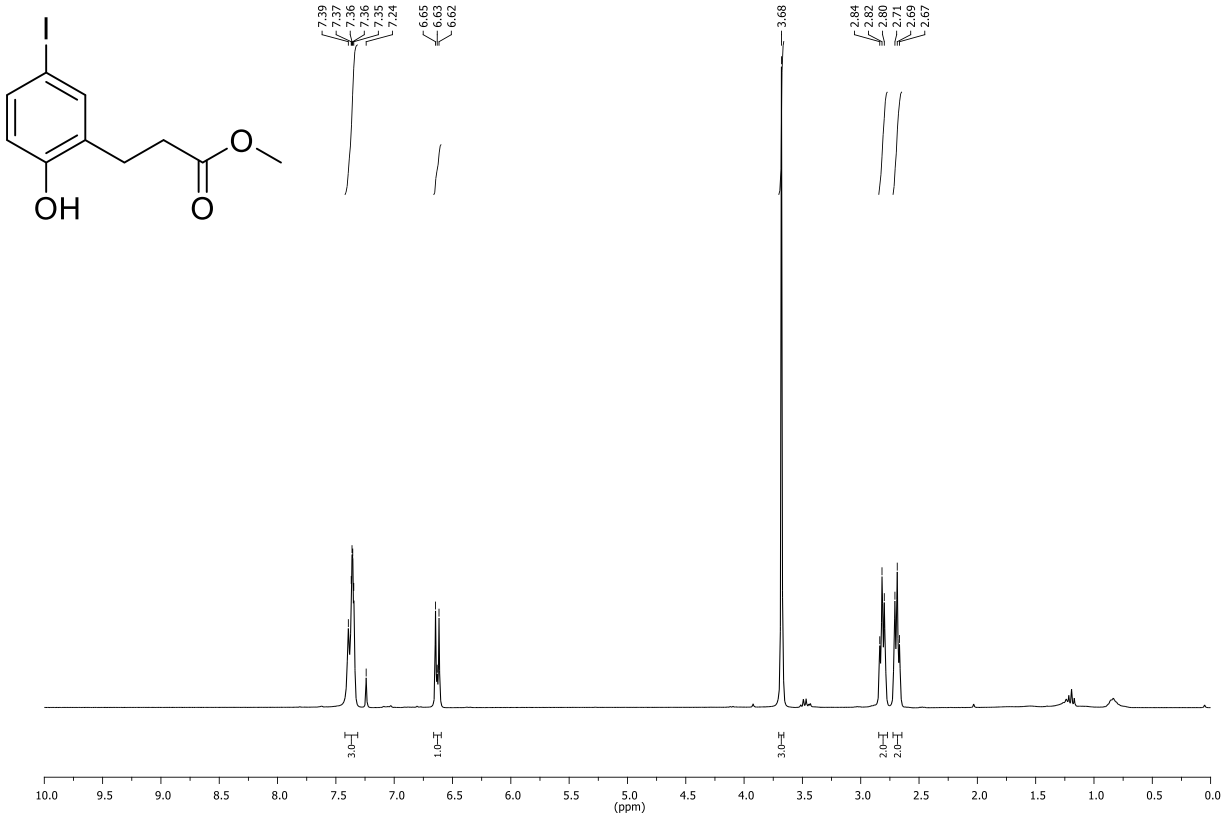

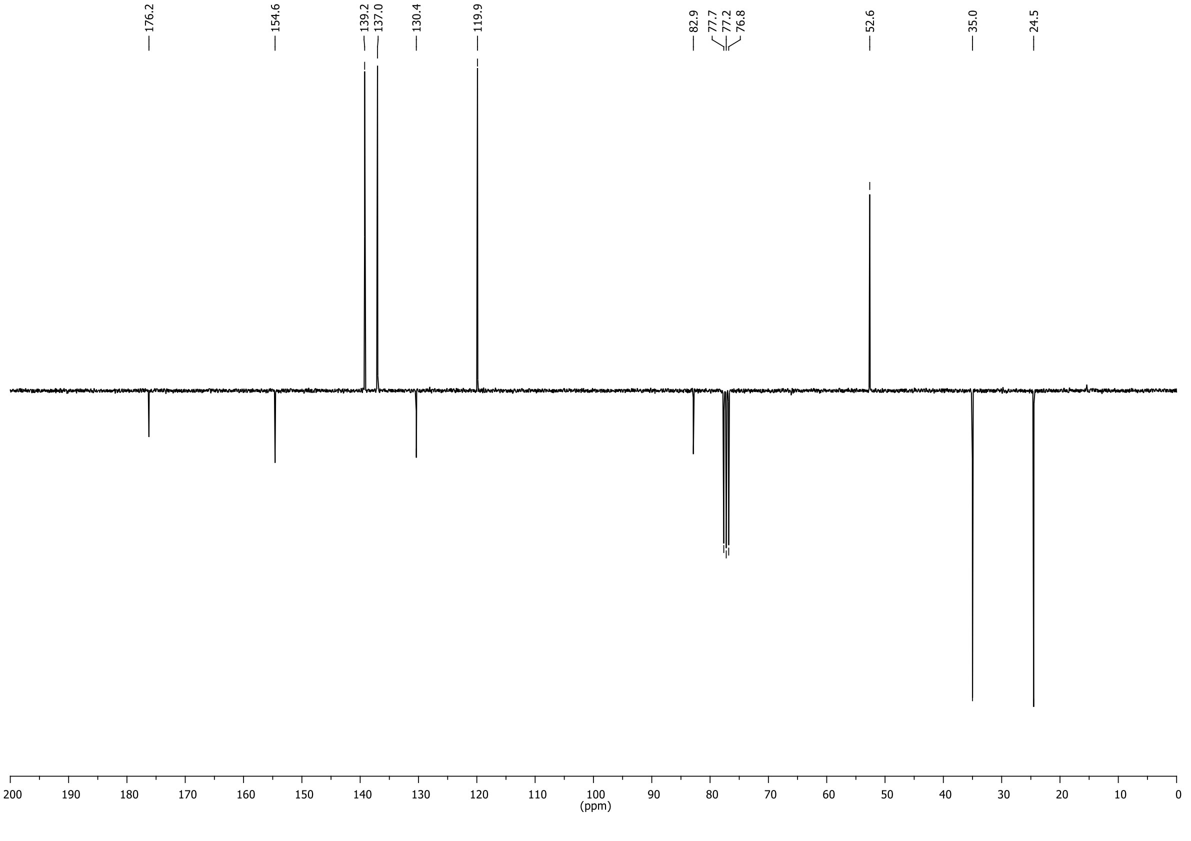

Methyl 3-(5-iodo-2-(((trifluoromethyl)sulfonyl)oxy)phenyl)propanoate (**23**)

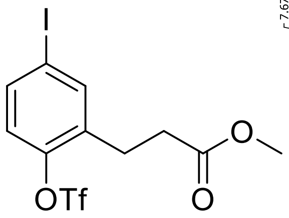

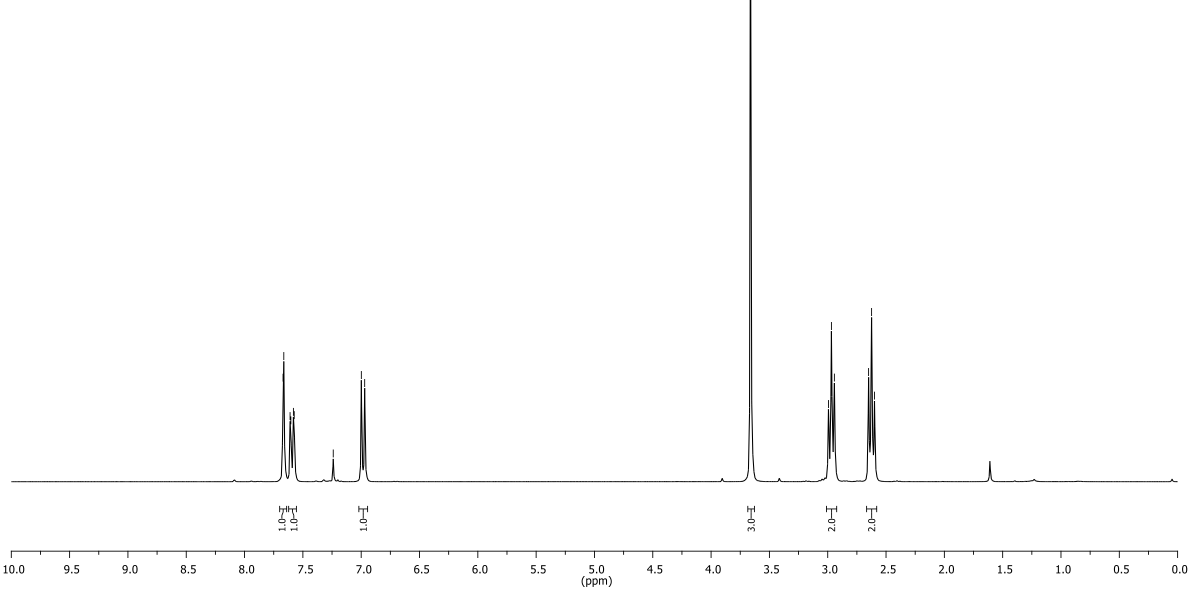

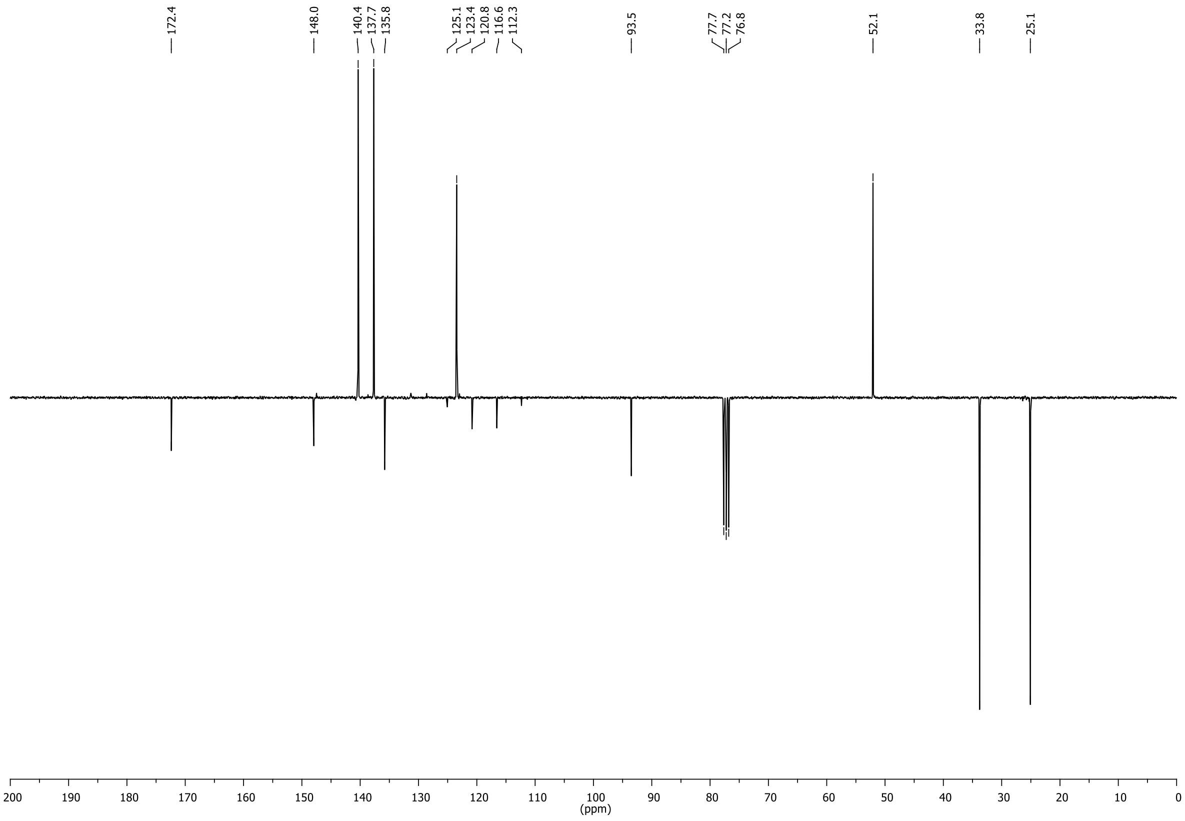

2-(3-Hydroxypropyl)-4-iodophenyl trifluoromethanesulfonate (**24**)

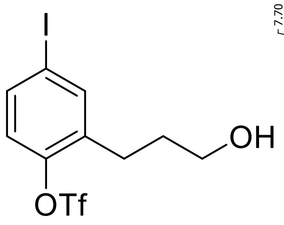

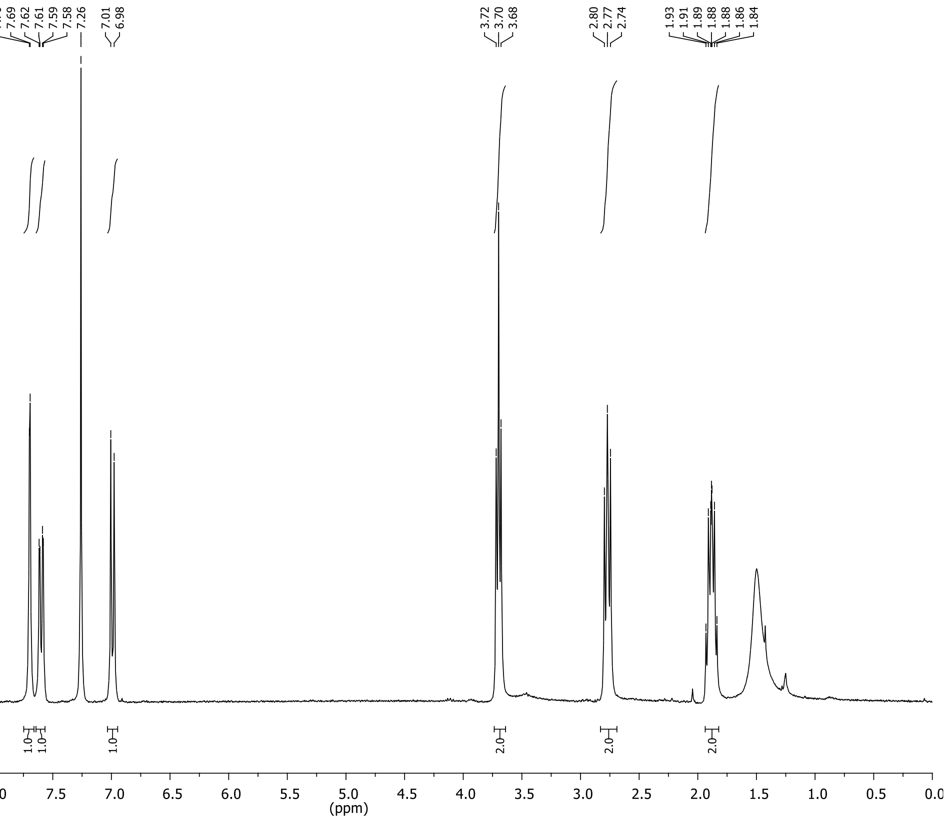

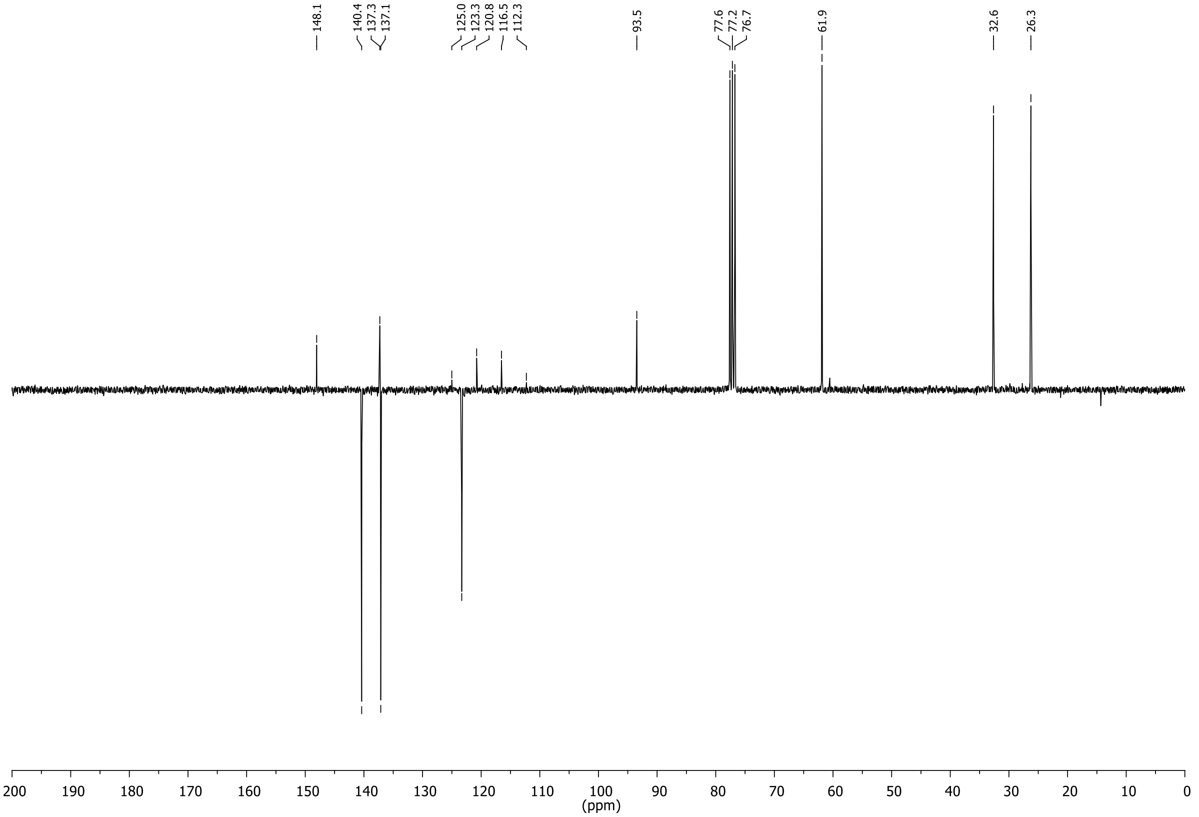

2-(3-(2,3-bis(*tert*-Butoxycarbonyl)guanidino)propyl)-4-iodophenyl trifluoromethanesulfonate (**26**)

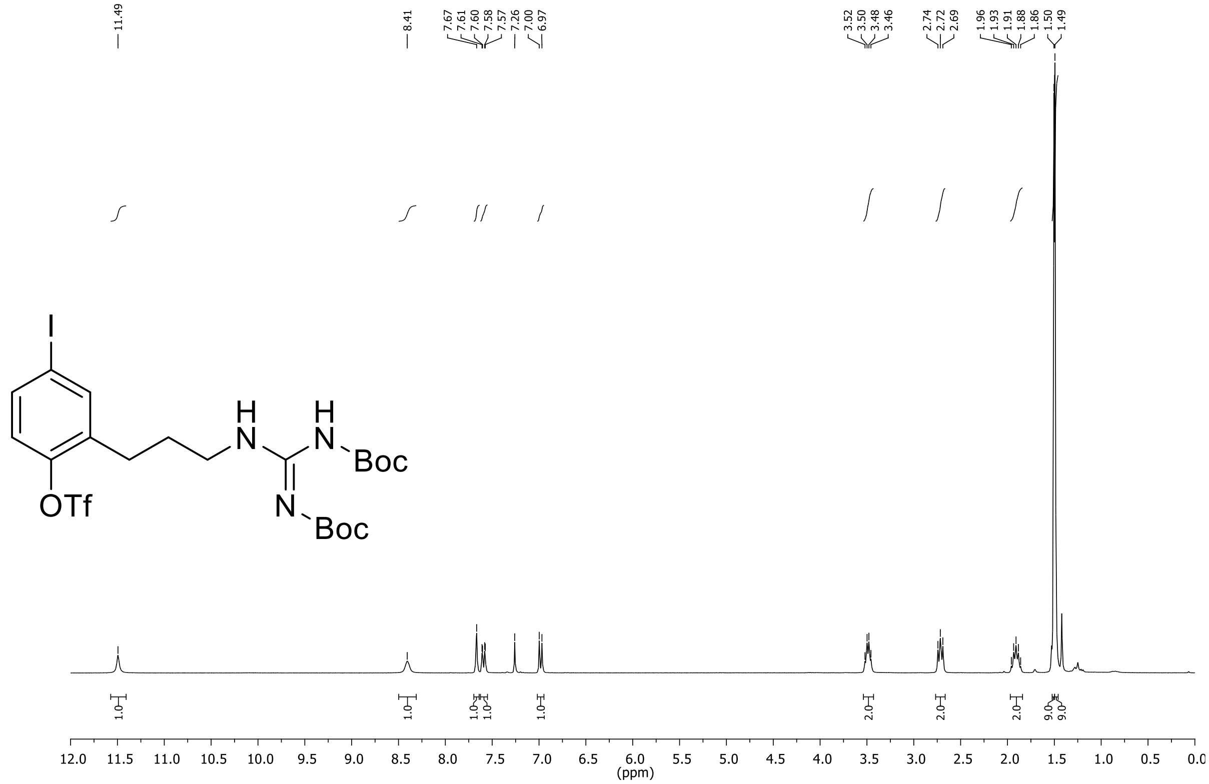

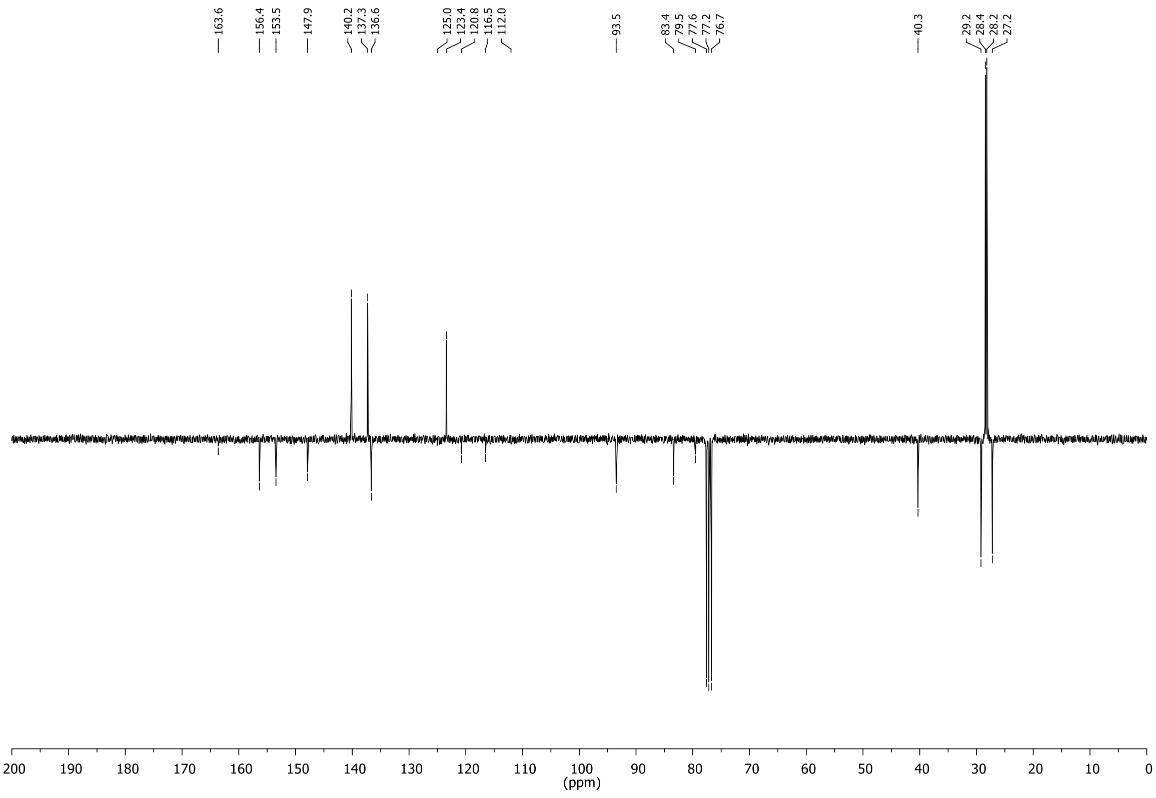

2-Methyl-4-(pyridin-3-yl)phenyl trifluoromethanesulfonate (**29**)

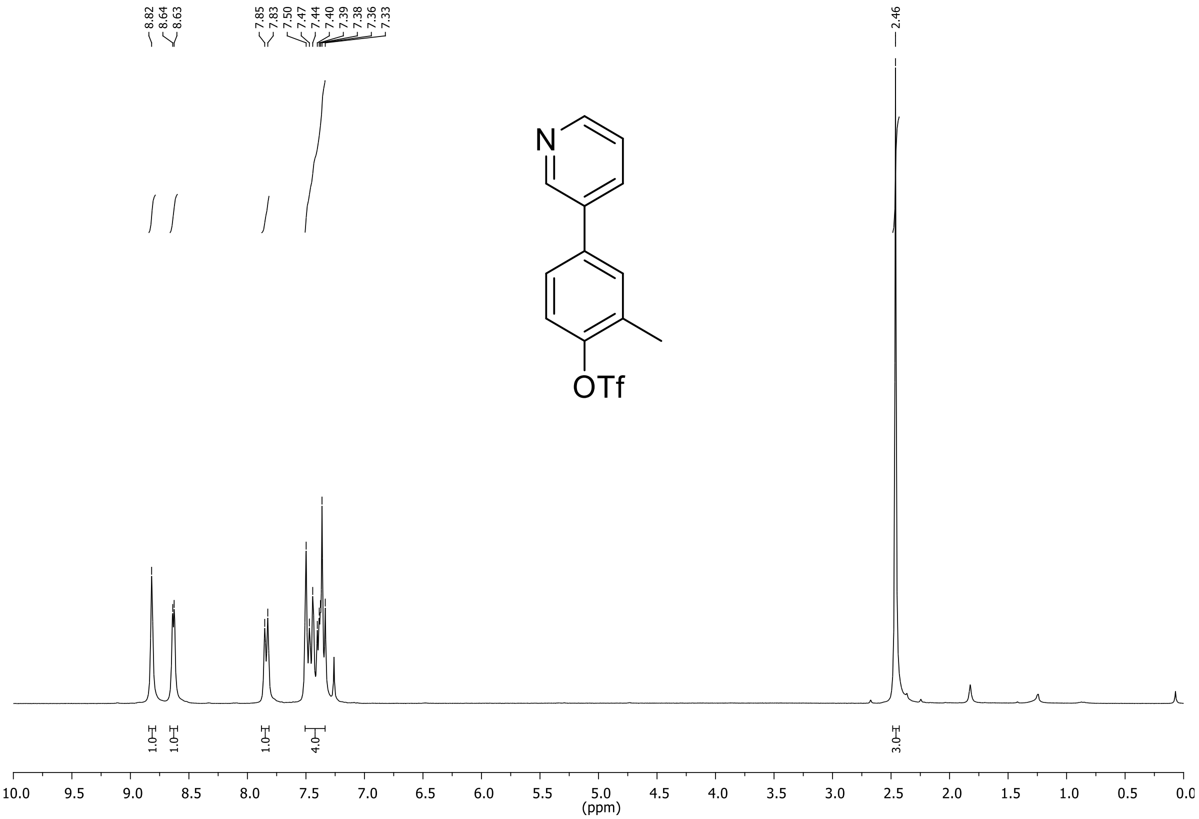

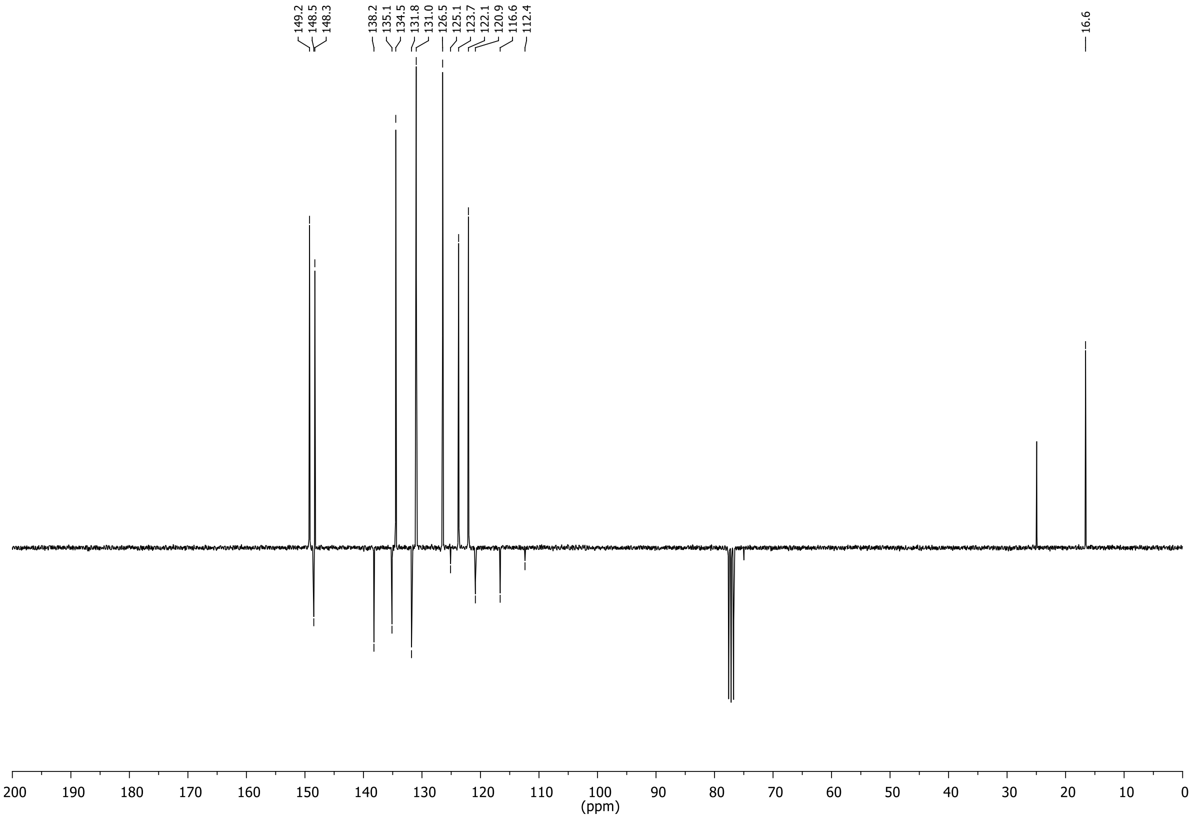

3,3'-(2-Methyl-1,4-phenylene)dipyridine (**30**)

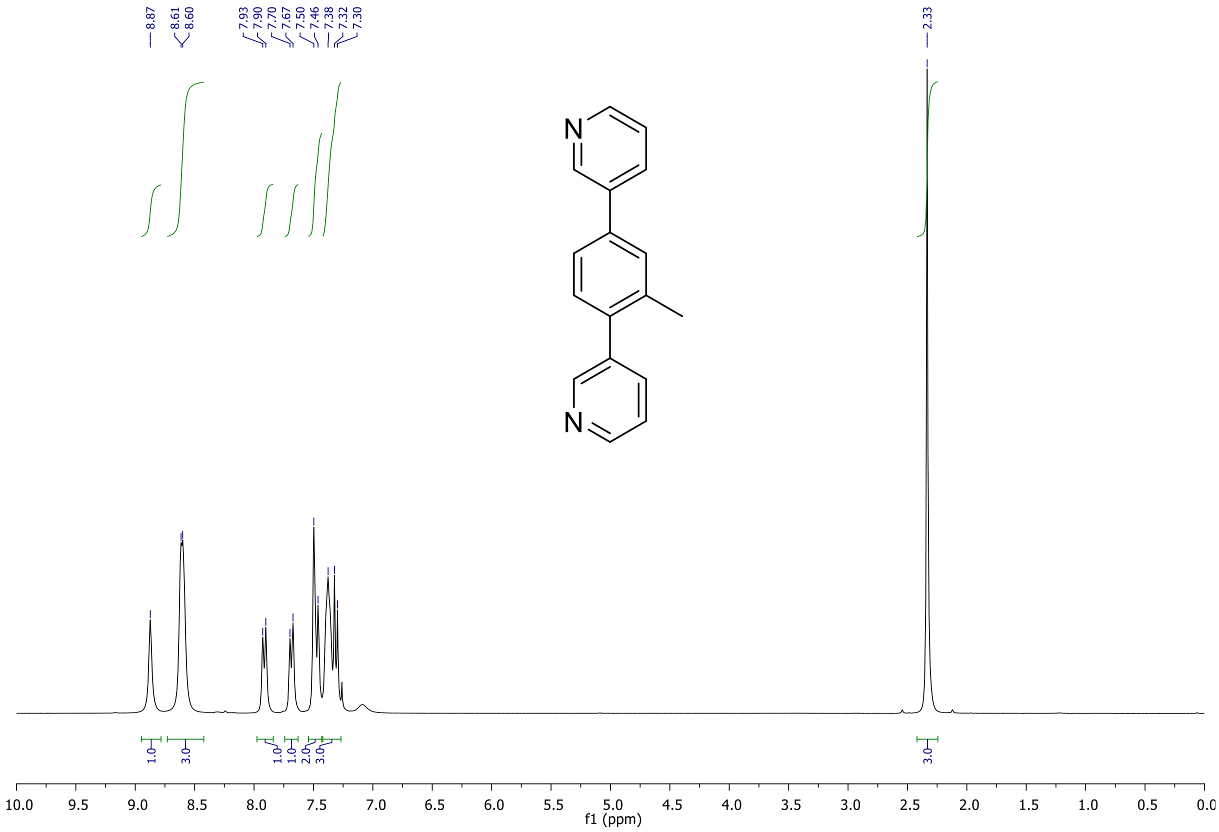

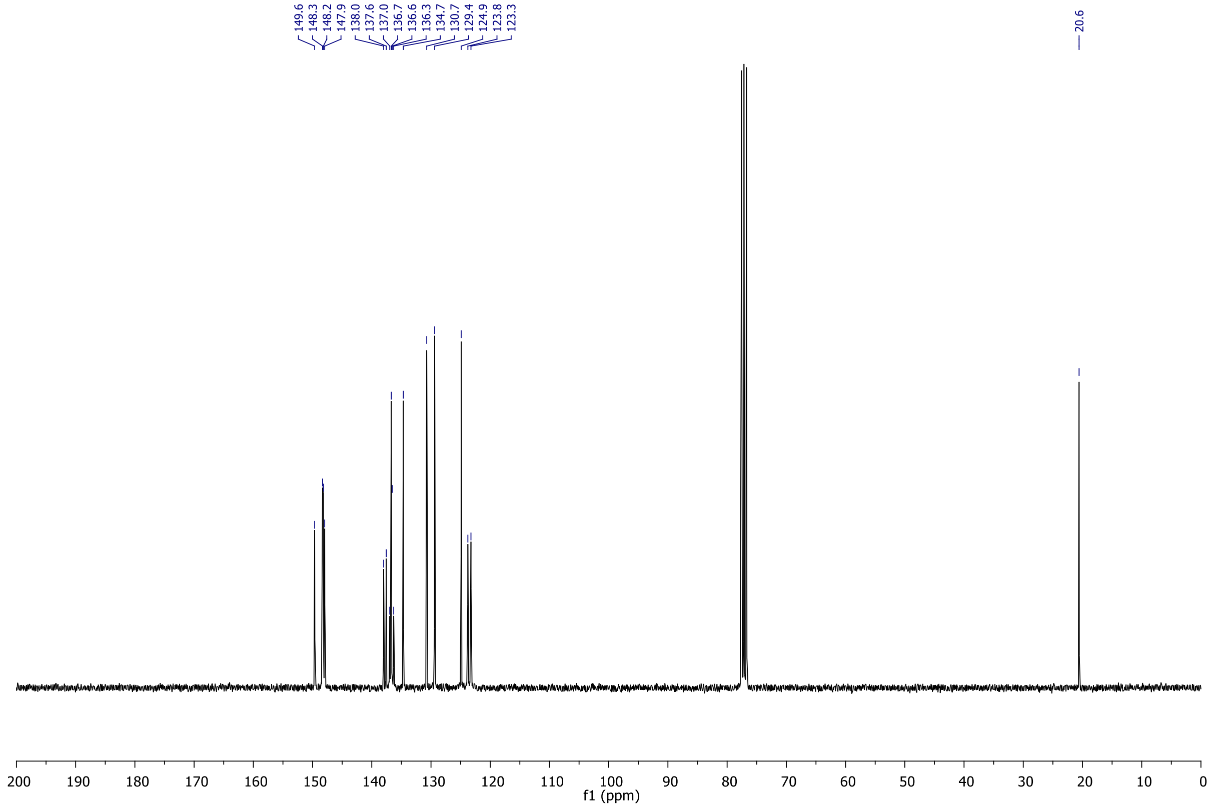

3-Bromo-5-isopropylpyridine (**31a**)

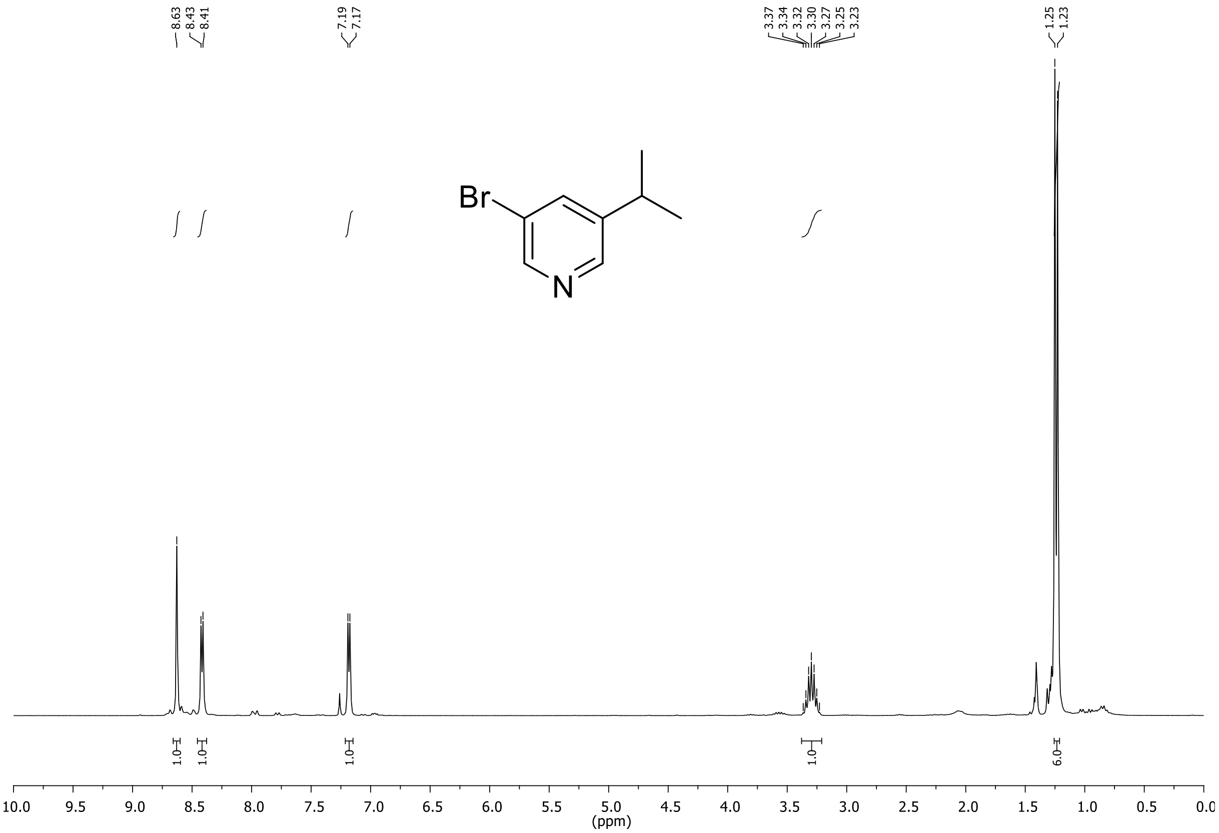

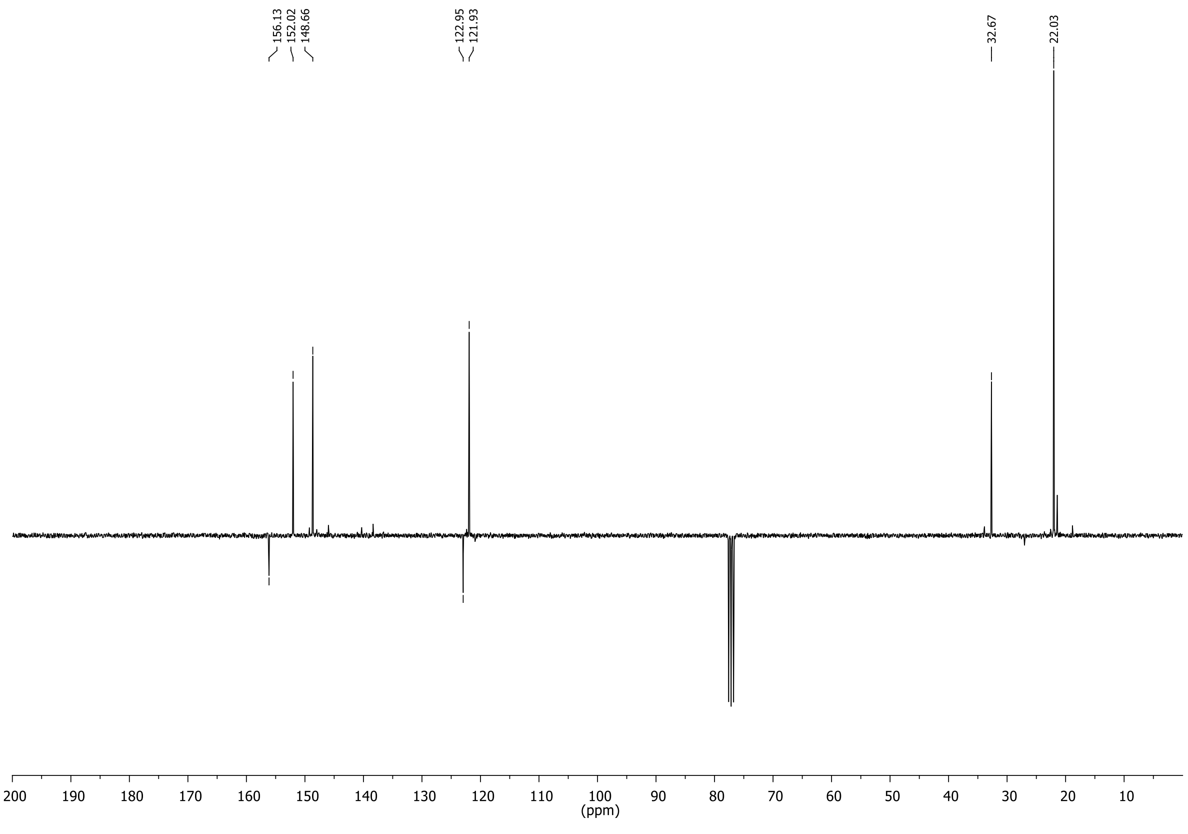

3-Isopropyl-5-(4,4,5,5-tetramethyl-1,3,2-dioxaborolan-2-yl)pyridine (**31**)

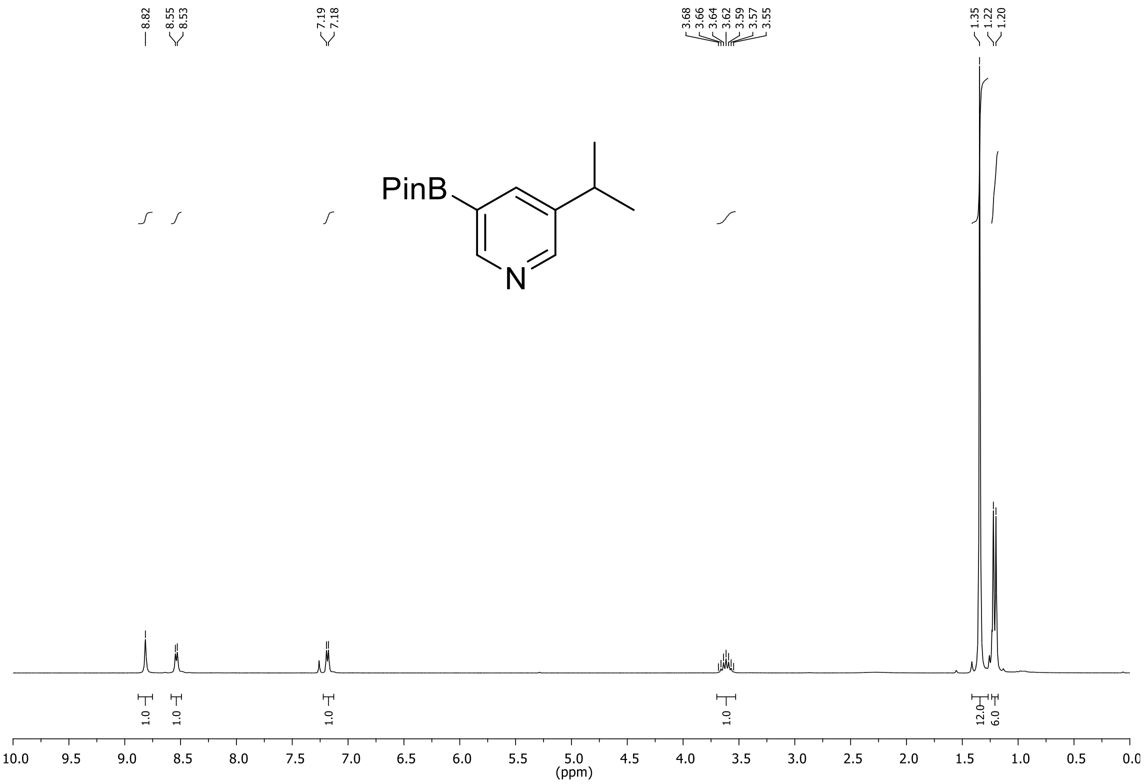

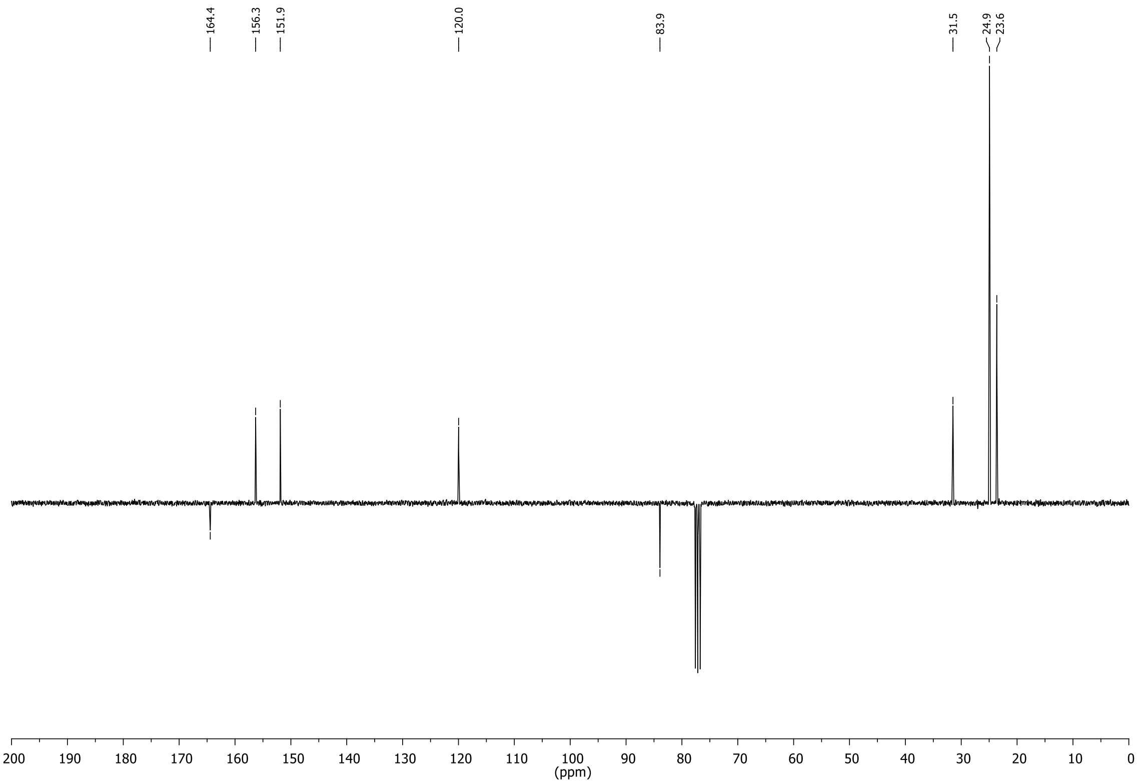

Gly-Met-Val (**32**)

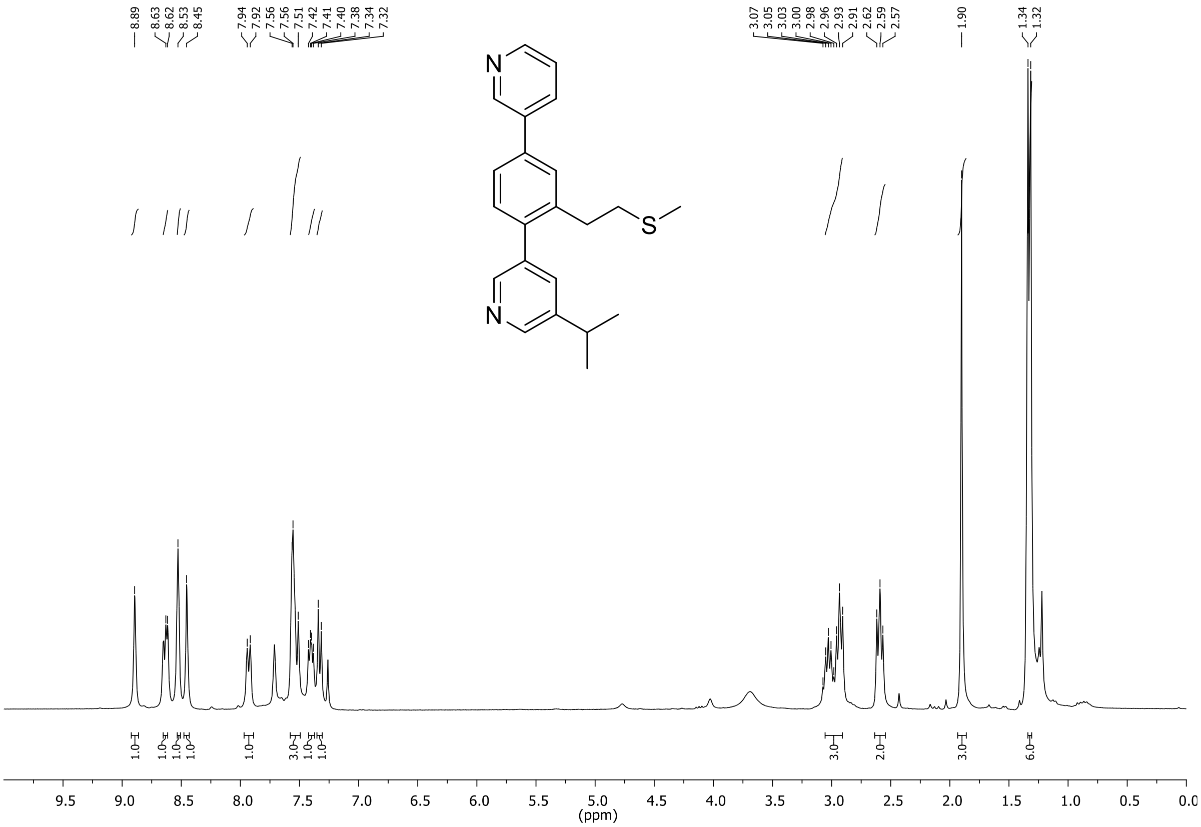

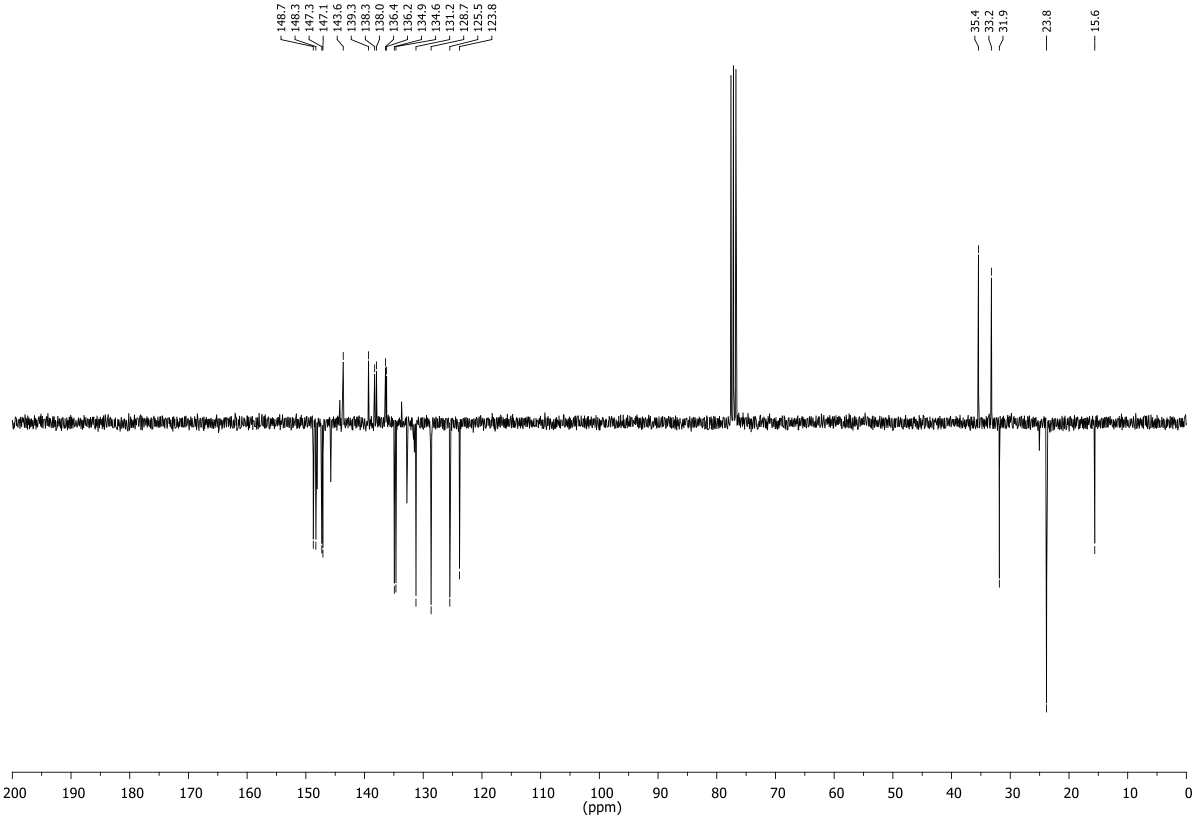

Methyl 2-(2-(5-isopropylpyridin-3-yl)-5-(pyridin-3-yl)phenyl)acetate (**33a**)

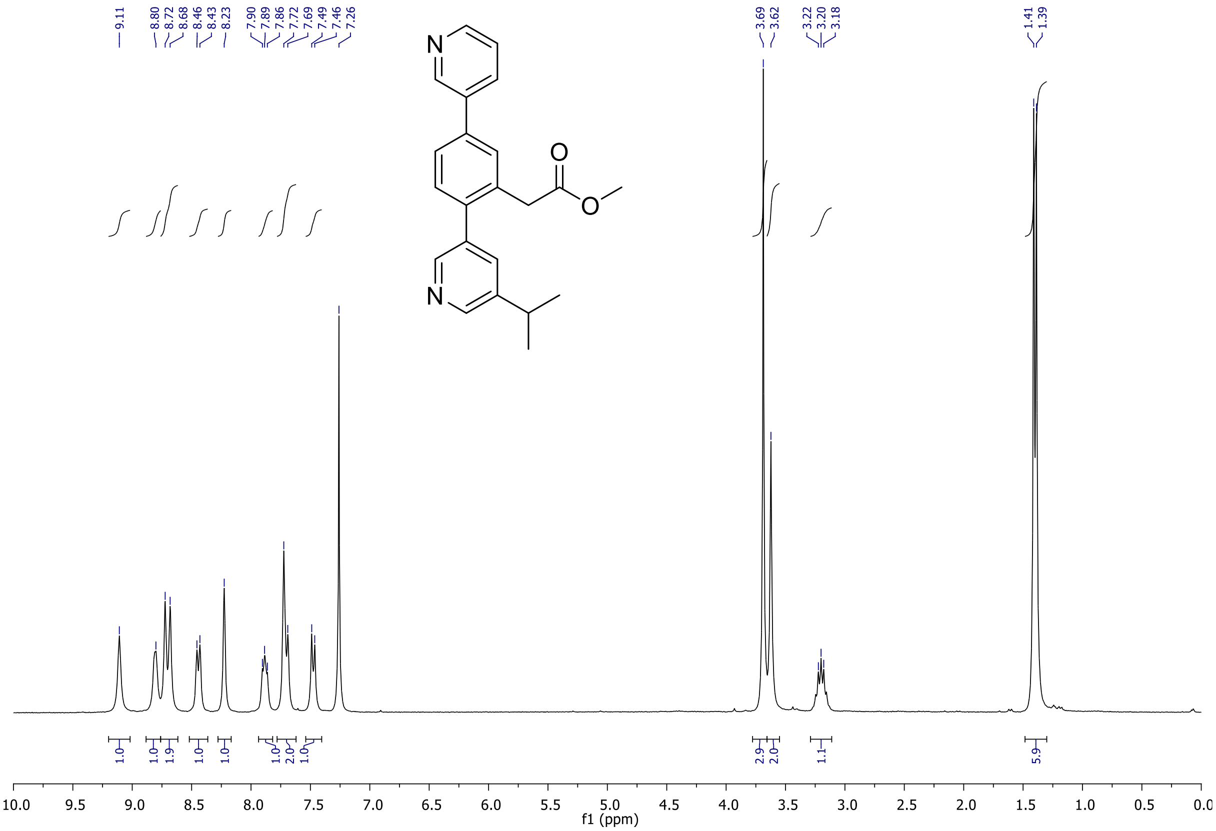

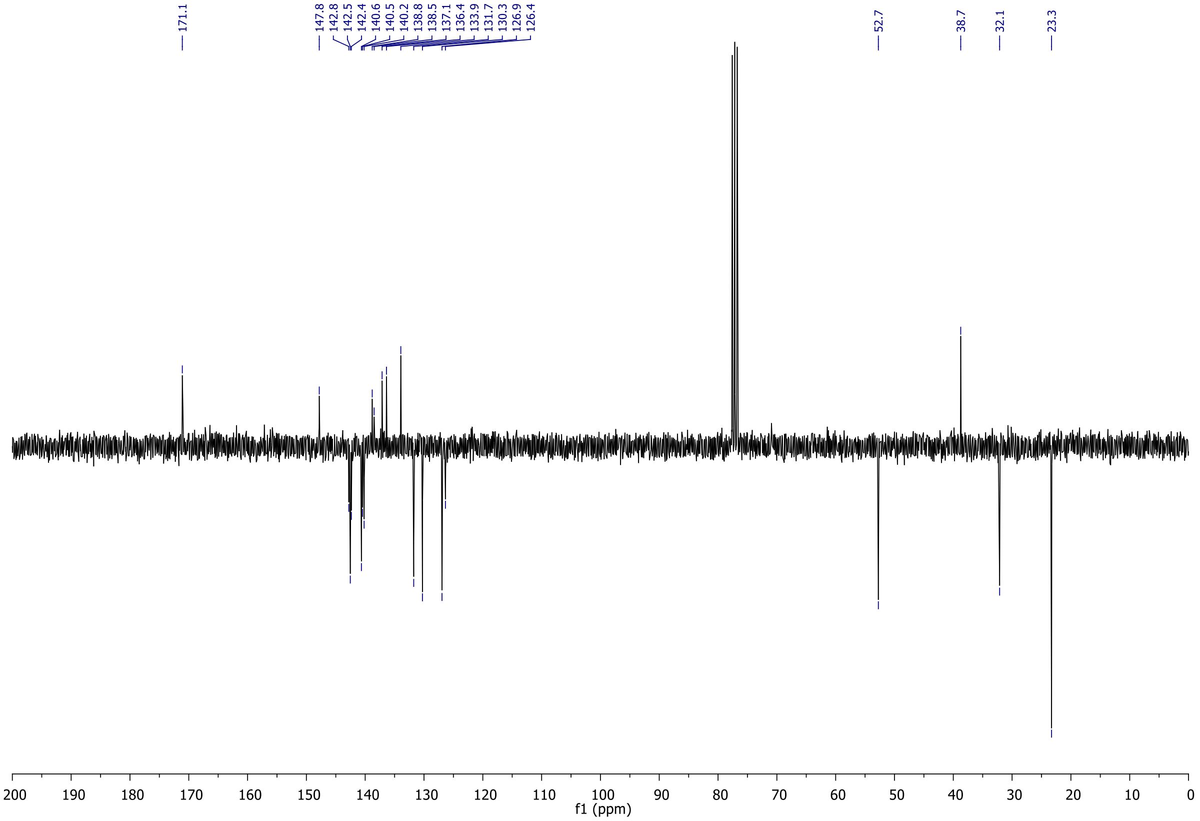

Gly-Asn-Val **(33)**

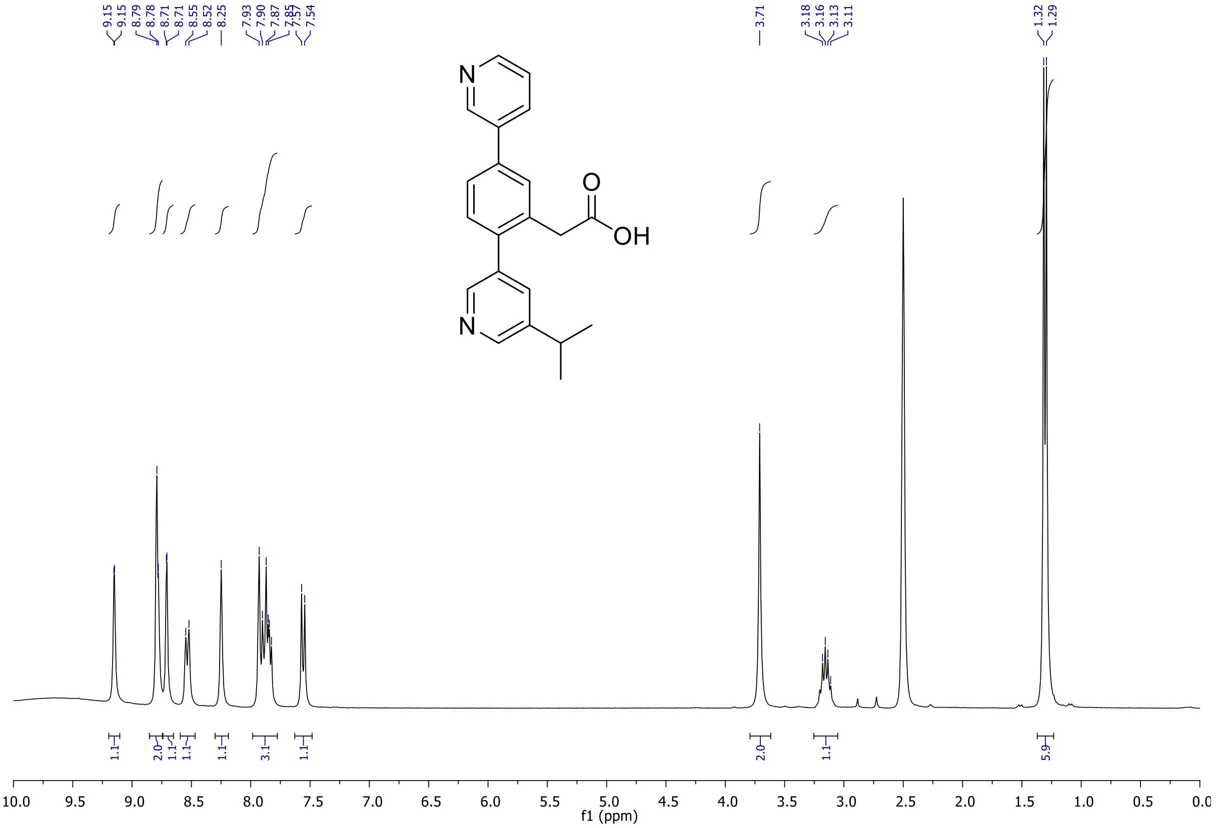

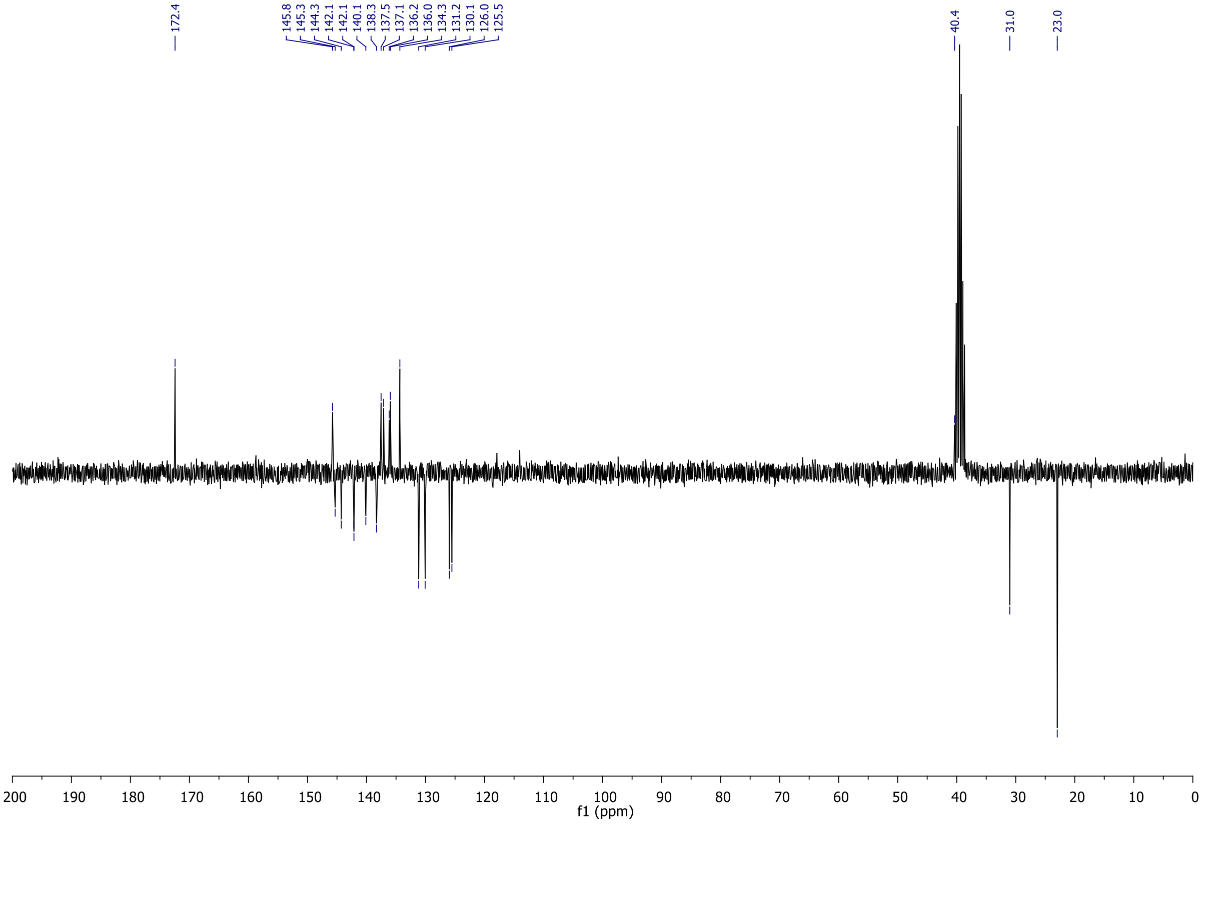

Gly-Tyr-Val **(34)**

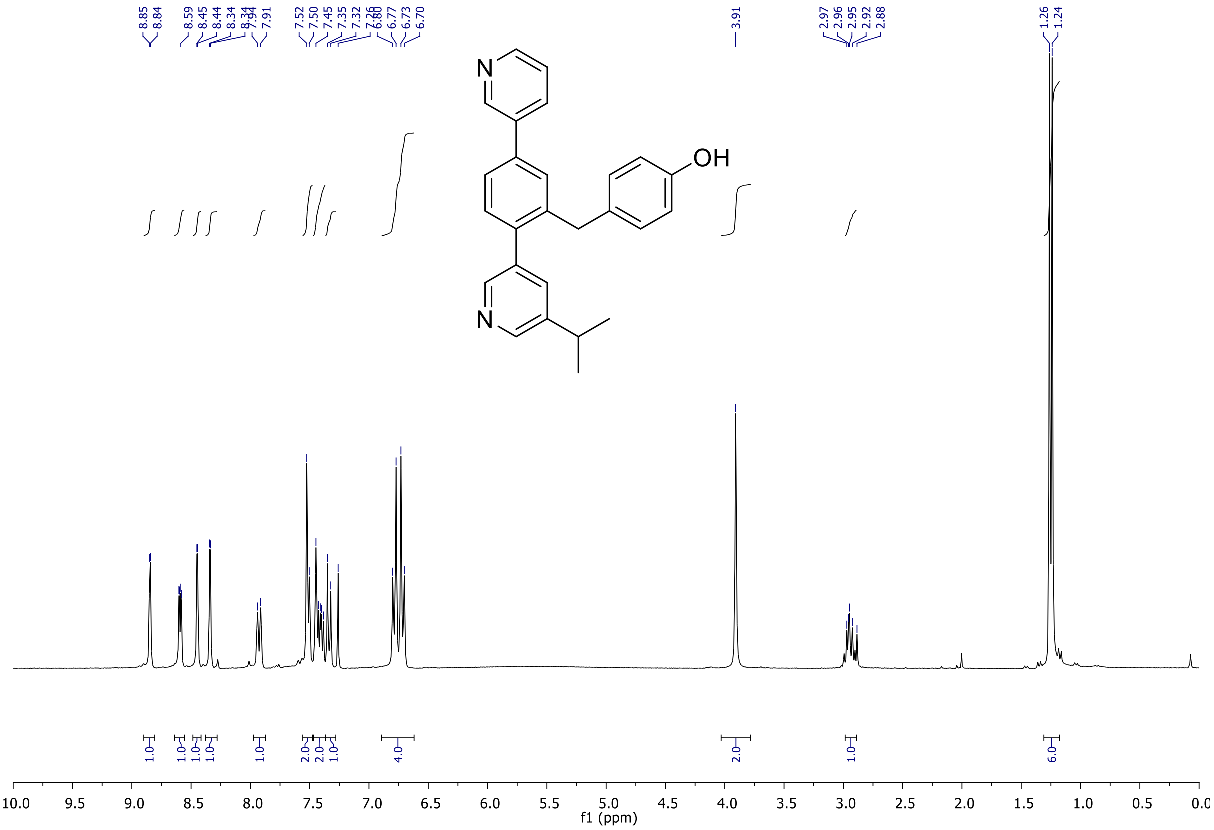

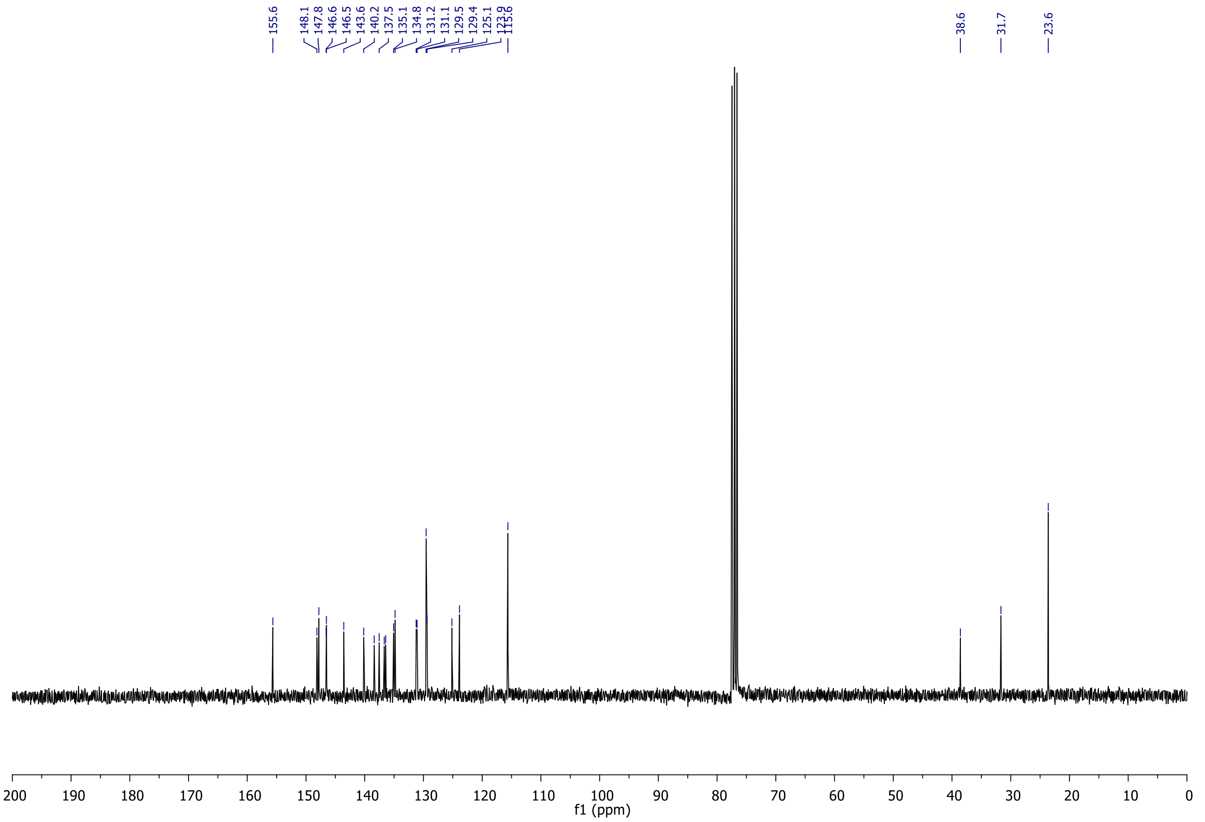

Gly-Trp-Val **(35)**

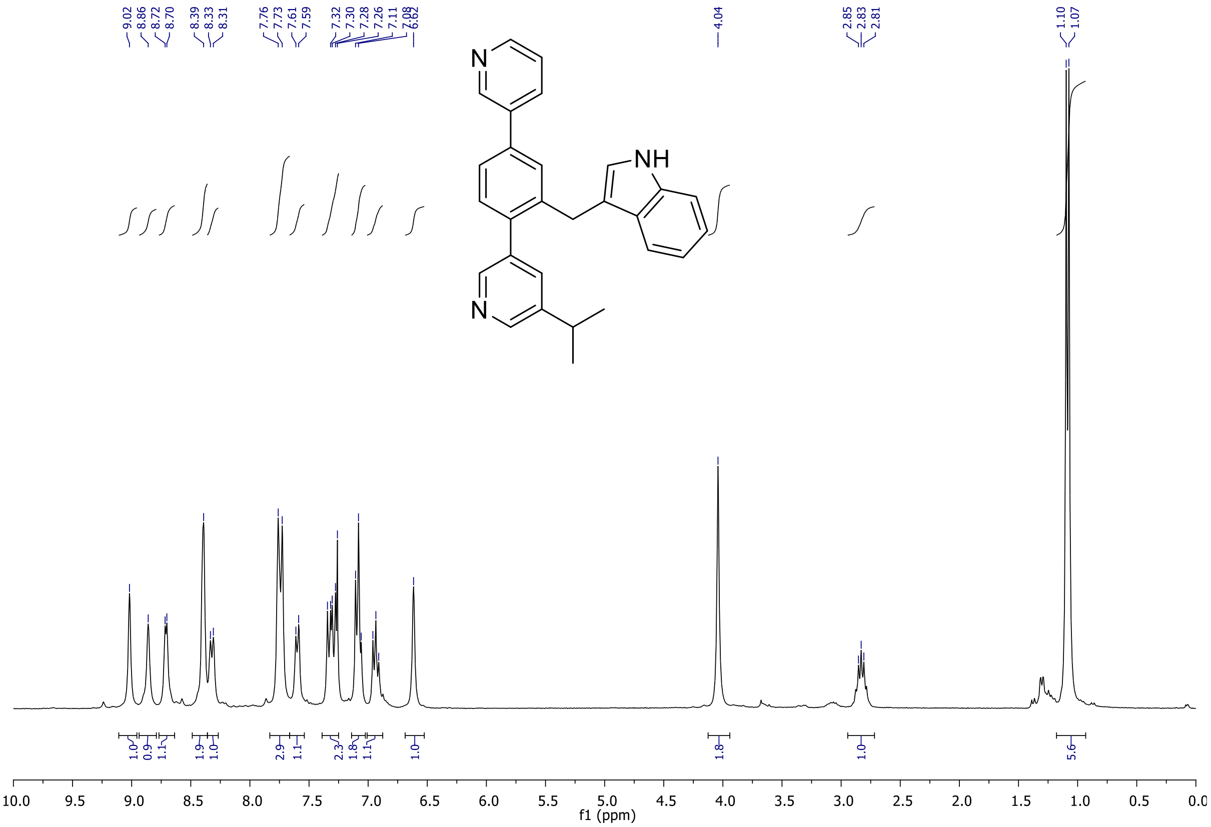

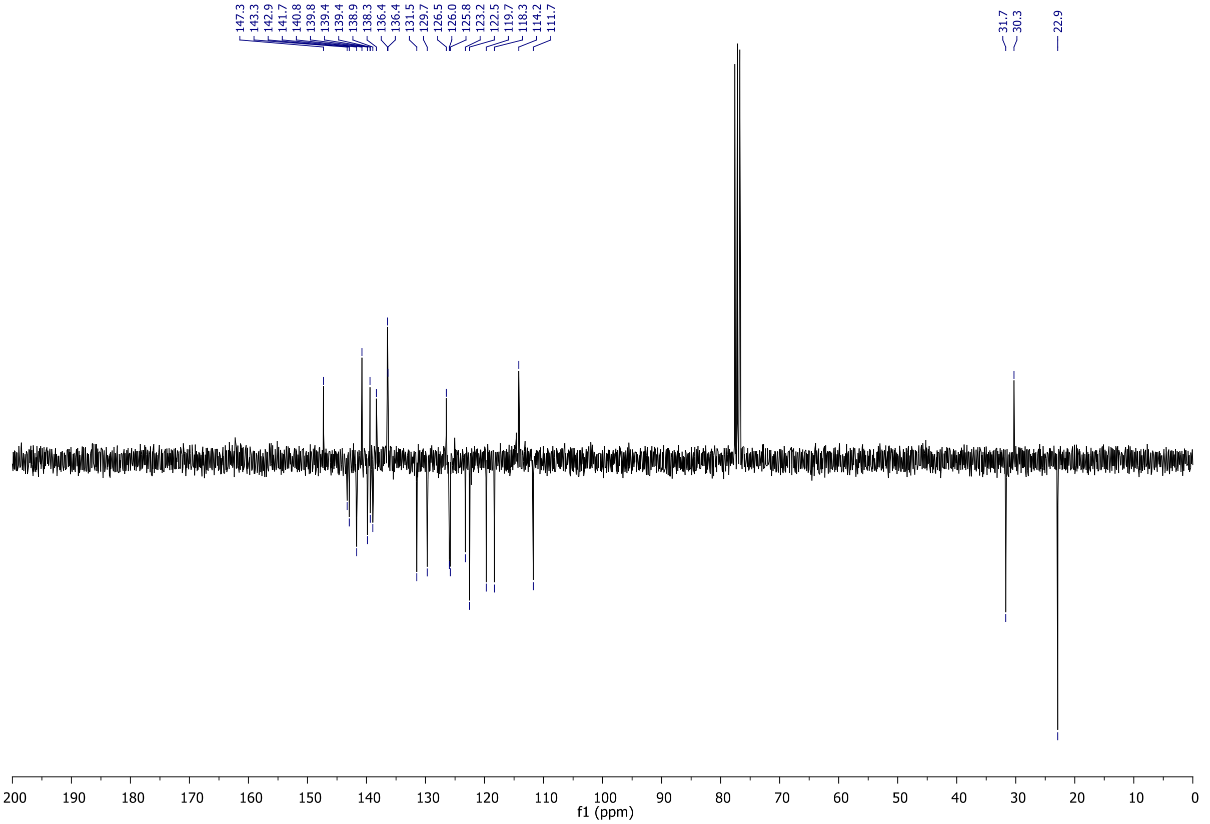

Supplement: Supplementary file 1 — Supporting Information [file EJOC-2022-0-s001.pdf]
